# Genome evolution in the fish family salmonidae: generation of a brook charr genetic map and comparisons among charrs (Arctic charr and brook charr) with rainbow trout

**DOI:** 10.1186/1471-2156-12-68

**Published:** 2011-07-28

**Authors:** Evan R Timusk, Moira M Ferguson, Hooman K Moghadam, Joseph D Norman, Chris C Wilson, Roy G Danzmann

**Affiliations:** 1 Department of Integrative Biology, University of Guelph, Guelph, Ontario, N1G 2W1, Canada; 2 Great Lakes Laboratory for Fisheries and Aquatic Sciences, Fisheries and Oceans, Sault Ste. Marie, Ontario, P6A 2E5, Canada; 3 Department of Zoology, University of Oxford, South Parks Rd., Oxford, OX1 3PS, UK; 4 Aquatic Research and Development Section, Ontario Ministry of Natural Resources, Trent University, Peterborough, Ontario, K9J7B8, Canada

**Keywords:** whole-genome duplications, salmonid fishes, pseudolinkage, recombination rates, segregation distortion, comparative synteny analyses, homeologies, duplicated genes

## Abstract

**Background:**

Salmonids are regarded as 4R derivative species, having experienced 4 whole genome duplication events in their ancestry. Many duplicated chromosome regions still share extensive homology with one another which is maintained primarily through male-based homeologous chromosome pairings during meiosis. The formation of quadrivalents during meiosis leads to pseudolinkage. This phenomenon is more prevalent within 5 of the 12 ancestral teleost linkage groups in salmonids.

**Results:**

We constructed a genetic linkage map for brook charr and used this in combination with the genetic map from Arctic charr, to make comparisons with the genetic map of rainbow trout. Although not all chromosome arms are currently mapped, some homologous chromosome rearrangements were evident between Arctic charr and brook charr. Notably, 10 chromosome arms in brook charr representing 5 metacentric chromosomes in Arctic charr have undergone rearrangements. Three metacentrics have one arm translocated and fused with another chromosome arm in brook charr to a make a new metacentrics while two metacentrics are represented by 4 acrocentric pairs in brook charr. In two cases (i.e., BC-4 and BC-16), an apparent polymorphism was observed with the identification of both a putative metacentric structure (similar to metacentric AC-4 = BC-4 and a joining of acrocentric AC-16 + one arm of AC-28 = BC-16), as well as two separate acrocentric linkage groups evident in the mapping parents. Forty-six of the expected 50 karyotypic arms could be inter-generically assigned. SEX in brook charr (BC-4) was localized to the same homologous linkage group region as in Arctic charr (AC-4). The homeologous affinities detected in the two charr species facilitated the identification of 20 (expected number = 25) shared syntenic regions with rainbow trout, although it is likely that some of these regions were partial or overlapping arm regions.

**Conclusions:**

Inter-generic comparisons among 2 species of charr (genus *Salvelinus*) and a trout (genus *Oncorhynchus*) have identified that linkage group arm arrangements are largely retained among these species. Previous studies have revealed that up to 7 regions of high duplicate marker retention occur between *Salmo *species (i.e., Atlantic salmon and brown trout) and rainbow trout, with 5 of these regions exhibiting higher levels of pseudolinkage. Pseudolinkage was detected in the charr species (i.e., BC-1/21, AC-12/27, AC-6/23, = RT-2p/29q, RT-12p/16p, and RT-27p/31p, respectively) consistent with three of the five 'salmonid-specific' pseudolinkage regions. Chromosome arms with the highest number of duplicated markers in rainbow trout are the linkage group arms with the highest retention of duplicated markers in both charr species.

## Background

Understanding the evolution of vertebrate genomes requires knowledge of the consequences of the whole genome duplications that have characterized their history [1]. Comparative studies suggest that the modern day assemblage of ray-finned fishes have descended from an ancestral grouping of fishes with 12-13 linkage groups [2,3]. All jawed vertebrates are hypothesized to have experienced two whole genome duplications (WGD) in their ancestry (2R duplication) with a third duplication (3R) in most extant teleosts [4,5]. Salmonid fishes have undergone one additional WGD (4R) between 25-100 MYR [6]. The presence of multivalents during meiosis, tetrasomic segregation at some loci, and the retention of large numbers of duplicated gene copies as syntenic clusters on homologous chromosome arms suggests that the genome duplication event was auto-polyploid in nature.

Tetrasomic segregation is expected to prevail as a result of quadrivalent formations during meiosis following a whole genome duplication event. The gradual decay towards modes of disomic segregation from the increasing formations of paired sets of bivalents during meiosis is expected through time [7]. Both modes of gamete segregation have been observed in species such as rainbow trout (*Oncorhynchus mykiss*) [7], and complete disomic inheritance has not yet been restored in any salmonid species [6,8].

Structural divergence of homeologous chromosomes into homologous chromosomes during the diploidization process is thought to occur through centric fusions between non-homeologous chromosomes [8]. The degree of divergence from the presumed acrocentric karyotype of the ancestral salmonid varies among present day species. Species characterized by Group A karyotypes (2N = 80, NF = 100) such as Arctic charr (*Salvelinus alpinus*) and brook charr (*Salvelinus fontinalis*) have more acrocentric than metacentric chromosomes while Group B species (2N = 60, NF = 104) such as rainbow trout have more derived karyotypes with greater numbers of metacentric chromosomes [9]. The Atlantic salmon is an exception with chromosome and arm numbers of 54-58 and 72-74, respectively leading to the most derived karyotype of all salmonids which is composed of a number of whole arm fusions [8].

Genetic linkage maps have been used to more fully investigate the patterns of chromosomal rearrangements that have taken place after the 3R and 4R WGD events. The ancestral linkage groups of ray-finned fishes share whole arm affinities with the homeologous chromosomal segments in Atlantic salmon and rainbow trout [10]. Comparisons among the genetic linkage maps of rainbow trout and Atlantic salmon [10,11] as well as the assignment of linkage groups to their specific chromosomes [12,13] have detected whole chromosome arm translocations (Robertsonian translocations) in the two species although ancestral chromosome arms have largely remained intact. In addition, species with more derived karyotypes (rainbow trout and Atlantic salmon) show greater sex-specfic differences in recombination rates compared to less derived species such as the Arctic charr [11]. It is still not entirely clear whether sex-specific differences arise due to elevated recombination in small, putative acrocentric, chromosomes or proportionally greater suppression of recombination in large metacentric chromosomes in males relative to females.

A more complete picture of genome evolution in salmonids requires a more detailed reference framework in which to evaluate chromosomal rearrangements. Initial studies have been based on comparisons between rainbow trout and Atlantic salmon to Arctic charr with its more basal karyotype. Unfortunately, the Arctic charr genetic linkage map is relatively incomplete relative to those of the more derived species resulting in a limited degree of comparison. The objective of the current research is to use an updated genetic linkage map for Arctic charr as a template to create a linkage map for brook charr, a second species of salmonid with a relatively basal karyotype. We predicted that individual brook charr linkage groups would share a high degree of homology to single Arctic charr linkage groups due to the apparent lack of major chromosomal rearrangements that have been observed in other salmonid species when comparative data has been utilized [13]. Furthermore, due to the more derived nature of the rainbow trout and Atlantic salmon karyotypes, it was expected a single metacentic linkage group in these species would share homology with at least two brook charr linkage groups. It was also expected that sex-specific differences in recombination rate in brook charr would be most similar to those observed in Arctic charr given their similar karyotypes.

## Results

### Brook charr map statistics

Of the 103 primer sets utilized, 26 amplified two polymorphic loci and five amplified three polymorphic loci. A total of 35 linkage groups were identified (Additional Files 1, 2, 3) (Table 1), 14 of which are represented by only a pair of loci. However, if the two instances of linkage group polymorphisms (i.e., BC-4 and BC-16; see below) are also counted then 37 linkage groups may be evident. Six of the 139 loci detected remain unlinked at a LOD threshold of 3.0 (see Additional File 1 for a list of all markers which have been mapped in at least one mapping parent). Following comparative mapping, it was ascertained that one of the unassigned markers (i.e., OMM3095) could localize to the RT-19p linkage group arm, and another marker OMM5146 may assign to the RT-31p linkage group arm. The RT-19p rainbow trout linkage group arm does not currently possess major homology to any of the other brook charr linkage group arms, suggesting that this may be representative of separate linkage group arms in brook charr. Four of the unassigned markers are duplicates (BX073647/i, CA368462/i, OMM1195/ii and BHMS417/iii) with unknown affinities, while the singleton marker SalD39SFU may be tentatively assigned to BC-25 given the homology of this marker to the AC-25 linkage group. Given that not all brook charr chromosomes are currently represented by two or more markers, the inclusion of OMM3095 as being representative of a single linkage group, or part of a linkage group arm would indicate that perhaps only 4 of the expected 42 linkage groups (8 pairs of metacentrics and 34 pairs of acrocentrics) [8,14], are not represented in this study.

**Table 1:** Comparison of putative linkage groups in Arctic charr and brook charr based upon comparative alignments of homologous marker segments to the rainbow trout genome (see Additional File 9 for these arm assignments)

Arctic charr linkage group	Chromosome Type*	Brook charr linkage group	Chromosome Type*	No. of BC arms detected
AC-1	M	BC-1	M	2
AC-3	M	BC-3	M	2
AC-4	M	BC-4**	M	2
AC-5	A	BC-5	A	1
AC-6	A?	BC-6	A?	1
AC-7	A	BC-7	A	1
AC-8	M	BC-8	A?	1
AC-9	A	BC-9	A	1
AC-10	A	BC-10	M^1^	2
AC-11	A	BC-11	A	1
AC-12	A	BC-12	A	1
AC-13	M	BC-13a	A	1
		BC-13b	A	1
AC-14	A	BC-14	A	1
AC-15	M?	BC-15	M?	2
AC-16	A	BC-16**	M^2^	2
AC-17	A	BC-17	M^3^?	2
AC-18	M	BC-18	M	2
AC-19	M?	BC-19a	A	1
		arm b in BC-17	see^3^	
AC-20	M	BC-20a	A	1
		BC-20b	A	1
AC-21	A	BC-21	A	1
AC-22	A	BC-22	A	1
AC-23	M?	BC-23b	A	1
		arm a in BC-35	see^5^	
AC-24	A	BC-24	A	1
AC-25	M	BC-25	M?	2
AC-26	A	arm in BC-30	see^4^	
AC-27	A?	BC-27	A	1
AC-28	M	BC-28a	A	1
		arm b in BC-16**	see^2^	
AC-30	A	BC-30	M^4^	2
AC-31	A	?		
AC-32	A	BC-32	A	1
AC-33	A	?		
AC-34	A	BC-34	A	1
AC-35	A	BC-35	M^5^	2
AC-36	A	BC-36	A	1
AC-37	A	BC-37	A	1
AC-39	A	arm in BC-10	see^1^	
AC-43	A	BC-43	A	1

Total No. Arms	49		total arms	46

* Chromosome is defined as either metacentric (M) or acrocentric (A).

** Polymorphisms were detected with both BC-4 and BC-16 linkage groups. BC-4 appears to form a metacentric linkage group in females, but a singleton position was detected with SSOSL32 in HL7 male, suggesting the formation of two acrocentrics. BC-16 markers appear to form a larger linkage group in the HL7 female (= metacentric structure), but only form two smaller linkage group clusters in the HL3 female (= 2 acrocentric linkage groups?).

Superscripts ^1, 2, 3, 4, 5 ^indicates that two Arctic charr chromosome arms are part of a single brook charr metacentric chromosome. see^1-5 ^designates which Arctic charr chromosome arm appears to be part of the brook charr metacentric having the same superscript numeral.

Although 8 metacentric linkage groups are expected in brook charr [8,14], up to 11 possible metacentric configurations were tentatively identified in this study. However, within two of these metacentric configurations (i.e., BC-4 and BC-16) intraspecific polymorphisms in linkage were observed. In BC-16 two apparent acrocentric clusters evident in one mapping female were joined in the other mapping female. Also, the marker SSOSL32 appears joined to other BC-4 markers in the female mapping parents, but appears as a separate unassigned singleton marker in the HL7 male mapping parent. This suggests that two acrocentric linkage groups may exist in certain males (designated BC-4a and BC-4b). Furthermore, for three of the putative metacentric assemblages (i.e., BC-15, -17, and -25), the available data do not allow us to unequivocally assess these linkage groups as being metacentric. Markers assigned to both the rainbow trout RT-10p and RT-10q linkage group arms do assign to BC-15 suggesting a metacentric structure for this linkage group. This provides stronger support that the linkage group BC-15 in this study is metacentric. BC-17 possesses a maker homologous to both the AC-17 and AC-19 linkage groups suggesting that a possible fusion of two linkage group arms may have occurred in brook charr (Table 1). However, as mentioned previously, only a single marker homologous to AC-25 (i.e., SalD39SFU) was genotyped, and identified as a singleton. Homeologous affinities to this linkage group and both BC-4 and BC-22 linkage groups cannot be clearly resolved as all three linkage groups contain a region that amplifies marker Ssa0080BSFU (based on Arctic charr homologies). In the current study duplicates of Ssa0080BSFU appear to map to BC-4/22. The linkage of Ssa0080BSFU with OkeSLINRA tentatively places this affinity on BC-22, rather than to BC-25, given that only a single copy of the OkeSLINRA gene has been mapped to AC-22 in Arctic charr.

Map lengths were substantially different between the sexes. The female and male HL3 maps, which are the most complete, span 4.43 and 2.04 Morgans, respectively. A complete female map is expected to be roughly 25 Morgans, assuming approximately 50 cM per chromosome arm pair as brook charr contain 50 chromosome arm sets [8]. The combined brook charr female map covers a total of 5.548 Morgans and thus the current map likely represents only about 20% of the genome. We also provide updated marker information for the composite Arctic charr female genetic map in this study, which increases the coverage in this species to 20.589 Morgans. This adds an additional 275 markers to the existing male and female Arctic charr genetic map [15](See Additional File 4) and increases the map size to 620 markers.

### Pseudolinkage

Three instances of possible pseudolinkage were evident in the brook charr maps for male HL7. Three markers assigned to BC-21 in the female maps, were localized to the BC-1 linkage group suggesting a possible pseudolinkage affinity between BC-1/21 in this male. Also, marker OMM5007 maps to BC-20a, but is localized to BC-43 in the other mapping parents. This arrangement is intriguing, as BC-43 is homologous to the RT-9p linkage group arm, while BC-20a is homologous to the RT-9q arm. In addition, a small segment of BC-15 possessed duplicate markers of OMM1197, suggesting a possible BC-10/15 pseudolinkage. These latter two instances are only based upon single markers, however, and therefore may also represent tandem repeats.

### Linkage group polymorphisms in brook charr

Two different arrangements for the markers on BC-16 were detected in the two female parents. Within the HL3 female, markers located in regions homologous to either AC-16 and AC-28b were localized to separate single linkage groups (LOD = 3.0 clusters) representative of their configuration in Arctic charr. Conversely, these markers were joined into a single LOD = 3.0 cluster in the HL7 female parent suggestive of a metacentric chromosome. The two clusters detected within the HL3 female may simply represent unjoined BC-16 groupings given the low numbers of markers genotyped, While these two arms appear to be separate linkage groups in Arctic charr, they also form a metacentric linkage group in rainbow trout (i.e., RT-8). If these two regions do in fact represent separate linkage groups in some brook charr females, then this could represent a case of female-specific pseudolinkage. Such a configuration would, however, be extremely unlikely given that it would be female based, and most importantly, involve linkage group arms that are unrelated ancestrally. An interpretation that this represents a polymorphism involving either a metacentric or two separate acrocentrics is more likely. We have tentatively identified both of these linkage group arms as BC-16 but denote the second HL3 cluster as BC-16b in Additional File 1.

Linkage group BC-4 was made up of two clusters of markers in the HL7 male parent. Markers homologous to AC-4 were joined in the female mapping parents, while the linkage group region marked by SSOSL32 was unlinked in the HL7 male. This same marker was linked to other markers on BC-4 in the female mapping parents. Although SSOSL32 is duplicated in Arctic charr (i.e., AC-4/25 homeologies) this primer set amplifies only a single locus in both parents from the HL7 family.

### Recombination rate comparisons

Significant differences in recombination rate were detected both within and between sexes (Additional File 5, 6). Average female: male recombination ratios were 3.47:1 for HL3 (N = 18) and 2.19:1 (N = 18) for HL7. For all but one marker interval (OMM5102/ii and BHMS465/i in HL7F vs HL7M and HL3F vs HL7M), higher recombination rate was detected in the female relative to the male mapping parent (Additional File 5). In addition, average female recombination rate was significantly higher than that of the males in both interfamily comparisons (i.e., HL3F vs HL7M and HL7F vs HL3M) (Additional File 5). Due to the paucity of markers genotyped in the LN4 mapping family, conserved syntenic blocks of markers were not detected and therefore this family was not included in the comparisons.

In same-sex comparisons, recombination rate was significantly higher in the HL3 female relative to the HL7 female (HL3F:HL7F = 1.49:1df, N = 20, *χ*^2 ^= 8.81), while average recombination rate did not differ between the HL3 and HL7 males (HL3M:HL7M = 0.8736, N = 27, *χ*^2 ^= 1.22) (Additional File 6). In the female comparisons, significant differences were present for the intervals tested on two linkage groups, BC-16 and BC-18, with the HL3 female showing higher recombination on all the intervals with significant differences. The greatest differences were evident on BC-16 where 13.3 cM separated two markers (CA060381 - BX299451), and 11 cM separated two additional markers (OMM5091 - OMM1195) in female HL3. In the HL7 female, the markers in both marker groups were linked to one another in a zero recombination cluster.

### Segregation distortion

Prior to Bonferroni correction, 33 loci representing nine linkage groups and one unlinked marker, did not segregate according to 1:1 Mendelian expectations (p < 0.05 in single G-tests) (Additional File 7). However, several of these markers were missing a large proportion of their genotypes (i.e., genetic markers where the parents are heterozygous for identical alleles, leading to uninformative phases in the progeny when they are also heterozygous), and were therefore excluded. When considering only the remaining 25 loci, nine instances of significant segregation distortion were detected on BC-16 alone among the parents tested. Only one unlinked marker BHMS417/iii remained significant following Bonferroni correction.

### Putative homologies (Salvelinus species combined and rainbow trout)

Marker assignments to the various brook charr linkage groups along with their comparative homologous locations within the Arctic charr and rainbow trout maps facilitated the tentative identification of 46 out of 50 expected linkage group arms in brook charr. Homologies to all Arctic charr linkage groups were evident, with the exception of two (AC-31 and AC-33) (Table 1). Similarly, the assessment of linkage groups AC-6/BC-6 and BC-19 as being acrocentric in structure is tentative, as there are single marker homologies to two separate chromosome arms in the rainbow trout genome. However, as previously mentioned, BC-17 does not appear to be homologous to one of the AC-19 linkage group arms, which suggests that an arm fusion between AC-19 and AC-17 homologous chromosomes has occurred in brook charr (= the designated BC-17 linkage group). This would then indicate that the remaining linkage group designated BC-19, is acrocentric. This could also be the result of some type of pseudolinkage affinity, or may represent a small translocation region between these two linkage groups. As mentioned above, the lack of known homeology affinities between BC-17 and -19 makes the possibility of pseudolinkage affinity less likely. More marker information is needed to resolve this.

**Figure 1:**
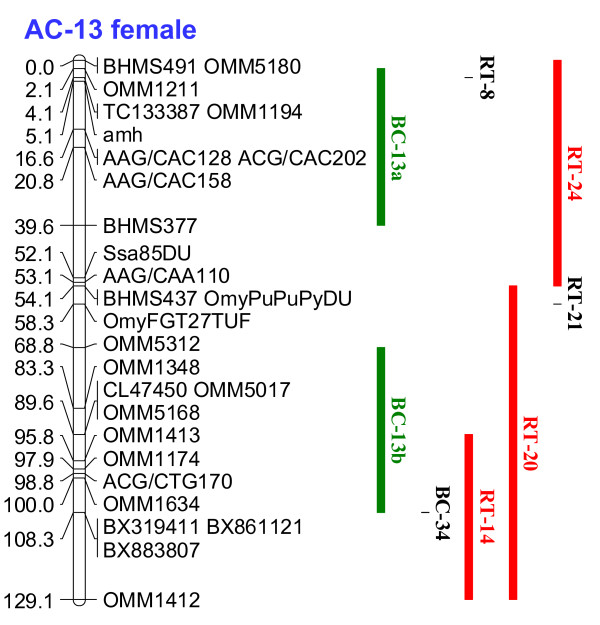
**Arctic charr linkage group 13 depicting homologous marker affinities in brook charr and rainbow trout**. Conserved synteny blocks spanning the homologous regions are shown in green (Brook charr) and red (rainbow trout).

An example of how cross homology assignments can be made is shown in Figure 1, using Arctic charr linkage group AC-13, which has a putative metacentric structure. This linkage group has homologies to the RT-24p linkage group arm, as well as to the RT-20q linkage group arm, which shares homeology with the RT-14p arm. In brook charr, homologous markers in this region are evident in two separate linkage groups, which are tentatively identified as acrocentric in structure. They are designated as BC-13a and BC-13b. The complete comparative homology maps among brook charr, Arctic charr, and rainbow trout, using the composite Arctic charr female map as the template, are shown in Additional File 8. Furthermore, cross assignments between all the extant *Salvelinus *linkage group arms to those currently described in rainbow trout [10,12] could be made for all chromosome arms except for RT-17q, RT-19p, RT-20p, and RT-25p (See Additional File 9).

### Putative homeologies

**Figure 2:**
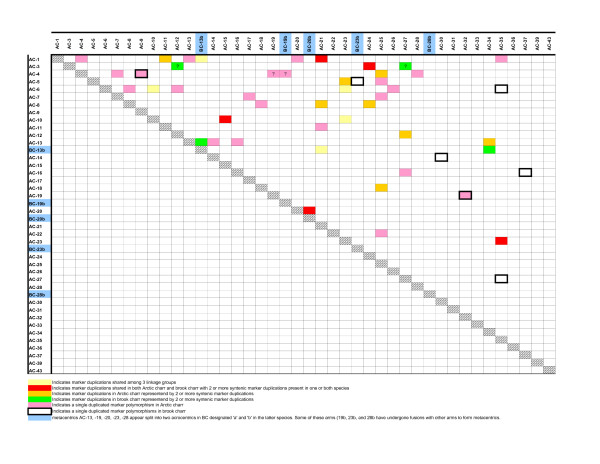
**Putative 4R homeologous linkage group affinities within Arctic charr and brook charr**. See the figure legend for explanation of the coloured blocks.

Based on the detection of 26 duplicated microsatellite loci and five primer pairs that amplified three copies, 15 putative homeologous linkage groups were identified in brook charr (Figure 2 and Table 2). In addition three duplicated microsatellite markers (BHMS417, BX073647, and CA368462) have one duplicated copy that is a singleton in the current map, while several markers may represent tandem duplications within linkage groups or pseudolinked markers that were detected (BHMS417, CA376300, Sal9UoG, OMM1197 and OMM5155). In combination with the duplicated homeologous linkage group blocks identified in Arctic charr (Figure 2), comparative mapping to the genome of rainbow trout facilitated the identification of 20 shared larger synteny block homeologs, along with two regions with smaller overlap (Table 3). Three markers spanning BC-1 and BC-21 linkage groups were joined at LOD = 3.0 threshold of clustering within the HL7 mapping parent suggesting an apparent pseudolinkage for these two linkage groups in brook charr. There is also a possible single marker pseudolinkage between BC-10 and BC-15, as previously mentioned, although more markers are needed to confirm this latter case.

**Table 2:** Putative homeologous linkage group affinities detected in brook charr

Putative homeologues^1^	Duplicated markers
BC-6 and BC-35	OMM5000
BC-14 and BC-30	OMM3015
BC-16 and BC-37	OMM5014
BC-16 and BC-16 and UL	BHMS417
BC-13a and BC-13b	BHMS377, OMM5312
BC-3 and BC-24	BHMS465, OMM5102
BC-3 and BC-3 and BC-12/-27	Sal9UoG
BC-3 and BC-12/-27	OMM5161
BC-25b and UL	BX073647
BC-5 and BC-23b	OMM1372
BC-8 and UL	CA368462
BC-1 and BC-1	OMM5155
BC-1 and BC-21	BX311884, BX087644
BC-1 and BC-21 and BC-13b	OMY21INRA
BC-27 and BC-35	OMM1263
BC-13b and BC-34	BX319411, BX861121
BC-32 and BC-19a	BX870052
BC-10 and BC-15	Omi30TUF, OmyRGT2TUF
BC-4 and BC-9	TC126859
BC-22 and UL	Ssa0080BSFU
BC-15 and BC-15^2^	OMM1197
BC-20a and BC-20a	CA376300
BC-20a and BC-20b	BX890355, OMM5019

^1 ^UL denotes that one of the duplicated copies is currently unlinked to any described brook charr linkage groups.

^2 ^appear to represent pseudolinked markers.

**Table 3:** Comparative homeologous linkage group affinities between *Salvelinus *species and rainbow trout linkage groups^1^

*Salvelinus *linkage groups	Rainbow trout linkage groups	*Salvelinus *linkage groups	Rainbow trout linkage groups	*Salvelinus *linkage groups	Rainbow trout linkage groups
1/11	12q/29p	1/21	2p/29q	3/24	7q/15p
5/23	1/19q/9c/20c	10/15	10q/18	4/9	14q/25q
4/25a	11/12q/26	4/19	27c/?	6/23	27p/31p
6/8	3p/27p	7/17	11/12q/26^2^	8/18	17p/22p
8/21	2p/29q	8/24	7q/15p^3^	12/27	12p/16p
13/34	14p/20q	13/16	8q/24p	20a/20b	2q/9q
18/25	6p?/30	19/32	13/23q	22/25	11/12q/26
23/35	27p/31p				

^1 ^Rainbow trout linkage group arms are designated as short (p), long (q), or centromeric (c) according to Danzmann et al. 2008 and Phillips et al. 2006. If a designation suffix is not indicated following the rainbow trout linkage group it is representative of a single acrocentric chromosome arm. Note: Linkage group RT-7 is depicted in an inverted orientation in Danzmann et al. (2008)

^2 ^Sal-7/17 homologies are mostly synonymous with a larger syntenic block of RT-21. The homeolog for this grouping in not known at present.

^3 ^appears to be a small region of synteny.

### Sex linkage

We tested markers localized to linkage groups BC-18 (homologous to AC-18), and BC-4 (homologous the Arctic charr sex linkage group AC-4), as being the most likely genomic locations to possess the sex determining region in brook charr. The sex-linked marker Yp136 was reported [16,17] to share similar chromosomal locations in lake charr and brook charr, and given that Yp136 maps to AC-18 [15], it was postulated that BC-18 may house the sex determining region in brook charr. Similarly, markers tightly linked to SEX in Arctic charr (i.e., Ots500NWFSC and SSOSL32)[18], were genotyped in the progeny raised to maturity in the HL3 and HL7 families.

The sex determining region in brook charr is localized to the BC-4 linkage group (Table 4), but was only found to be associated with segregation at one of the two markers we genotyped, that were previously reported to be tightly linked to SEX in Arctic charr (i.e., Ots500NWFSC)[18]. SSOSL32 was observed to be unlinked to SEX in the HL7 male parent.

**Table 4:** Associations between SEX and marker variation across various markers located within the two most likely sex linkage group regions in brook charr (i.e., either BC-18 or BC-4)

Linkage Group	Family	Marker	male alleles	No. of female progeny	No. of male progeny	χ^2 ^value (1 df); P-value from 1000 bootstrapping replicates
BC-18	HL3	BX079862	1	17	8	0.896; P = 0.369
			2	12	10	
BC-18	HL7	BX079862	1	14	11	2.768; P = 0.093
			2	7	15	
BC-18	HL3	BX319197	1	12	10	0.708; P = 0.408
			3	16	8	
BC-18	HL7	BX319197	2	8	18	3.305; P = 0.070
			3	12	9	
BC-4a	HL3	Ots500NWFSC	1	0	16	34.461; P = 0
			9	24	2	
BC-4a	HL7	Ots500NWFSC	1	0	26	42.081; P = 0
			9	19	1	
BC-4a	HL3	TC126859/i	1	26	2	31.056; P = 0
			2	1	14	
BC-4a	HL7	TC126859/i	1	0	28	48.000; P = 0
			2	20	0	
BC-4b	HL7	SSOSL32	2	8	17	2.006; P = 0.182
			3	12	11	
BC-9^1^	HL3	TC126859/ii	1	15	10	0.065; P = 0.824
			4	14	8	
BC-9	HL7	TC126859/ii	1	9	15	0.174; P = 0.705
			3	10	13	

^1 ^Sex linkage was tested on BC-9 due to the fact that marker TC126859 is duplicated in brook charr.

## Discussion

Comparative linkage group arrangements in the more completely characterized genetic map of Arctic charr, along with comparative analyses of the linkage group and chromosome arm arrangements in rainbow trout have provided a more detailed and comprehensive understanding of the genetic map arrangements in brook charr outlined in this study. These comparative analyses indicate that there are large regions of linkage group arm retention in the genetic maps of brook charr, Arctic charr, and rainbow trout. Thirty-seven of the expected 42 linkage groups in brook charr were tentatively identified in this study. In addition, one of the singleton markers genotyped in the survey may correspond to a separate linkage group arm in rainbow trout (i.e., RT-19p), suggesting affinities to 38 possible linkage groups.

The karyotype of brook charr purportedly has 34 acrocentric and 8 metacentric chromosomes [8]. Although the map depicted here has been assessed as having at least 8 metacentrics, up to 11 metacentrics may occur in the species, if indeed additional marker genotyping establishes that BC-15, BC-17, and BC-25 are also metacentric in structure. As evidenced from the mapping associations, it appears that two linkage groups (i.e., BC-4 and BC-16) may exhibit polymorphisms wherein some individuals may possess two acrocentrics associated with these linkage groups while others exhibit a metacentric structure. Furthermore, without a FISH analysis of the physical map, we cannot fully designate the linkage groups depicted in Table 1 as being metacentric in structure versus some type of whole-arm fusion event, similar to the karyotypic arrangements in Atlantic salmon [13]. These linkage groups may also be representative of some partial arm translocation event. However, intraspecific chromosome rearrangements are not that uncommon in salmonids, and the alignment of distinct rainbow trout chromosome arms to the various *Salvelinus *linkage groups suggests that an interpretation of whole-arm rearrangements is more likely.

### Recombination rate

It is likely that the recombination rate differences identified in this study are not representative of genome-wide differences in recombination rate given the small number of comparisons used to produce these estimates. Within brook charr, comparisons were limited to 9-17 marker intervals (14-27 pairwise comparisons) and thus much of the genome was not represented in the various estimates. When multiple intervals within a single linkage group were present for comparison, recombination ratios between the parents being compared were often variable (e.g., BC-16), indicating the importance of complete genome coverage for accurate average recombination rate estimates. However, BC-16 is homologous to RT-8 in rainbow trout, and RT-8 has been reported to have extremely unusual recombination rate dynamics, in that both female and male recombination rates were observed to be greatly suppressed throughout most of the length of the linkage group [11]. Thus, intrinsic factors regulating crossing-over mechanics may be much more variable within this particular genomic region in salmonids.

In the one case where recombination rate was significantly higher in the male mapping parent relative to the female, the loci (OMM5102/ii and BHMS465/i on BC-24) appear to be located near the telomere. Comparative mapping places OMM5102 distally (i.e., towards the telomeres) on the homologous Arctic charr (AC-3) and rainbow trout (RT-7q) linkage groups. BHMS465 has not been mapped in rainbow trout and the one copy mapped in Arctic charr maps distally on AC-24. Assuming that this pair of loci is located telomerically on BC-24, these results are not surprising in light of the work of Sakamoto et al. [19], who found recombination rates to be elevated in males relative to females in putative telomeric regions of the linkage group arms. Multivalent formations during Meiosis I restrict crossing over events to the telomeric regions of many chromosomes in males, thus resulting in suppressed recombination in regions proximal to the centromere and increased recombination in regions closer to the telomere [11,19,20]. In salmonids, these formations appear to be restricted to males (see [11] for an exception), hence the higher recombination rates observed in males relative to females in the telomeric regions of some chromosomes. Lastly, it should be noted that the higher male versus female recombination rate detected on BC-24 was not significant following Bonferroni correction.

The pairwise female: male recombination rates observed in this study among all four possible pairwise combinations of the mapping parents in brook charr (i.e., 2.41: 1) is somewhat higher than the levels observed in Arctic charr (i.e., 1.60: 1 - updated data based upon 550 map interval comparisons among the 4 mapping parents). This level of recombination is more similar to what has been observed in the rainbow trout mapping panels (i.e., ~ 2.95:1) [10], and much lower than levels observed in Atlantic salmon (i.e., ~ 7.23:1 - 8.26:1)[10,21,22]. Too few interspecific homologies existed within brook charr to permit even a preliminary analysis of whether these differences are in fact significant. A future reanalysis should permit a better understanding of this phenomenon with respect to the establishment of whether species with more acrocentric-based karyotypes do in fact have lower overall sex-specific recombination rates compared to those with more metacentric-based karyotypes, as suggested by Qumsiyeh [23].

### Segregation distortion

Evidence exists that segregation distortion can influence marker order, estimates of map distances, and linkage relationships [24]. While theoretical work by Hackett and Broadfoot [25] suggests segregation distortion at a single locus on a linkage group should have little effect on recombination estimates, the presence of two loci showing segregation distortion can result in the detection of false-positive linkage between two or more linkage groups [24]. However, these models are based upon tests of zygotic segregation distortion resulting from tests of combined parental genotypic combinations, such as those implemented when trying to build consensus genetic maps. Tests of gametic segregation distortion (conducted in this study) for assessing sex-specific genetic maps are expected to have less pronounced effects on linkage map construction and are a more accurate method of assessing such differences [26]. Even gametic phase distortion may, however, be associated with increased estimates of recombination distances between linked markers [15]. Localization of markers around recombination 'hot-spots' may also lead to a disruption in marker orders, but this effect may only be pronounced in regions of the salmonid genome involved with quadrivalent formations during meiosis (e.g., male meioses) [19].

All mapping parents except the LN4F contained markers on BC-16 which exhibited significant segregation distortion prior to Bonferroni correction. This might have partially accounted for the variability in map distances observed among mapping parents. Interestingly, the homologous linkage group to BC-16 in rainbow trout (i.e., RT-8) has a large degree of recombination suppression in females [11,12]. This region is also of interest evolutionarily as RT-8 appears to contain one or more genes important for several life-history traits, including development rate [27,28], spawning time [29 - 31] and maturation timing [32]. It has been argued that reduced recombination can be adaptive in that it helps to preserve highly compatible combinations of genes or gene complexes [33]. Whether the high degree of segregation distortion observed for markers on BC-16 stem directly from the importance of the genes within this linkage group region is unclear (i.e., are slight genomic incompatibilities more pronounced within BC-16 due to disruption of co-adapted gene complexes?). The mapping of additional markers to BC-16 within these experimental mapping panels and indeed additional brook charr families would assist in understanding the recombination 'hot-spot' dynamics within this chromosomal region.

Lastly, it should be noted that segregation distortion rates might be elevated in the HL brook charr due to their hybrid history. In Arctic charr, segregation distortion is elevated in hybrid relative to pure strain families [15]. Given the history of hybridization in the HL strain brook charr [34], the frequency of segregation distortion in HL brook charr might be elevated relative to that of pure strain brook charr. With so few markers currently mapped in LN brook charr, even comparing relative frequencies of segregation distortion between HL and LN brook charr is not particularly informative and thus this relationship cannot be tested at present. Therefore, it is important to recognize that segregation distortion rates observed in Hill's Lake brook charr might be an overestimation of typical rates for pure strain brook charr.

### Sex linkage

The observation that SEX is unlinked to the SSOSL32 marker in brook charr but appears tightly coupled to the Ots500NWFSC marker on BC-4 is intriguing given the polymorphisms with SEX linkage reported in Arctic charr [18]. In Arctic charr, SSOSL32 and Ots500NWFSC variation has been consistently linked to SEX, while other markers on AC-4 have shown variable associations. This could be due to the fact that this linkage group may be split into two acrocentric arms in certain individual males and yet be retained as a metacentric chromosome in other males, or result from pseudolinkage [18]. A similar polymorphism appears to exist in brook charr. Given that SSOSL32 and Ots500NWFSC appear to map close to one another in the central portion of the AC-4 linkage group [17], it is possible that some type of inversion has occurred in the homologous region of BC-4 to uncouple this association. This may have resulted in the placement of SSOSL32 onto the linkage group arm that shows variable associations with SEX in *Salvelinus*. Clearly, the examination of this sex linkage association in additional families of brook charr is needed. Ideally, this should be conducted across multiple strains of the species. In addition, a more complete genotyping survey of the markers located on this linkage group is needed in order to assess more precisely the 'break-points' in the SEX: marker associations, and define more accurately the recombination distances between SSOSL32 and Ots500NWFSC in brook charr.

### Homeologous and homologous affinities

Given that the modal number of chromosome arms in salmonids is 100 [13] and most Actinopterygiians have diploid chromosome numbers of 48 or 50 [35,36], it is expected that up to 25 homeologous affinities would be present in salmonids. As brook charr still possess the expected number of doubled chromosome arms following polyploidization, it was expected that any one brook charr linkage group would show homeology to only one other brook charr linkage group if the linkage group was representative of an acrocentric chromosome, or at most two other brook charr linkage groups if representative of a metacentric chromosome. Although greater than 25 putative homeologous affinities have been detected in the current study the expectation of 1:1 arm homeologies were largely met suggesting that there is a propensity to largely maintain evolutionary linkage arm arrangements in the salmonids. No homeologies were observed for five linkage groups (i.e., AC/BC-31, -33, -36, -39, and -43), in the combined *Salvelinus *linkage maps. Markers from AC/BC-31 are only syntenic with those on RT-16q (see Additional File 8, 9) confirming the status of this linkage group. For the other 4 linkage groups, it is possible that the designated linkage groups are only part of a larger linkage group that has not yet been identified (i.e., lack of intercalary markers genotyped to join the separated clusters), given that markers on each of these linkage groups assign to 2 - 4 different rainbow trout linkage group arms. However, for markers on AC/BC-36, -39, and -43, two or more markers define their assignments to rainbow trout linkage group arms RT-5p, -19q, and -9p. In each instance, these are the major cross homologies to the rainbow trout map suggesting that these are valid single linkage group arms. For AC/BC-33, assignments are possible to RT-11 (based upon a single marker homology), but this acrocentric linkage group also shares homology to AC/BC-4, -22, and therefore, further research is required to define this relationship. Since most assignments of cross homology for RT-11 are to AC/BC-22, it is possible that AC/BC-33 represents an unlinked fragment of AC/BC-22.

Linkage groups AC-15/BC-15 would appear to be a metacentric linkage group in structure, and along with AC-1/BC-1 and AC-3/BC-3, are three metacentric linkage groups that appear to have maintained a conserved structure between *Salvelinus *and *Oncorhynchus*. AC/BC-1, -3, and -15, are homologous to metacentric groups RT-29, -15, and -10, respectively, in rainbow trout. Only 1 arm of AC/BC-15 has been identified as possessing a homeologous affinity to AC/BC-10, and this pair of arms corresponds to the RT-10/18 homeology grouping [10]. The arm from AC-15/BC-15 lacking homeologous affinities to AC-10/BC-10, is homologous to RT-10p arm (see Additional File 9), supporting the contention that AC-15/BC-15 represent metacentric chromosomes.

Four of the duplicated genetic markers genotyped had only a single copy assigned to a known linkage group, while the other duplicate is currently recorded as a singleton. Unlinked copies of BX073647 and CA368462 appear to be homologous to rainbow trout linkage group arms where there is no coverage by the current genetic map for brook charr. BX073647 maps to homeologous linkage groups RT-17p/22p, and CA368462 maps to homeologous linkage groups AS-17/33, which is also homologous to the RT-17p/22p linkage group pair in rainbow trout [10]. Cross homology assignments would suggest that BX073647 and CA368462 are located on AC/BC-8/18 linkage groups, which needs to be confirmed with additional marker genotyping.

Four putative homeologies (BC-6/35, BC-14/30, BC-16/37 and BC-27/35) identified in brook charr, have not been detected to date in Arctic charr. While most of the identified homeologies in brook charr appear to represent conserved, known homeologous affinities in Arctic charr, rainbow trout, and Atlantic salmon, two of these (i.e., BC-14/30 and BC-16/37) appear to represent relationships which are not currently identified in other salmonine species. BC-14 shows homology to RT-19p (single marker) and RT-24q (multiple markers), while BC-30 shares homology to RT-3p and RT-6p (each with single marker affinities). Regions on RT-6p, and 19p are derived from the M ancestral karyotypic lineage in teleost fishes [10], suggesting that this homeology may be representative of either a 3R or 4R WGD homeology. BC-16 shares homology to RT-8 while BC-37 is related to RT-21p. There is a small segment on RT-21p derived from the F ancestral lineage of fishes, while it appears that most of the RT-8q arm is derived from the M ancestral lineage, and the RT-8p arm from the I lineage. Current data therefore, do not reconcile an origin for this homeology (i.e., BC-16/37) from the known duplicated segments in salmonids. With respect to the BC-6/35 and BC-27/35 homeologies, the former grouping shares affinity to the RT-27p/31p duplications (= B ancestral lineages), while the BC-27/35 region may relate to RT-6p/27q duplications (= possible K ancestral lineages), although it should be mentioned that the ancestral origins for the RT-6p arm are not well established [10], and therefore the assignment to other ancestral groupings may be revealed.

Interestingly, the majority of duplicated markers identified in brook charr correspond to linkage groups in rainbow trout and Atlantic salmon where the highest number of duplicated markers have been detected. Eight of the 12 conserved homologies between the duplicated homeologs in the two charr species and rainbow trout (Sal-23/35 and RT-27/31, Sal-3/24 and RT-7/15, Sal-12/27 and RT-12/16, Sal-1/21 and RT-2/29, BC-13b/34, AC-13/34 and RT-14/20, Sal-10/15 and RT-10/18, and BC-20a/20b and RT-2/9) are supported by a high number of duplicated markers in rainbow trout [10,13]. In addition, three of the six homeologies conserved between charr species and Atlantic salmon (BC-1/21 and AS-1/6, BC-13b/34 and AS-19/28, and BC-20a/20b and AS-4/11) are Atlantic salmon homeologies currently supported by the highest number of duplicated markers [13]. Phillips et al. [13] also observed a high degree of correlation in the number of duplicated markers supporting homeologous associations in both rainbow trout and Atlantic salmon. The observation that several of these markers remain duplicated in brook charr provides additional evidence for the continued exchange of information between these homeologous linkage groups across the Salmoninae. It remains unclear, however, why these chromosomal regions in particular contain such a high frequency of conserved duplicated markers.

The regions possessing the highest retention of duplicated markers are also those regions most likely to exhibit pseudolinkage in these species. These regions were homologous to RT-2p/29q; RT-2q/9q; RT-7q/15p; RT-12p/16p; RT-27p/31p in rainbow trout [10]. Here we report the expression of a pseudolinkage region on BC-1/21 (= RT-2p/29q homology). Apparent pseudolinkage arrangements have also been detected in the male mapping parents in two Arctic charr mapping panels involving three different linkage group regions (i.e., AC-12/27 (= RT-12p/16p); AC-6/23 (= RT-27p/31p); and AC-4/25a (= RT-11/12q/26)) [15; current study]. Hence, there appears to be a high degree of retention in the propensity to form quadrivalent pseudolinkage arrangements within a specific subset of the salmonid genome.

The suppression of diploidization in these linkage groups due to the continued exchange of chromosomal segments (especially in the telomeric regions of male quadrivalent formations) would ensure the continued retention of duplicated marker expression in populations exhibiting such phenomena. These meiotic processes would also shelter genetic markers towards the central parts of male metacentric linkage groups from recombination during meiosis [19]. Thus, it is tempting to speculate that one of the evolutionary forces driving these unusual meiotic processes in male salmonids is selection for co-adapted gene complexes. Whether synteny blocks prove to be less re-arranged within the centromeric regions of linkage groups exhibiting pseudolinkage regions awaits the completion of greater genomic sequence data.

## Conclusions

Examination of genetic markers in brook charr, Arctic charr, and rainbow trout linkage group arms suggests that a high degree of retention in marker affinities exist among salmonid chromosome arms. Evidence was obtained for potential polymorhisms in chromosome structure suggesting that two brook charr metacentric chromsosomes (i.e., BC-4 and BC-16) may also exist as two separate acrocentrics within individuals. BC-4 is the sex linkage group in brook charr and is homologous to the Arctic charr sex linkage group AC-4. Possible sex chromosome polymorphisms (i.e., fusions and fissions and/or pseudolinkage) have also been detected with the Arctic charr AC-4 linkage group. Brook charr linkage groups possessing more duplicated markers appear more likely to exhibit pseudolinkage, and in general, pseudolinkage regions appear to be conserved among salmonid species.

## Methods

### Arctic charr mapping panels

Microsatellite markers were named as outlined in Sakamoto et al. [19]. Microsatellite markers which appeared to amplify multiple copies, only one of which was polymorphic, were not designated as being duplicated. Multiple copies amplified by a single primer set are identified by "/i", "/ii" or "/iii". An updated genetic map for two Arctic charr mapping families (Family 2 & 3) [15] was used as template in this study for comparisons of arm homologies to brook charr, and was also used as a reference *Salvelinus*-based map to make comparative synteny assignments to the more complete linkage map available for rainbow trout [10]. The reference Arctic charr map used in the study had 620 markers assigned to it across 37 different linkage groups, while the rainbow trout map used for comparative purposes had 2055 markers assigned across 29 linkage groups.

### Brook charr mapping panels

The mapping panels consisted of two families (HL3 and HL7) from the Hill's Lake (HL) strain and one from the Lake Nipigon (LN) strain (LN4). The HL strain has been maintained for 20 generations at the Hill's Lake Fish Culture Station near Englehart, Ontario, and for an unknown number of generations in Pennsylvania prior to transfer to the Hill's Lake Fish Culture Station [37]. This strain has been crossed with a wild strain of brook charr on at least one occasion in the 1960s [34; OMNR Fish Culture Section Catalog Update 2005]. Lake Nipigon brood stock from the Hill's Lake Fish Culture Station were derived from wild collections of gametes from Lake Nipigon in 1977 (14 females and 16 males), 1978 (24 females and 20 males), and 1979 (24 females and 30 males) [OMNR Fish Culture Section Catalog Update 2005].

HL gametes were collected from the Hill's Lake Fish Culture Facility and taken to the Codrington Fisheries Research Facility where families were made on October 4^th^, 2007. The LN family was made on December 13, 2007 at the Codrington Fisheries Research Facility from gametes collected at this facility. Adipose fin tissue was taken from all parents and stored at -80°C for later DNA extraction. The families were raised in 16 compartment FAL (Heath-style) vertical flow incubating racks fed by water chilled to a constant 4°C. When the embryos had visible eye pigment, the families were reduced to 120 individuals by randomly selecting embryos from each family. Fish used for the genetic map construction, were killed at 1-3 months post hatching and stored at -80°C. Final family progeny numbers were 115 for HL3, 113 for HL7, and 110 for LN4.

DNA was extracted from parental adipose fins and whole free-embryos (minus their yolk sacs) using a phenol - chlorophorm - isoamyl alcohol protocol [38]. Parents were screened in total for variation at 218 microsatellite loci, and the results from the polymorphisms detected are summarized in Additional File 10. Marker selection was based on knowledge of linkage groups in other salmonid species, especially those of Arctic charr and rainbow trout, in order to facilitate a directed genotyping approach whereby at least 2 - 4 markers were genotyped that were assumed to be homologous to separate salmonid linkage group arms. Expressed sequence tag (EST) microsatellite markers, primarily derived for use with rainbow trout and Atlantic salmon, were used preferentially over other molecular markers for the reasons outlined in Rise et al. [39] and Vasemägi et al. [40]. However, the goal of obtaining coverage of at least two markers on each Arctic charr linkage group required several microsatellite markers developed from non-coding DNA to be used. In addition, 10 primer pairs designed to amplify copies of the *Clock *gene were screened for variation in the mapping panels. Of the markers which had detectable polymorphism in the parents, 101 microsatellites and two copies of *Clock *were selected to be used for the construction of the linkage map. All progeny from HL3, HL7 and LN4 were genotyped for the subset of 103 polymorphic markers for which they were informative. Counting all the duplicated locus positions that were amplified with some of the polymorphic markers, the final comparative maps generated from the three brook charr mapping panels consisted of 114 (HL3), 116 (HL7), and 54 (LN4) informative loci in each family (see Additional File 1).

To assess the putative location of the sex determining region in brook charr, additional progeny from the HL3 and HL7 mapping families were reared at the Codrington Fisheries Research Facility for an additional 2.5 years, when 48 fish from each of these families were sacrificed and internally sexed in September, 2010. DNA was then extracted from fin clips on these fish and genotyped for markers on the BC-18 (= homology to Yp136 marker region), and BC-4a; BC-4b linkage group regions (= homology to the AC-4 sex linkage group).

### Linkage map construction

Linkage analysis was performed using a number of programs (LINKMFEX, LINKGRP, GENOVECT-batch) contained in the LINKMFEX software suite that is available in the Link to Computer Software tab at (http://www.uoguelph.ca/~rdanzman/) [26], and unless otherwise cited, all program modules mentioned can be found at this website. Due to the complex nature of gene transmission in salmonids, linkage maps were constructed separately for each sex. Maps were constructed using a minimum LOD threshold of 3.0 to assign linkage between loci, and markers were clustered into their respective linkage groups with LINKGRP. Linkage group specific pairwise distance files were created using LINKMFEX. Marker orders within each linkage group were determined with MAPORD. Finally, marker distances within each linkage group were calculated with MAPDIS-V, which also created a map file formatted to produce a graphical linkage map in the program MAPCHART [41] (http://www.joinmap.nl). To facilitate future cross comparisons of linkage groups within fishes of the genus *Salvelinus*, all brook charr linkage groups were designated according the their homologous chromosome arms in Arctic charr following the designations given in [15].

### Recombination rates

Recombination rate differences along conserved chromosomal segments were calculated using the program RECOMDIF, which uses a two-way contingency G-test that compares parental versus recombinant genotypes inherited from each parent, for each pair of linked markers shared between the mapping parents being compared. Williams' correction was applied when the number of recombinants was less than five. Bonferroni correction for multiple comparisons was applied by dividing alpha (0.05) by the number of linkage groups tested in each comparison in order to determine the adjusted critical *χ*^2 ^values. For all comparisons, only recombination rates between adjacent marker intervals within linkage groups were compared. Duplicated marker designations were ignored for these comparisons (i.e., marker/i or marker/ii designations were considered equivalent as these assignments are arbitary, and may in fact cross-assign to the homeologous linkage groups across the various mapping parents).

### Segregation distortion

For all polymorphic loci (except those where both parents were heterozygous for the same alleles) goodness of fit to the expected 1:1 segregation ratio was assessed using a log likelihood adjusted *χ*^2 ^test (with the program LINKMFEX), which is an appropriate test for sample sizes between 25 and 200 [42]. The program SEGsort was used to compile a list of all markers showing significant deviation (p < 0.05), prior to Bonferroni correction, from the expected 1:1 segregation ratio. As multiple tests were performed for each mapping parent, Bonferroni correction for multiple comparisons was applied as described above.

### Comparative analysis

Linkage map comparisons between the brook charr and Arctic charr mapping parents ([15]; plus updated mapping data), rainbow trout ([10], plus updated mapping data), were made using Oxford grids. For comparative purposes, and to potentially help resolve ambiguous map assignments, reference of homologous marker locations was also made to the Atlantic salmon linkage map. However, fewer homologous markers are localized between Atlantic salmon and the two *Salvelinus *species, and therefore, more extensive comparisions are not reported in this study. The programs markerSORT and markerCOMP, were used to assist in the assignment of cross-species homologies and homeologies. Putative cross species homologies and homeologies were assigned based on conserved syntenic blocks of markers, and conserved syntenic blocks of duplicated markers (except where noted differently), respectfully. The construction of marker-specific synteny blocks was accomplished using the program BLOCKON with Arctic charr as the reference genome to depict assignments to brook charr and rainbow trout arm homologies. Using the composite female Arctic charr map, it was also possible to infer the more precise structure of the composite *Salvelinus *linkage groups by referencing each linkage group to the more complete rainbow trout linkage map. *Salvelinus *linkage groups showing homology to two different linkage group arms in rainbow trout were inferred to be metacentric in structure. As comparisons to a physical map have not been completed for either Arctic charr or brook charr at present, linkage group arms were arbitrarily designated as 'a' and 'b' arms within metacentrics.

## Authors' contributions

ERT performed the genotyping on the brook charr mapping panels while HKM and JDN contributed to adding new markers on the Arctic charr genetic map. ERT and RGD analyzed and interpreted the data as well as wrote the manuscript, while CCW organized the family crosses and rearing of the fish. MMF, CCW and RGD conceptualized the study, while all authors read and commented on the paper.

## Supplementary Material

Additional file 1 **Assignment of polymorphic markers to the various identified brook charr linkage groups in three mapping panels (HL3, HL7, and LN4)**. Click here for file

Additional file 2 **Composite female linkage maps derived from mapping data in the HL3 and HL7 mapping panel female parents**. Click here for file

Additional file 3 **Linkage map based upon genotypic segregation data from the HL3 and HL7 mapping panel male parents**. Click here for file

Additional file 4 **Assignment of polymorphic markers to the various identified Arctic charr linkage groups in two mapping panels (Family 2 and Family 3)**. Click here for file

Additional file 5 **Within family comparisons (Hills Lake strain) of recombination rate differences between female and male brook charr mapping parents**. Click here for file

Additional file 6 **Intra-sex comparisons of the recombination rates between the Hills Lake brook charr female and male mapping parents**. Click here for file

Additional file 7 **Observed deviations from Mendelian expectations in the brook charr mapping parents of families HL3 and HL7**. Click here for file

Additional file 8 **Comparative genetic maps between Arctic charr, brook charr, and rainbow trout, using the combined female of Arctic charr as a template**. Click here for file

Additional file 9 **Oxford Grid of the linkage group arm assignments between *Salvelinus *(based primarily upon the Arctic charr) and rainbow trout**. Click here for file

Additional file 10 **Complete listing of all the genetic markers screened in the brook charr mapping panels**. Click here for file
