# Supplementary material for: Genome evolution in the fish family salmonidae: generation of a brook charr genetic map and comparisons among charrs (Arctic charr and brook charr) with rainbow trout
Source: BMC Genet. 2011 Jul 28;12:68. doi: 10.1186/1471-2156-12-68 (PMC3162921; doi:10.1186/1471-2156-12-68)
Supplement: Additional file 2 — Composite female linkage maps derived from mapping data in the HL3 and HL7 mapping panel female parents. [file 1471-2156-12-68-S2.PDF]

Additional File 2: Composite female linkage map for brook charr. Maps are derived from the combined genetic maps of the HL3 and HL7 mapping panels. For certain linkage groups separate linkage groups are depicted due to large differences in recombination distances or lack of 2 or more shared markers required for marker ordering.

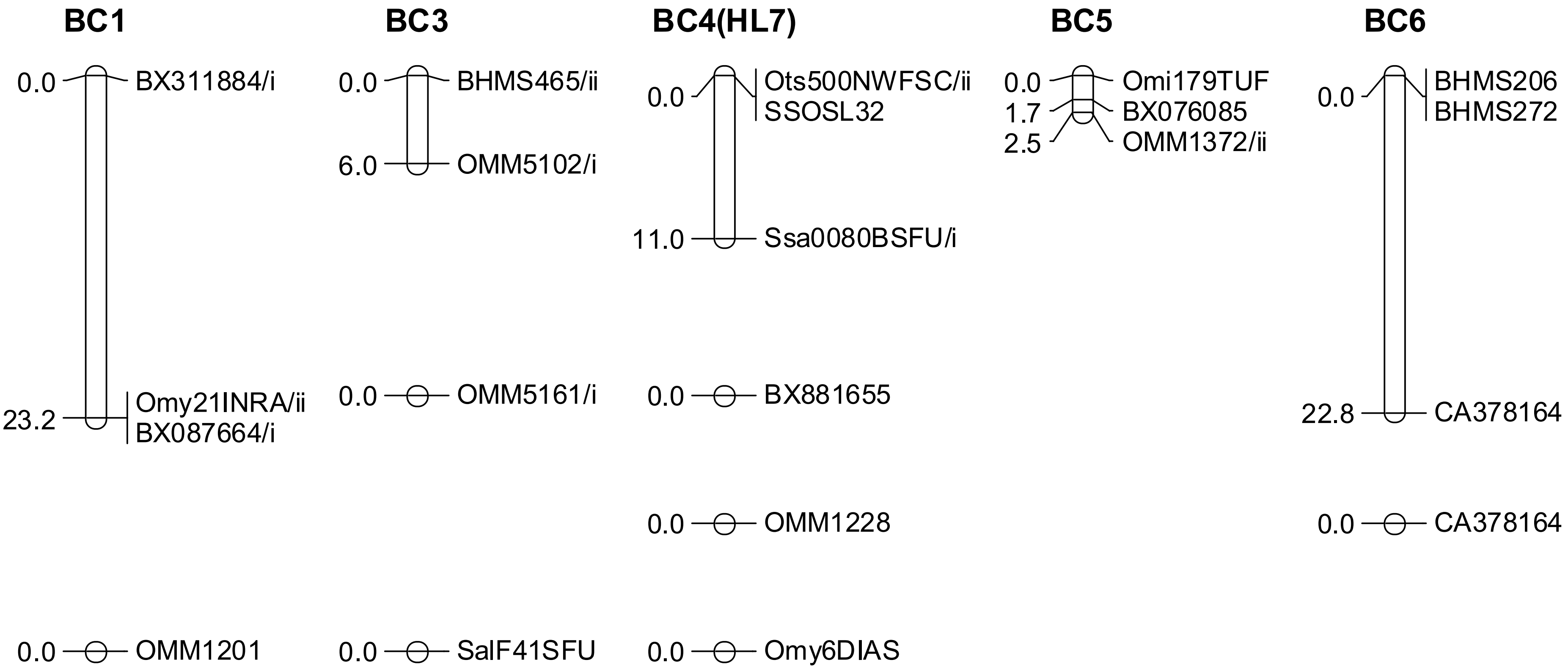
BC1
0.0 BX311884/i
23.2 Omy21INRA/ii
BX087664/i
0.0 OMM1201
BC3
0.0 BHMS465/ii
6.0 OMM5102/i
0.0 OMM5161/i
0.0 SalF41SFU
BC4(HL7)
0.0 Ots500NWFSC/ii
SSOSL32
11.0 Ssa0080BSFU/i
0.0 BX881655
0.0 OMM1228
0.0 Omy6DIAS
BC5
0.0 Omi179TUF
1.7 BX076085
2.5 OMM1372/ii
BC6
0.0 BHMS206
BHMS272
22.8 CA378164
0.0 CA378164

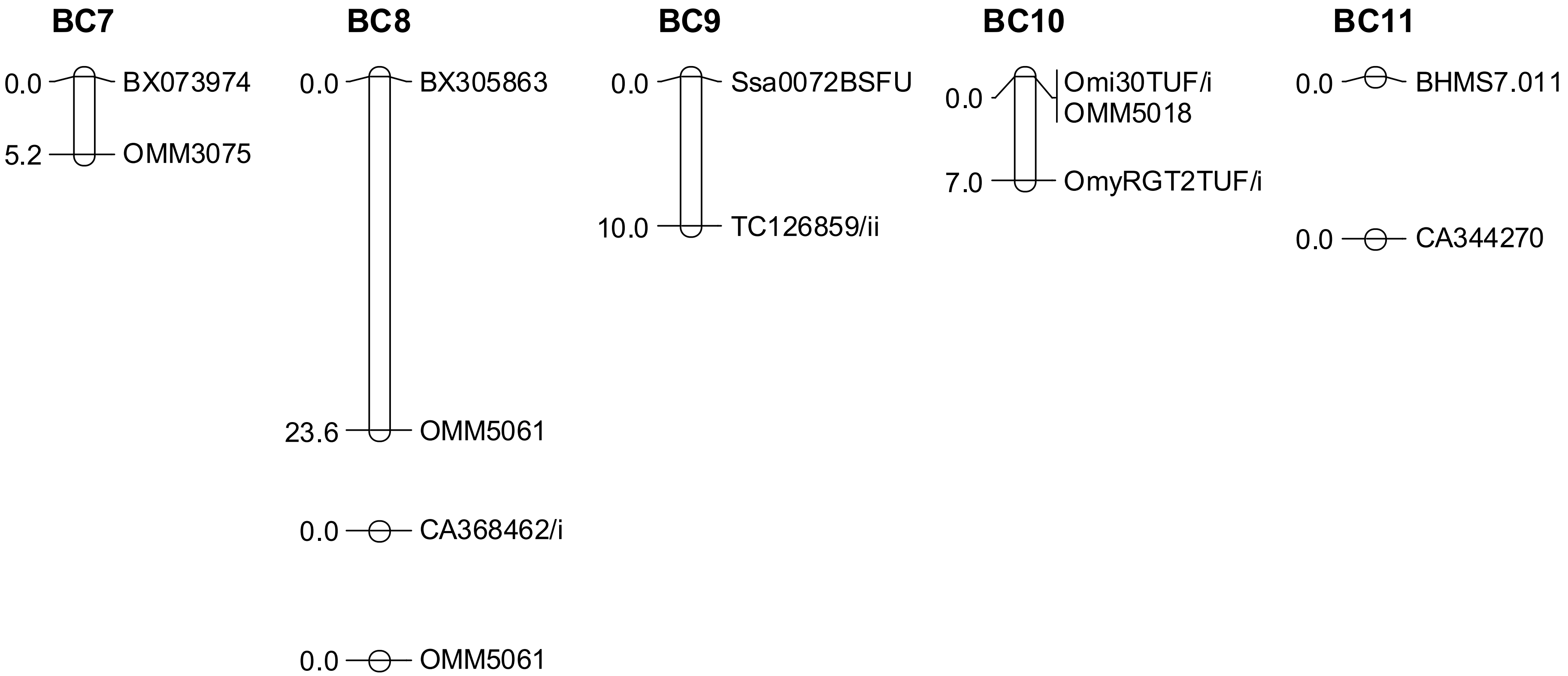
BC7
0.0 BX073974
5.2 OMM3075
BC8
0.0 BX305863
23.6 OMM5061
0.0 CA368462/i
0.0 OMM5061
BC9
0.0 Ssa0072BSFU
10.0 TC126859/ii
BC10
0.0 Omi30TUF/i
OMM5018
7.0 OmyRGT2TUF/i
BC11
0.0 BHMS7.011
0.0 CA344270

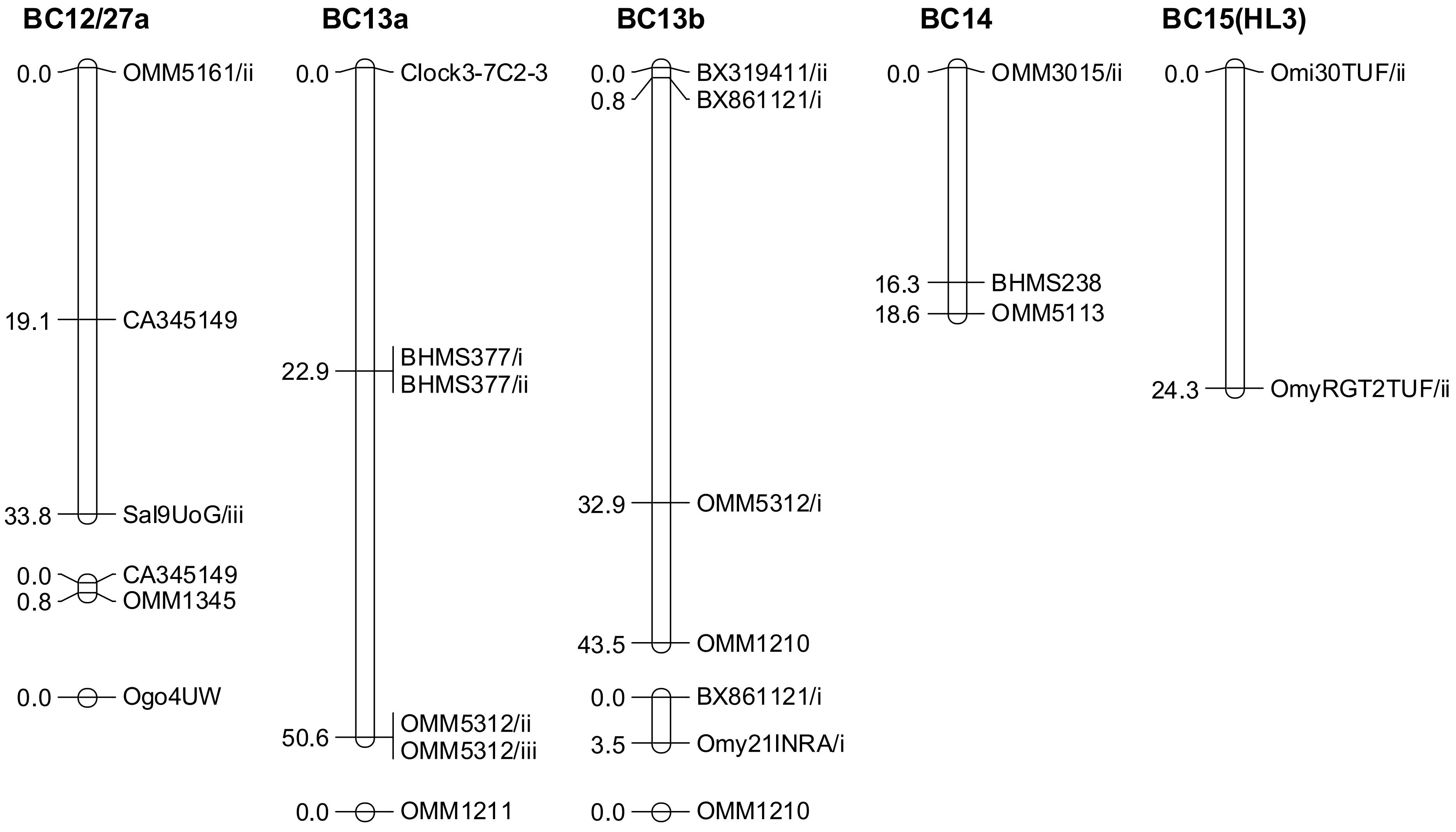
BC12/27a
0.0 OMM5161/ii
19.1 CA345149
33.8 Sal9UoG/iii
0.0 CA345149
0.8 OMM1345
0.0 Ogo4UW
BC13a
0.0 Clock3-7C2-3
22.9 BHMS377/i BHMS377/ii
50.6 OMM5312/ii OMM5312/iii
0.0 OMM1211
BC13b
0.0 BX319411/ii
0.8 BX861121/i
32.9 OMM5312/i
43.5 OMM1210
0.0 BX861121/i
3.5 Omy21INRA/i
0.0 OMM1210
BC14
0.0 OMM3015/ii
16.3 BHMS238
18.6 OMM5113
BC15(HL3)
0.0 Omi30TUF/ii
24.3 OmyRGT2TUF/ii

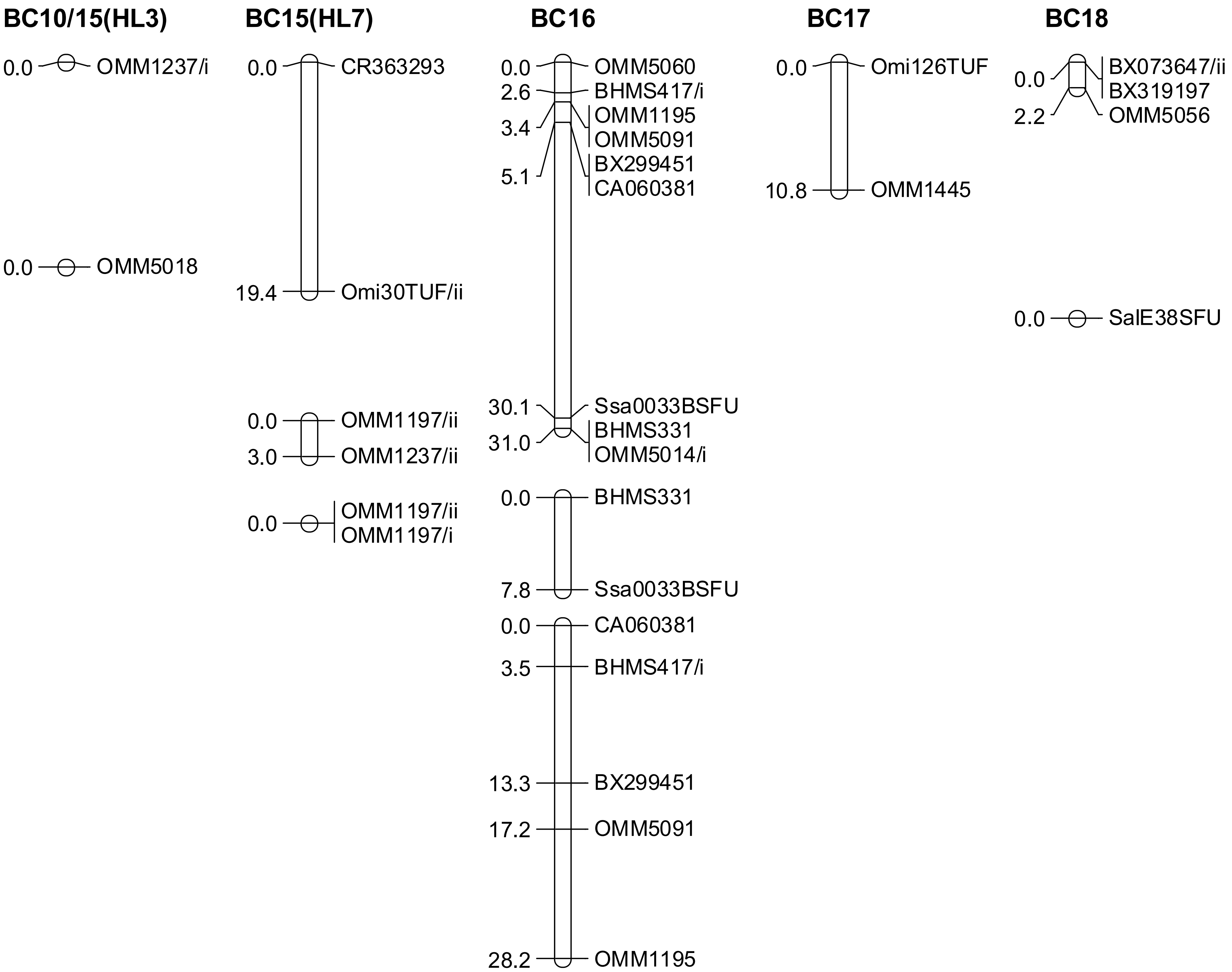

BC10/15(HL3)
0.0 OMM1237/i
0.0 OMM5018
BC15(HL7)
0.0 CR363293
19.4 Omi30TUF/ii
0.0 OMM1197/ii
3.0 OMM1237/ii
0.0 OMM1197/ii
OMM1197/i
BC16
0.0 OMM5060
2.6 BHMS417/i
3.4 OMM1195
OMM5091
5.1 BX299451
CA060381
30.1 Ssa0033BSFU
31.0 BHMS331
OMM5014/i
0.0 BHMS331
7.8 Ssa0033BSFU
0.0 CA060381
3.5 BHMS417/i
13.3 BX299451
17.2 OMM5091
28.2 OMM1195
BC17
0.0 Omi126TUF
10.8 OMM1445
BC18
0.0 BX073647/ii
BX319197
2.2 OMM5056
0.0 SalE38SFU

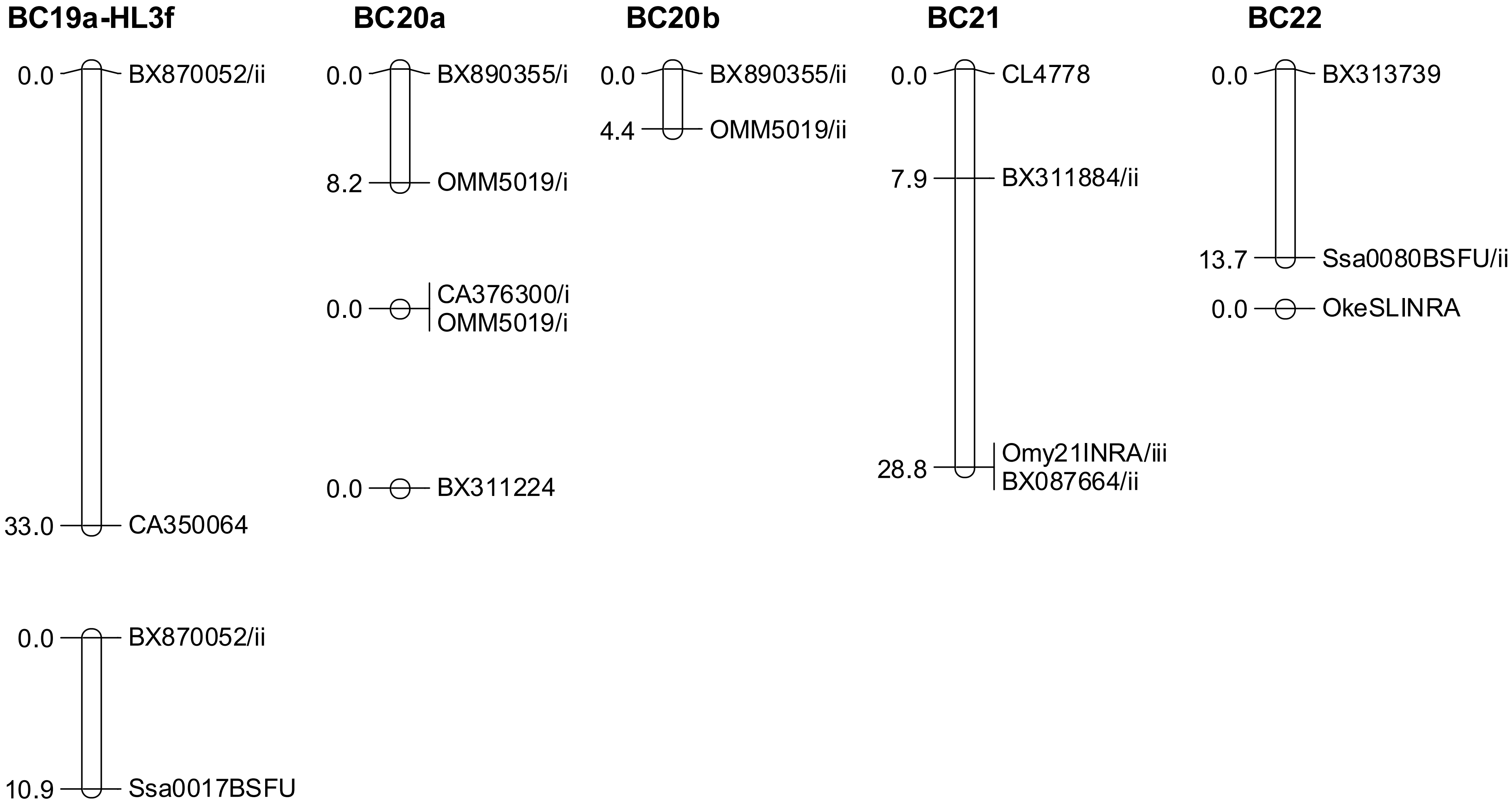
BC19a-HL3f
0.0 BX870052/ii
33.0 CA350064
0.0 BX870052/ii
10.9 Ssa0017BSFU
BC20a
0.0 BX890355/i
8.2 OMM5019/i
0.0 CA376300/i
OMM5019/i
0.0 BX311224
BC20b
0.0 BX890355/ii
4.4 OMM5019/ii
BC21
0.0 CL4778
7.9 BX311884/ii
28.8 Omy21INRA/iii
BX087664/ii
BC22
0.0 BX313739
13.7 Ssa0080BSFU/ii
0.0 OkeSLINRA

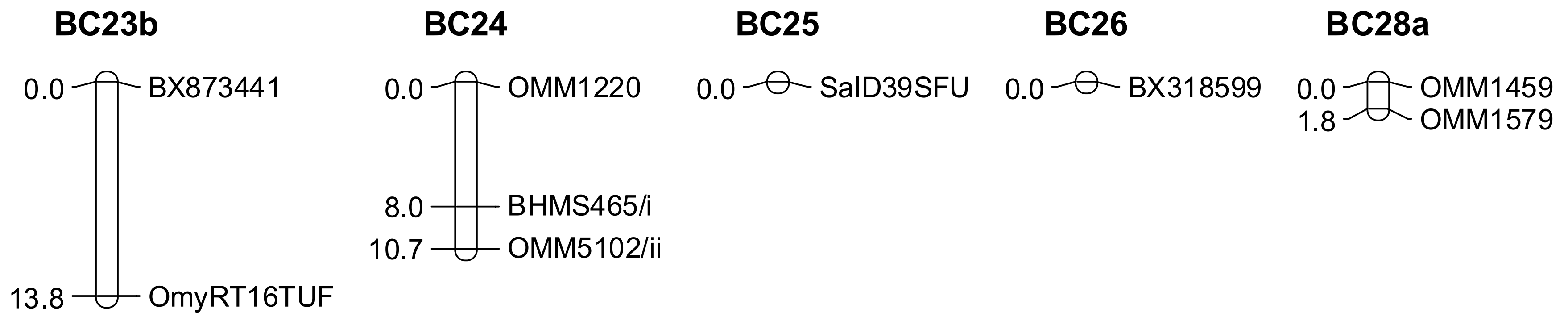
BC23b
0.0
BX873441
13.8
OmyRT16TUF
BC24
0.0
OMM1220
8.0
BHMS465/i
10.7
OMM5102/ii
BC25
0.0
SalD39SFU
BC26
0.0
BX318599
BC28a
0.0
OMM1459
1.8
OMM1579

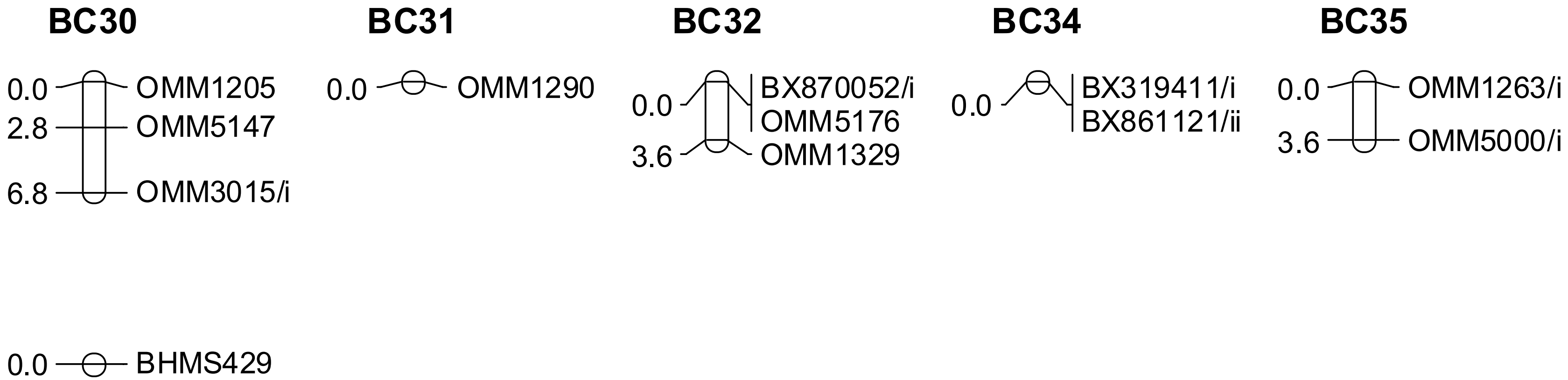

BC30
0.0 OMM1205
2.8 OMM5147
6.8 OMM3015/i
0.0 BHMS429
BC31
0.0 OMM1290
BC32
0.0 BX870052/i
OMM5176
3.6 OMM1329
BC34
0.0 BX319411/i
BX861121/ii
BC35
0.0 OMM1263/i
3.6 OMM5000/i

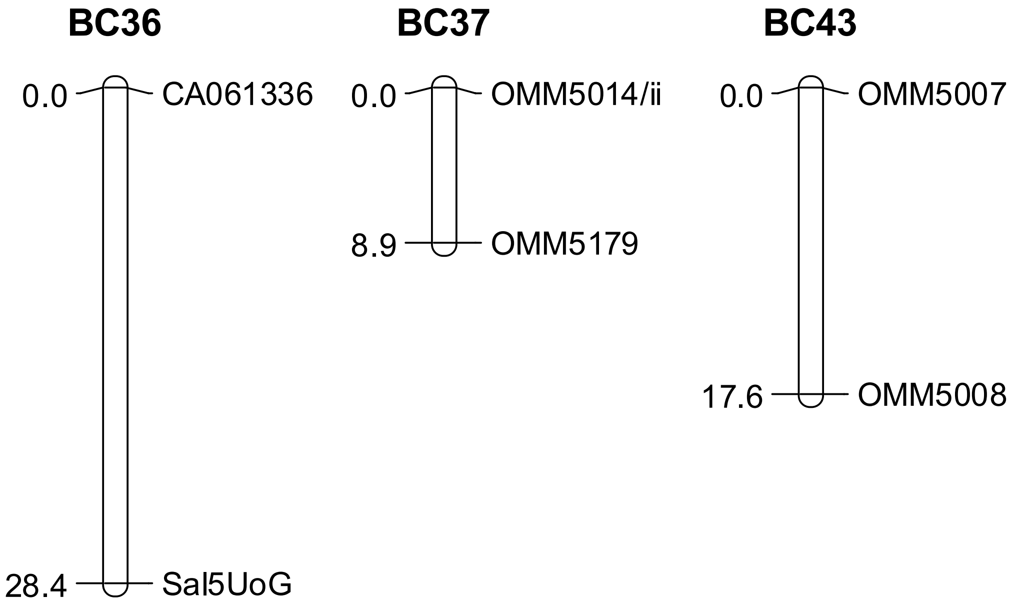
BC36
0.0
CA061336
28.4
Sal5UoG
BC37
0.0
OMM5014/ii
8.9
OMM5179
BC43
0.0
OMM5007
17.6
OMM5008
