# Supplementary material for: Genome evolution in the fish family salmonidae: generation of a brook charr genetic map and comparisons among charrs (Arctic charr and brook charr) with rainbow trout
Source: BMC Genet. 2011 Jul 28;12:68. doi: 10.1186/1471-2156-12-68 (PMC3162921; doi:10.1186/1471-2156-12-68)
Supplement: Additional file 3 — Linkage map based upon genotypic segregation data from the HL3 and HL7 mapping panel male parents. [file 1471-2156-12-68-S3.PDF]

Additional File 3: Composite male linkage map for brook charr. Maps are derived from the combined genetic maps of the HL3 and HL7 mapping panels. For certain linkage groups separate linkage groups are depicted due to lack of 2 or more shared markers required for marker ordering.

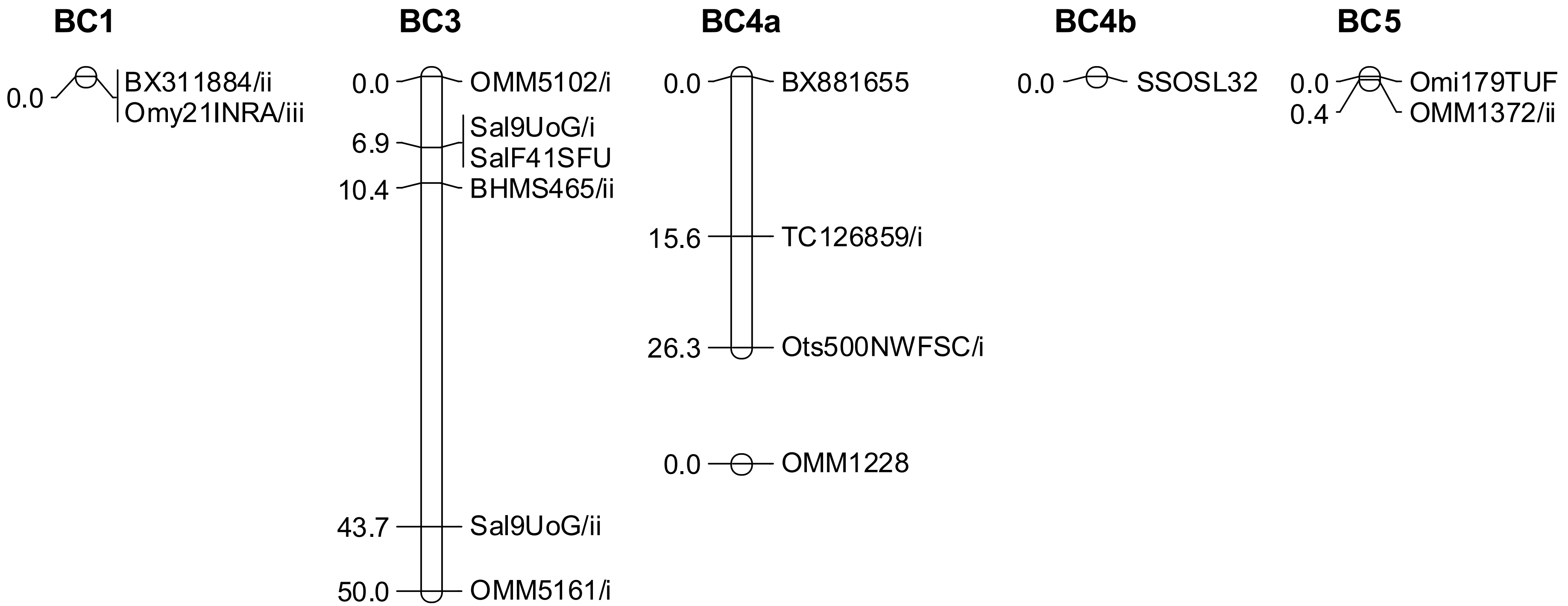
BC1
0.0
BX311884/ii
Omy21INRA/iii
BC3
0.0
OMM5102/i
6.9
Sal9UoG/i
SalF41SFU
10.4
BHMS465/ii
43.7
Sal9UoG/ii
50.0
OMM5161/i
BC4a
0.0
BX881655
15.6
TC126859/i
26.3
Ots500NWFSC/i
0.0
OMM1228
BC4b
0.0
SSOSL32
BC5
0.0
Omi179TUF
0.4
OMM1372/ii

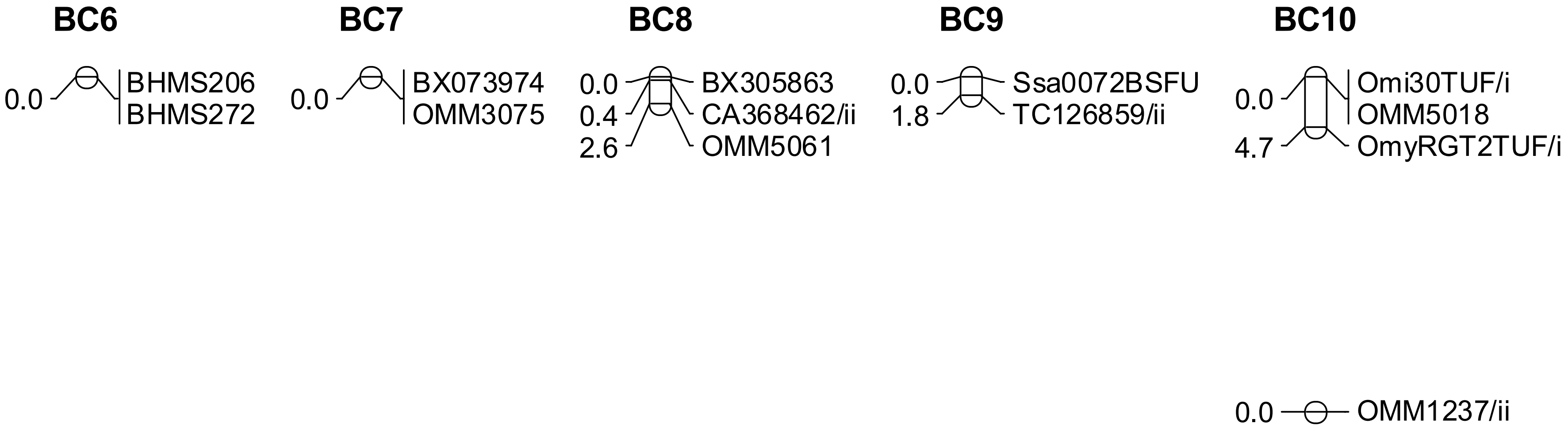
BC6
0.0
BHMS206
BHMS272
BC7
0.0
BX073974
OMM3075
BC8
0.0
0.4
2.6
BX305863
CA368462/ii
OMM5061
BC9
0.0
1.8
Ssa0072BSFU
TC126859/ii
BC10
0.0
4.7
Omi30TUF/i
OMM5018
OmyRGT2TUF/i
0.0
OMM1237/ii

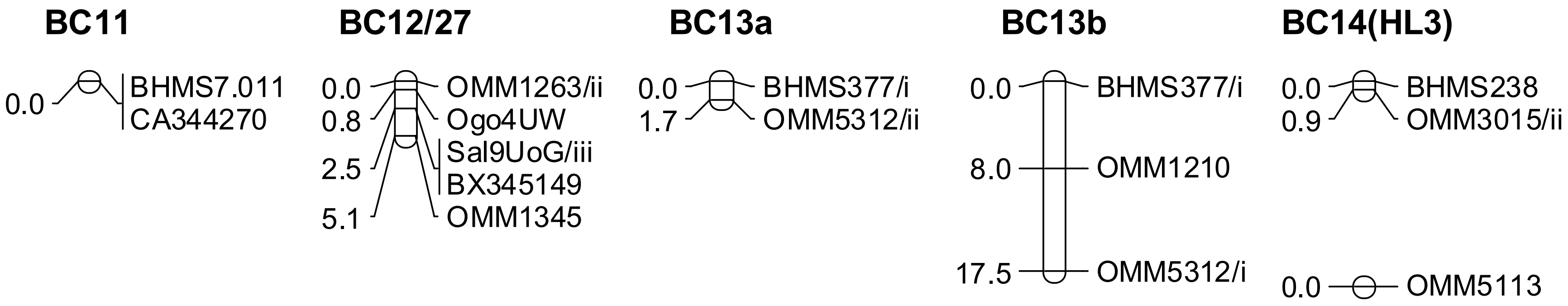
BC11
0.0
BHMS7.011
CA344270
BC12/27
0.0
0.8
2.5
5.1
OMM1263/ii
Ogo4UW
Sal9UoG/iii
BX345149
OMM1345
BC13a
0.0
1.7
BHMS377/i
OMM5312/ii
BC13b
0.0
8.0
17.5
BHMS377/i
OMM1210
OMM5312/i
BC14(HL3)
0.0
0.9
BHMS238
OMM3015/ii
0.0
OMM5113

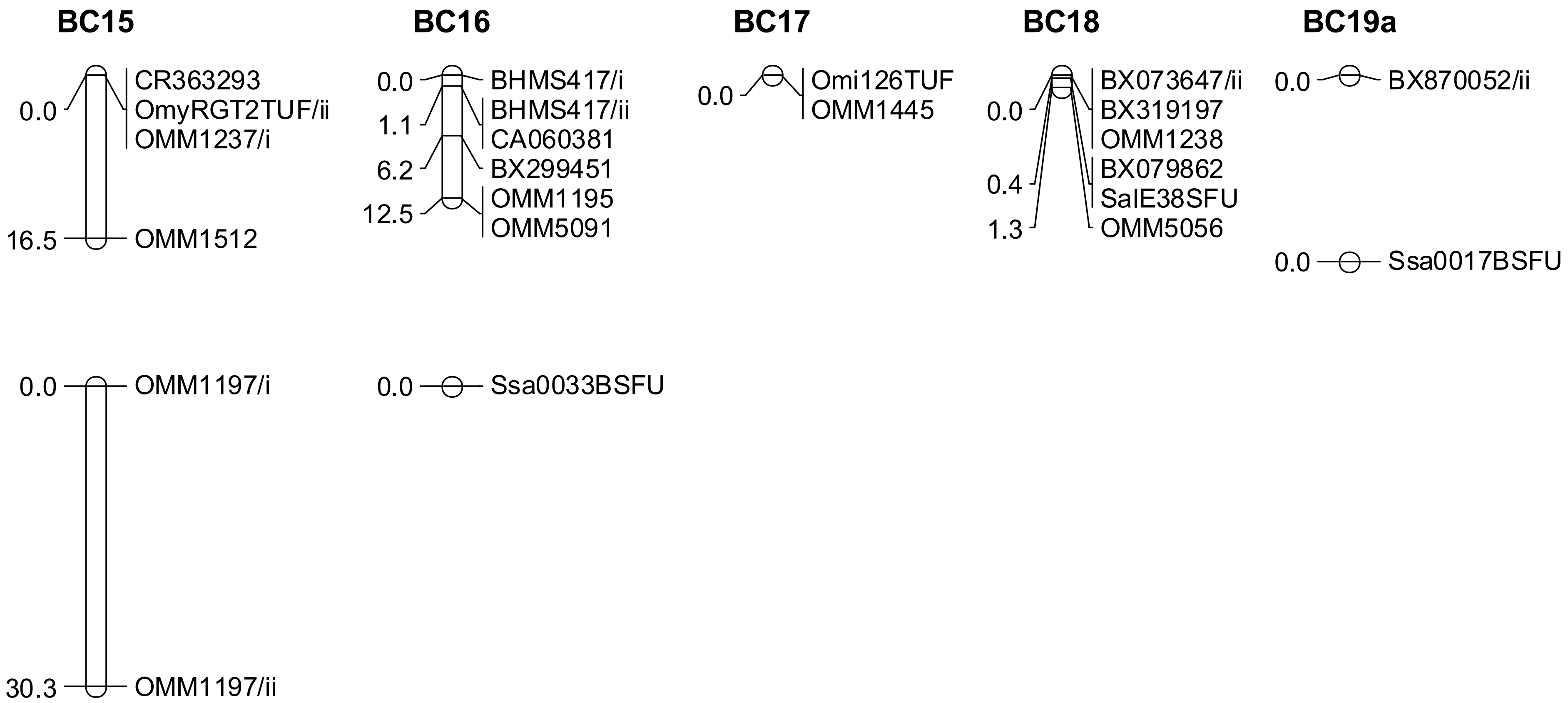
BC15
0.0
CR363293
OmyRGT2TUF/ii
OMM1237/i
16.5
OMM1512
0.0
OMM1197/i
30.3
OMM1197/ii
BC16
0.0
BHMS417/i
1.1
BHMS417/ii
CA060381
6.2
BX299451
12.5
OMM1195
OMM5091
0.0
Ssa0033BSFU
BC17
0.0
Omi126TUF
OMM1445
BC18
0.0
BX073647/ii
BX319197
OMM1238
0.4
BX079862
SaIE38SFU
1.3
OMM5056
BC19a
0.0
BX870052/ii
0.0
Ssa0017BSFU

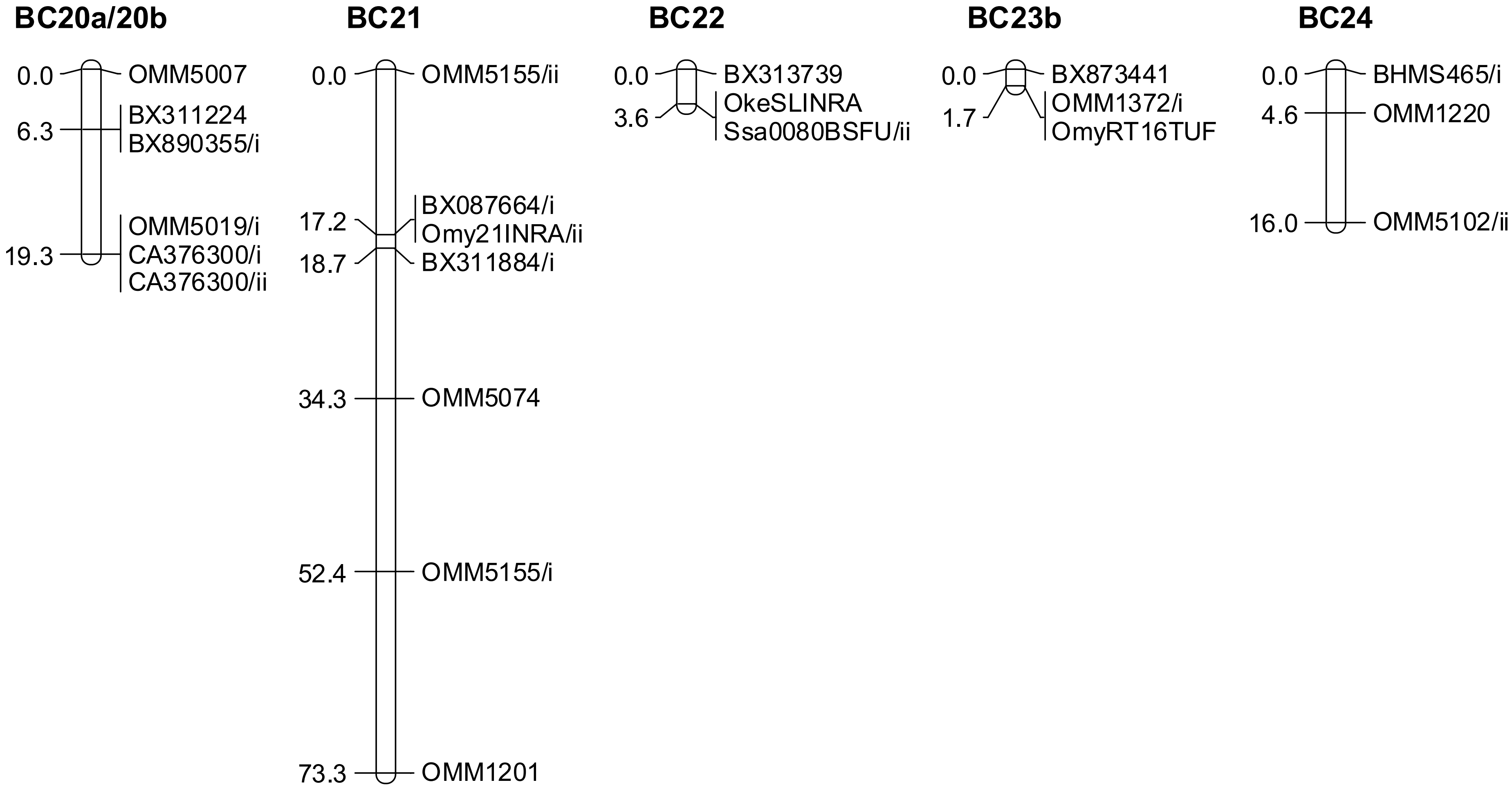
BC20a/20b
0.0
OMM5007
6.3
BX311224
BX890355/i
19.3
OMM5019/i
CA376300/i
CA376300/ii
BC21
0.0
OMM5155/ii
17.2
18.7
BX087664/i
Omy21INRA/ii
BX311884/i
34.3
OMM5074
52.4
OMM5155/i
73.3
OMM1201
BC22
0.0
BX313739
3.6
OkeSLINRA
Ssa0080BSFU/ii
BC23b
0.0
BX873441
1.7
OMM1372/i
OmyRT16TUF
BC24
0.0
BHMS465/i
4.6
OMM1220
16.0
OMM5102/ii

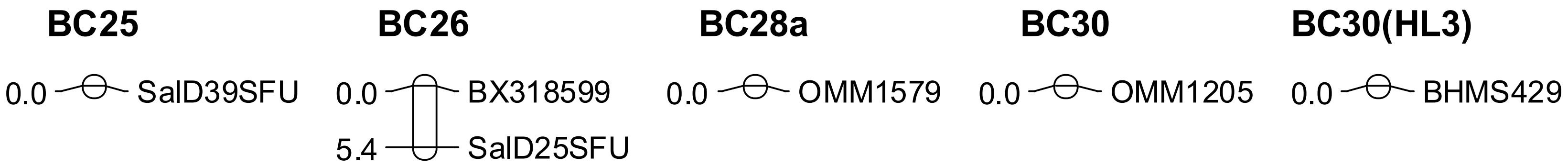
BC25
0.0
SalD39SFU
BC26
0.0
BX318599
5.4
SalD25SFU
BC28a
0.0
OMM1579
BC30
0.0
OMM1205
BC30(HL3)
0.0
BHMS429

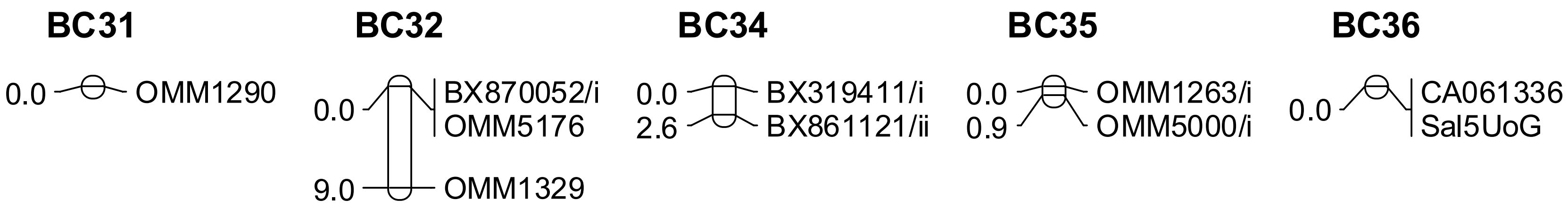
BC31
0.0
OMM1290
BC32
0.0
BX870052/i
OMM5176
9.0
OMM1329
BC34
0.0
BX319411/i
2.6
BX861121/ii
BC35
0.0
OMM1263/i
0.9
OMM5000/i
BC36
0.0
CA061336
Sal5UoG

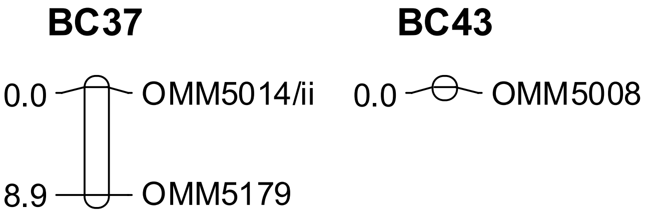
BC37
0.0
OMM5014/ii
8.9
OMM5179
BC43
0.0
OMM5008
