# Supplementary material for: Genome evolution in the fish family salmonidae: generation of a brook charr genetic map and comparisons among charrs (Arctic charr and brook charr) with rainbow trout
Source: BMC Genet. 2011 Jul 28;12:68. doi: 10.1186/1471-2156-12-68 (PMC3162921; doi:10.1186/1471-2156-12-68)
Supplement: Additional file 5 — Within family comparisons (Hills Lake strain) of recombination rate differences between female and male brook charr mapping parents. [file 1471-2156-12-68-S5.DOC]

Additional File 4 – PartA: Within family comparisons (Hills Lake strain) of recombination rate differences between female and male brook charr mapping parents within families HL3 and HL7.

| Family | LG | Marker 1 | Marker 2 | Ratio (F:M)^1^ | G-value^2^ | P-value^3^ |
| --- | --- | --- | --- | --- | --- | --- |
| HL3 | 5 | Omi179TUF | OMM1372/ii | 3.25:1 | 0.94 | NS |
| HL3 | 6 | BHMS206 | BHMS272 | 1 | 0 | NS |
| HL3 | 8 | BX305863 | OMM5061 | 8.74:1 | 23.24 | <0.001^3^ |
| HL3 | 9 | Ssa0072BSFU | TC126859/i | 2.78:1 | 3.51 | NS |
| HL3 | 10 | Omi30TUF/i | OMM5018 | 1 | 0.00 | NS |
| HL3 | 10 | Omi30TUF/i | OmyRGT2TUF/i | 1 | -0.01 | NS |
| HL3 | 10 | OMM5018 | OmyRGT2TUF/i | 9.45:1 | 29.28 | <0.001^3^ |
| HL3 | 13a | BHMS377/i | OMM5312/ii | 16.29:1 | 18.67 | <0.001^3^ |
| HL3 | 14 | OMM3015/ii | BHMS238 | 18.11:1 | 17.73 | <0.001^3^ |
| HL3 | 16 | CA060381 | BHMS417/i | 4.38:1 | 1.78 | NS |
| HL3 | 16 | BHMS417/i | BX299451 | 0.72:1 | 0.37^2^ | NS |
| HL3 | 16 | BX299451 | OMM1195 | 1 | 0^2^ | NS |
| HL3 | 23b | BX873441 | OmyRT16TUF | 6.76:1 | 9.26 | <0.005^3^ |
| HL3 | 18 | BX073647/ii | BX319197 | 1 | 0.00 | NS |
| HL3 | 18 | BX073647/ii | OMM5056 | 1 | 0.00 | NS |
| HL3 | 18 | BX319197 | OMM5056 | 0 | 0.92 | NS |
| HL3 | 32 | BX870052/i | OMM5176 | 1 | 0.00 | NS |
| HL3 | 37 | OMM5014/ii | OMM5179 | 1 | 0^2^ | NS |
| Average |  |  |  | 3.47:1 | 63.21^2^ | p<0.001 |
|  |  |  |  |  |  |  |
| HL7 | 6 | BHMS206 | BHMS272 | 1 | 0 | NS |
| HL7 | 12/27 | CA345149 | OMM1345 | 0.31:1 | 0.94 | NS |
| HL7 | 13b | OMM5312i | OMM1210 | UnDef | 16.59 | <0.001^3^ |
| HL7 | 16 | OMM1195 | OMM5091 | 1 | 0 | NS |
| HL7 | 16 | OMM1195 | BX299451 | 0.48:1 | 0.29 | NS |
| HL7 | 16 | OMM5091 | BX299451 | 0.5:1 | 0.29 | NS |
| HL7 | 16 | BX299451 | CA060381 | 1 | 0.00 | NS |
| HL7 | 17 | Omi126TUF | OMM1445 | 12:1 | 11.00 | <0.001^3^ |
| HL7 | 23b | BX873441 | OmyRT16TUF | 3.43:1 | 7.80^2^ | <0.01 |
| HL7 | 24 | OMM1220 | BHMS465/i | 1.37:1 | 0.29^2^ | NS |
| HL7 | 24 | BHMS465/i | OMM5102/ii | 0.15:1 | 7.49 | <0.01 |
| HL7 | 18 | BX319197 | OMM5056 | 1.69:1 | 0.48 | NS |
| HL7 | 32 | BX870052/i | OMM5176 | 1 | 0.00 | NS |
| HL7 | 32 | BX870052/i | OMM1329 | 0.19:1 | 2.82 | NS |
| HL7 | 32 | OMM5176 | OMM1329 | 0.2:1 | 2.82 | NS |
| HL7 | 34 | BX319411/i | BX861121/ii | 0 | 3.59 | NS |
| HL7 | 35 | OMM1263/i | OMM5000/i | 2:1 | 0.63 | NS |
| HL7 | 36 | CA061336 | Sal5UoG | UnDef | 47.25 | <0.001^3^ |
| Average |  |  |  | 2.19:1 | 20.00^2^ | p<0.001 |

Part-B. Between family comparisons of sex-specific recombination rates for Hills Lake

brook charr mapping parents.

| Family | LG | Marker 1 | Marker 2 | Ratio (F:M) ^1^ | G-value^2^ | Significance^3^ |
| --- | --- | --- | --- | --- | --- | --- |
| HL3vsHL7 | 5 | Omi179TUF | OMM1372/ii | UnDef | 3.54 | N.S. |
| HL3vsHL7 | 6 | BHMS206 | BHMS272 | 1 | 0 | N.S. |
| HL3vsHL7 | 7 | BX073974 | OMM3075 | UnDef | 7.52 | <0.01 |
| HL3vsHL7 | 9 | Ssa0072BSFU | TC126859/i | UnDef | 15.51 | <0.001^3^ |
| HL3vsHL7 | 16 | CA060381 | BX299451 | UnDef | 11.02 | <0.001^3^ |
| HL3vsHL7 | 16 | CA060381 | OMM5091 | 8.56:1 | 8.09 | <0.005 |
| HL3vsHL7 | 16 | BX299451 | OMM5091 | 1.15:1 | 0 | N.S. |
| HL3vsHL7 | 16 | OMM5091 | OMM1195 | UnDef | 16.84 | <0.001^3^ |
| HL3vsHL7 | 23b | BX873441 | OmyRT16TUF | 2.44:1 | 3.46^2^ | N.S. |
| HL3vsHL7 | 24 | OMM1220 | BHMS465/i | 2.13:1 | 2.25^2^ | N.S. |
| HL3vsHL7 | 24 | BHMS465/i | OMM5102/ii | 0.15:1 | 7.74 | <0.01 |
| HL3vsHL7 | 18 | BX319197 | OMM5056 | 0 | 3.5 | N.S. |
| HL3vsHL7 | 32 | BX870052/i | OMM5176 | 1 | 0 | N.S. |
| HL3vsHL7 | 32 | BX870052/i | OMM1329 | 0.40:1 | 1.82 | N.S. |
| HL3vsHL7 | 32 | OMM5176 | OMM1329 | 0.40:1 | 1.88 | N.S. |
| Average |  |  |  | 2.41:1 | 21.56 ^2^ | <0.001 |
|  |  |  |  |  |  |  |
| HL7vsHL3 | 6 | BHMS206 | BHMS272 | 1 | 0 | N.S. |
| HL7vsHL3 | 10 | Omi30TUF/i | OmyRGT2TUF/i | 3.89:1 | 2.26 | N.S. |
| HL7vsHL3 | 13a | BHMS377/i | OMM5312/ii | 9.41:1 | 7.17 | <0.05 |
| HL7vsHL3 | 13b | OMM5312/i | OMM1210 | 0.56:1 | 3.1^2^ | N.S. |
| HL7vsHL3 | 15 | Omi30TUF/ii | OmyRGT2TUF/ii | UnDef | 25.16 | <0.001^3^ |
| HL7vsHL3 | 16 | BHMS417/i | OMM1195 | 0.04:1 | 25.89 | <0.001^3^ |
| HL7vsHL3 | 16 | OMM1195 | BX299451 | 0.14:1 | 4.46 | <0.05 |
| HL7vsHL3 | 16 | BX299451 | CA060381 | 0 | 7.99 | <0.005 |
| HL7vsHL3 | 17 | Omi126TUF | OMM1445 | UnDef | 16.5 | <0.001^3^ |
| HL7vsHL3 | 22 | BX313739 | Ssa0080BSFU/ii | 3.91:1 | 7.61 | <0.01 |
| HL7vsHL3 | 23b | BX873441 | OmyRT16TUF | 9.47:1 | 15.49 | <0.001^3^ |
| HL7vsHL3 | 18 | BX319197 | OMM5056 | 4.89:1 | 2.61 | N.S. |
| HL7vsHL3 | 32 | BX870052/i | OMM5176 | 1 | 0 | N.S. |
| HL7vsHL3 | 35 | OMM1263/i | OMM5000/i | UnDef | 5.08 | <0.05 |
| Average |  |  |  | 1.60:1 | 9.88^2^ | <0.005 |

^1^Indicates female(F) : male(M) recombination ratio for the pair of markers indicated. UnDef indicates no

recombination in the male parent while 0 indicates no recombination in the female parent.

^2^Indicates G-test was not corrected for small sample size.

^3^Indicates significance at p<0.05 following Bonferroni correction.
