# Supplementary material for: Genome evolution in the fish family salmonidae: generation of a brook charr genetic map and comparisons among charrs (Arctic charr and brook charr) with rainbow trout
Source: BMC Genet. 2011 Jul 28;12:68. doi: 10.1186/1471-2156-12-68 (PMC3162921; doi:10.1186/1471-2156-12-68)
Supplement: Additional file 6 — Intra-sex comparisons of the recombination rates between the Hills Lake brook charr female and male mapping parents. [file 1471-2156-12-68-S6.DOC]

Additional File 5. – Part A. Comparison of recombination rates between the

Hills Lake brook charr female mapping parents.

| LG | Marker 1 | Marker 2 | Ratio^1^ | G-value^2^ | Significance^3^ |
| --- | --- | --- | --- | --- | --- |
| 3 | BHMS465/ii | OMM5102/i | UnDef | 3.30 | N.S. |
| 6 | BHMS206 | BHMS272 | 1 | 0 | N.S. |
| 10 | Omi30TUF/i | OmyRGT2TUF/i | 0.26:1 | 2.26 | N.S. |
| 13a | BHMS377/i | OMM5312/ii | 1.73:1 | 2.38^2^ | N.S. |
| 14 | BHMS238 | OMM5113 | 0.73:1 | 0.10 | N.S. |
| 16 | BHMS331 | Ssa0033BSFU | 8.67:1 | 6.94 | <0.01 |
| 16 | CA060381 | BHMS417/i | 1.09:1 | 0 | N.S. |
| 16 | CA060381 | BX299451 | UnDef | 11.02 | <0.001^3^ |
| 16 | BHMS417/i | BX299451 | 6.12:1 | 3.41 | N.S. |
| 16 | BX299451 | OMM5091 | 2.29:1 | 0.41 | N.S. |
| 16 | OMM5091 | OMM1195 | UnDef | 16.84 | <0.001^3^ |
| 23b | BX873441 | OmyRT16TUF | 0.71:1 | 1^2^ | N.S. |
| 30 | OMM1205 | OMM3015/i | 2.18:1 | 2.42^2^ | N.S. |
| 24 | OMM1220 | BHMS465/i | 1.56:1 | 0.93^2^ | N.S. |
| 24 | BHMS465/i | OMM5102/ii | 0.96:1 | 0 | N.S. |
| 18 | BX319197 | OMM5056 | 0 | 6.23 | <0.05 |
| 32 | BX870052/i | OMM5176 | 1 | 0 | N.S. |
| 32 | BX870052/i | OMM1329 | 2.12:1 | 0.41 | N.S. |
| 32 | OMM5176 | OMM1329 | 2:1 | 0.38 | N.S. |
| 43 | OMM5007 | OMM5008 | 1.09:1 | 0.09^2^ | N.S. |
| Average |  |  | 1.49:1 | 8.81^2^ | P<0.005 |

^1^Indicates HL3 female:HL7 female recombination ratio for the pair of markers indicated.

UnDef indicates no recombination in the HL7 female while 0 indicates no recombination in the

HL3 female.

^2^Indicates G-test was not corrected for small sample size.

^3^Indicates significance at p<0.05 following Bonferroni correction.

Additional File 6. – Part B. Comparison of recombination rates between the

Hills Lake brook charr male mapping parents.

| LG | Marker 1 | Marker 2 | Ratio^1^ | G-value^2^ | Significance^3^ |
| --- | --- | --- | --- | --- | --- |
| 3 | OMM5102/i | SalF41SFU | UnDef | 7.23 | <0.01 |
| 4 | BX881655 | TC126859/ii | 1.46:1 | 1.10^2^ | N.S. |
| 5 | Omi179TUF | OMM1372/ii | UnDef | 0.91 | N.S. |
| 6 | BHMS206 | BHMS272 | 1 | 0 | N.S. |
| 8 | BX305863 | CA368462/ii | 0 | 0.91 | N.S. |
| 9 | Ssa0072BSFU | TC126859/i | UnDef | 5.11 | <0.05 |
| 13b | OMM1210 | OMM5312/ii | UnDef | 30.91 | <0.001^3^ |
| 15 | OmyRGT2TUF/ii | OMM1237/i | 1 | 0 | N.S. |
| 15 | OmyRGT2TUF/ii | OMM1512 | 0.94:1 | 0.02^2^ | N.S. |
| 15 | OMM1237/i | OMM1512 | 0.98:1 | 0^2^ | N.S. |
| 16 | CA060381 | BX299451 | UnDef | 7.99 | <0.005 |
| 16 | CA060381 | OMM1195 | 11.56:1 | 13.05 | <0.001^3^ |
| 16 | BX299451 | OMM1195 | 3.42:1 | 2.56 | N.S. |
| 17 | Omi126TUF | OMM1445 | 0 | 0.92 | N.S. |
| 20a | BX890355/i | OMM5019/i | 0.12:1 | 51.59^2^ | <0.001^3^ |
| 21 | OMM1201 | BX311884/i | 0.078:1 | 19.15 | <0.001^3^ |
| 21 | BX311884/i | Omy21INRA/ii | 0 | 1.3 | N.S. |
| 22 | OkeSLINRA | Ssa0080BSFU/ii | 1 | 0 | N.S. |
| 23b | BX873441 | OmyRT16TUF | 0.36:1 | 1.46 | N.S. |
| 25b | BX079862 | BX319197 | 0 | 0.86 | N.S. |
| 25b | BX079862 | OMM5056 | 0 | 2.18 | N.S. |
| 25b | BX079862 | SalE38SFU | 1 | 0 | N.S. |
| 25b | BX319197 | OMM5056 | 0.35:1 | 0.87 | N.S. |
| 25b | BX319197 | SalE38SFU | 0 | 0.88 | N.S. |
| 25b | OMM5056 | SalE38SFU | 0 | 2.21 | N.S. |
| 32 | BX870052/i | OMM5176 | 1 | 0 | N.S. |
| 35 | OMM1263/i | OMM5000/i | 0 | 2.27 | N.S. |
| Average |  |  | 0.87:1 | 1.22^2^ | N.S. |

^1^Indicates HL3 male:HL7 male recombination ratio for the pair of markers indicated. UnDef

indicates no recombination in the HL7 male while 0 indicates no recombination in the HL3 male.

^2^Indicates G-test was not corrected for small sample size.

^3^Indicates significance at p<0.05 following Bonferroni correction.
