# Supplementary material for: Genome evolution in the fish family salmonidae: generation of a brook charr genetic map and comparisons among charrs (Arctic charr and brook charr) with rainbow trout
Source: BMC Genet. 2011 Jul 28;12:68. doi: 10.1186/1471-2156-12-68 (PMC3162921; doi:10.1186/1471-2156-12-68)
Supplement: Additional file 7 — Observed deviations from Mendelian expectations in the brook charr mapping parents of families HL3 and HL7. [file 1471-2156-12-68-S7.DOC]

Additional File 6. Observed deviations from Mendelian expectations in the brook

charr mapping parents of families HL3 and HL7.

^1^Significant following Bonferroni correction

| Linkage group | Family | Sex | Marker | N | G test value | P |
| --- | --- | --- | --- | --- | --- | --- |
| 3 | HL7 | M | OMM5102/i | 70 | 47.286 | P<0.001^1,2^ |
| 4 | HL7 | F | BX881655 | 113 | 6.514 | P<0.05 |
| 5 | HL7 | M | Omi179TUF | 113 | 3.925 | P<0.05 |
| 5 | HL7 | M | OMM1372/ii | 113 | 3.925 | P<0.05 |
| 6 | HL3 | M | BHMS206 | 115 | 3.856 | P<0.05 |
| 6/35 | LN4 | F | OMM5000/ii | 104 | 3.87 | P<0.05 |
| 8/UNA | LN4 | F | CA368462/ii | 103 | 5.179 | P<0.05 |
| 9 | HL3 | F | TC126859/i | 111 | 3.997 | P<0.05 |
| 11 | HL3 | M | CA344270 | 111 | 3.997 | P<0.05 |
| 12 | HL3 | F | BX345149 | 115 | 5.478 | P<0.05 |
| 12 | HL3 | F | OMM5161/ii | 115 | 3.856 | P<0.05 |
| 13a | HL3 | F | BHMS377/i | 74 | 27.975 | P<0.001^1,2^ |
| 13a | HL3 | M | OMM5312/ii | 88 | 7.797 | P<0.01^2^ |
| 14 | HL7 | M | OMM5113 | 113 | 5.577 | P<0.05 |
| 15 | HL3 | F | CR363293 | 113 | 7.526 | P<0.01 |
| 15 | HL7 | F | OMM1197/ii | 65 | 22.378 | P<0.001^1,2^ |
| 16 | HL7 | F | BHMS331 | 113 | 4.714 | P<0.05 |
| 16 | HL7 | F | Ssa0033BSFU | 109 | 4.071 | P<0.05 |
| 16 | HL3 | M | BHMS417/ii | 84 | 6.953 | P<0.01^2^ |
| 16 | HL3 | F | CA060381 | 115 | 3.856 | P<0.05 |
| 16 | HL7 | M | OMM1195 | 113 | 5.577 | P<0.05 |
| 16 | HL3 | F | OMM1195 | 113 | 3.925 | P<0.05 |
| 16 | HL7 | F | OMM5014/i | 112 | 4.349 | P<0.05 |
| 16 | HL3 | F | OMM5091 | 109 | 5.785 | P<0.05 |
| 16 | HL7 | M | OMM5091 | 111 | 5.679 | P<0.05 |
| 16 | LN4 | M | OMM5091 | 109 | 5.785 | P<0.05 |
| 20a | HL7 | F | CA376300/i | 57 | 68.95 | P<0.001^1,2^ |
| 20a | HL7 | M | CA376300/i | 19 | 13.552 | P<0.001^1,2^ |
| 20a | HL7 | M | CA376300/ii | 19 | 13.552 | P<0.001^1,2^ |
| 24 | LN4 | M | BHMS465/ii | 90 | 10.193 | P<0.01^1,2^ |
| 26 | HL7 | M | SalD25SFU | 111 | 5.679 | P<0.05 |
| 35 | HL3 | M | OMM1263/i | 115 | 3.856 | P<0.05 |
| UNA | HL7 | F | BHMS417/iii | 89 | 4.087 | P<0.05^1^ |
| 4+2 | HL3 | M | OMM1228 | 115 | 3.856 | P<0.05 |

^2^Large proportion of progeny not scored
