# Supplementary material for: Genome evolution in the fish family salmonidae: generation of a brook charr genetic map and comparisons among charrs (Arctic charr and brook charr) with rainbow trout
Source: BMC Genet. 2011 Jul 28;12:68. doi: 10.1186/1471-2156-12-68 (PMC3162921; doi:10.1186/1471-2156-12-68)
Supplement: Additional file 8 — Comparative genetic maps between Arctic charr, brook charr, and rainbow trout, using the combined female of Arctic charr as a template. [file 1471-2156-12-68-S8.PDF]

Additional File 8

Conserved synteny blocks among rainbow trout (RT),brook charr (BC) and Arctic charr linkage groups. The composite Arctic charr female map is used as a template. If additional markers are present in the male mapping parents, they are listed below the female maps. Male markers were not incorporated into the genetic maps given the large recombination differences between the sexes in salmonid fishes.

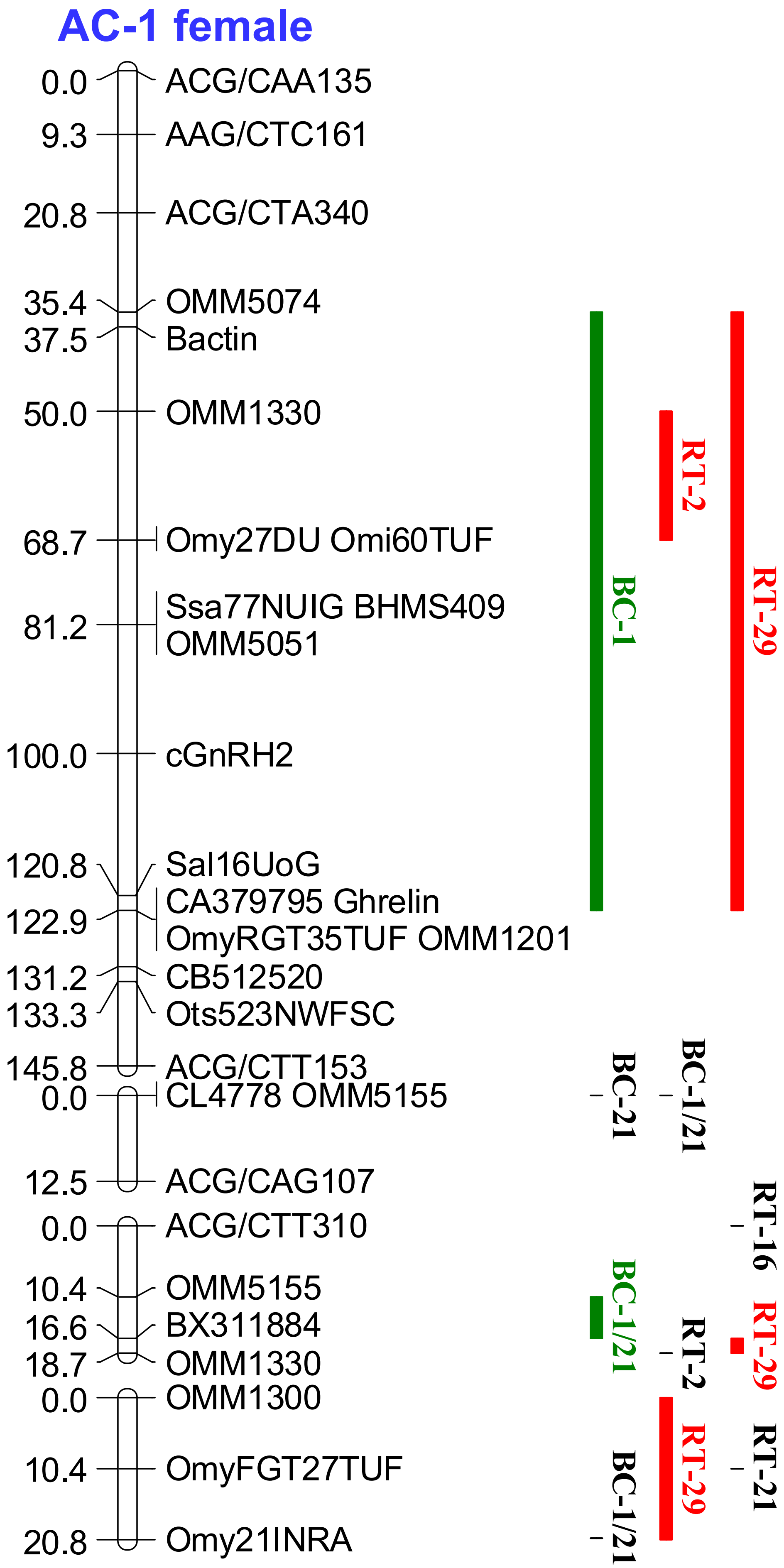
AC-1 female
0.0 ACG/CAA135
9.3 AAG/CTC161
20.8 ACG/CTA340
35.4 OMM5074
37.5 Bactin
50.0 OMM1330
68.7 Omy27DU Omi60TUF
81.2 Ssa77NUIG BHMS409 OMM5051
100.0 cGnRH2
120.8 Sal16UoG
122.9 CA379795 Ghrelin OmyRGT35TUF OMM1201
131.2 CB512520
133.3 Ots523NWFSC
145.8 ACG/CTT153
0.0 CL4778 OMM5155
12.5 ACG/CAG107
0.0 ACG/CTT310
10.4 OMM5155
16.6 BX311884
18.7 OMM1330
0.0 OMM1300
10.4 OmyFGT27TUF
20.8 Omy21INRA
BC-1
RT-2
RT-29
BC-21
BC-1/21
RT-16
BC-1/21
RT-2
RT-29
RT-29
RT-21
BC-1/21

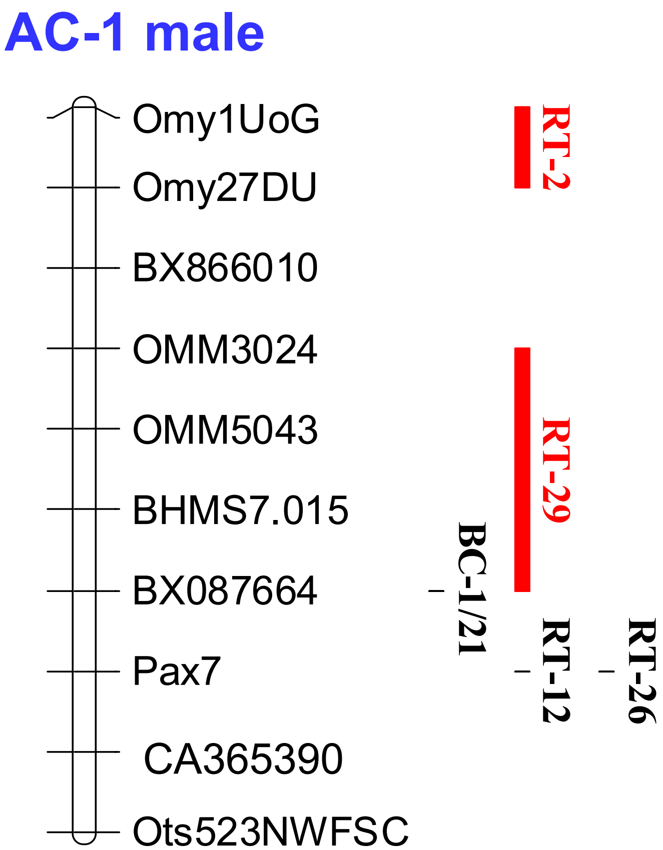
AC-1 male
Omy1UoG
Omy27DU
BX866010
OMM3024
OMM5043
BHMS7.015
BX087664
Pax7
CA365390
Ots523NWFSC
RT-2
RT-29
BC-1/21
RT-12
RT-26

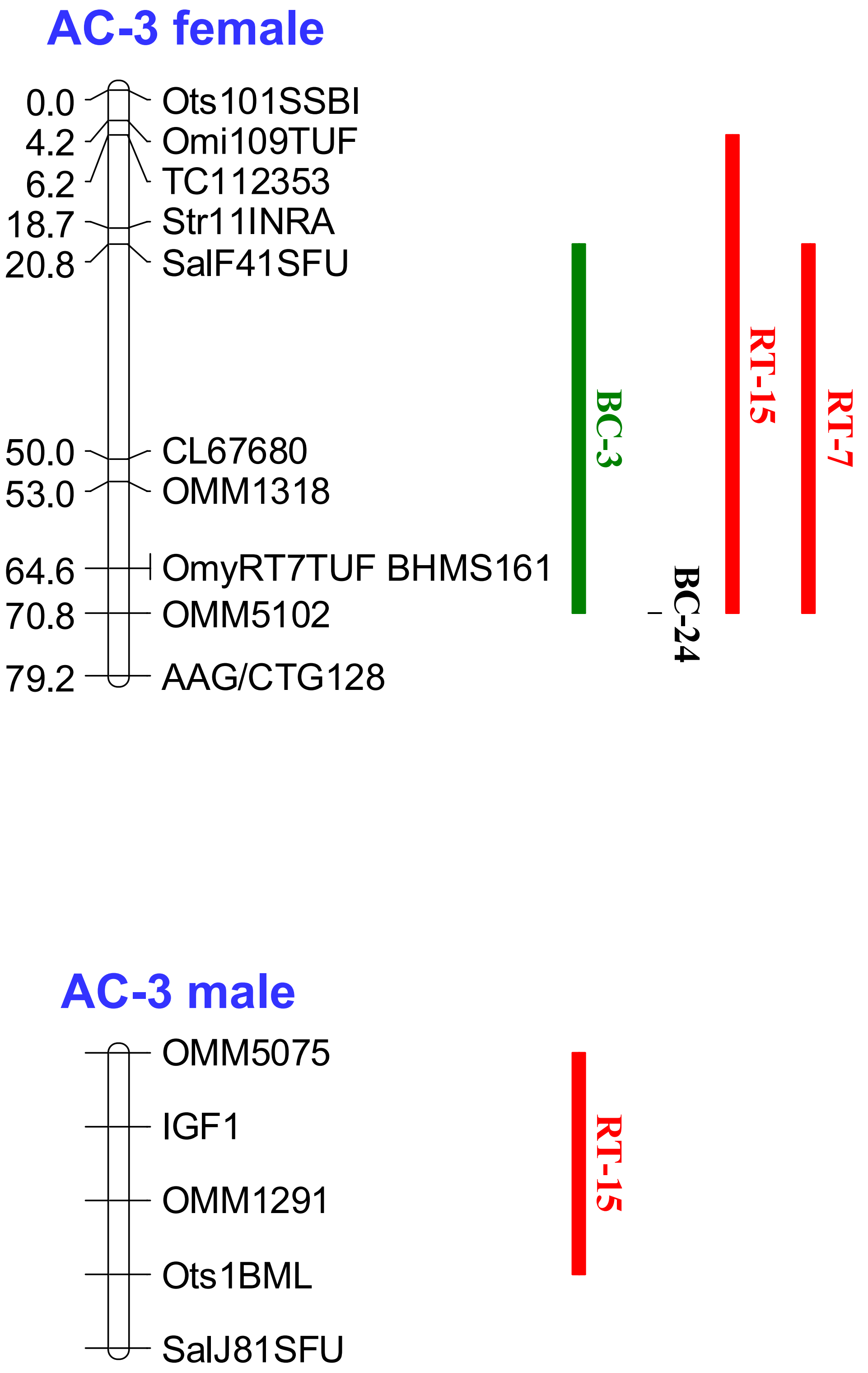
AC-3 female
0.0
Ots101SSBI
4.2
Omi109TUF
6.2
TC112353
18.7
Str11INRA
20.8
SalF41SFU
50.0
CL67680
53.0
OMM1318
64.6
OmyRT7TUF BHMS161
70.8
OMM5102
79.2
AAG/CTG128
BC-3
RT-15
RT-7
BC-24
AC-3 male
OMM5075
IGF1
OMM1291
Ots1BML
SalJ81SFU
RT-15

AC-4 female

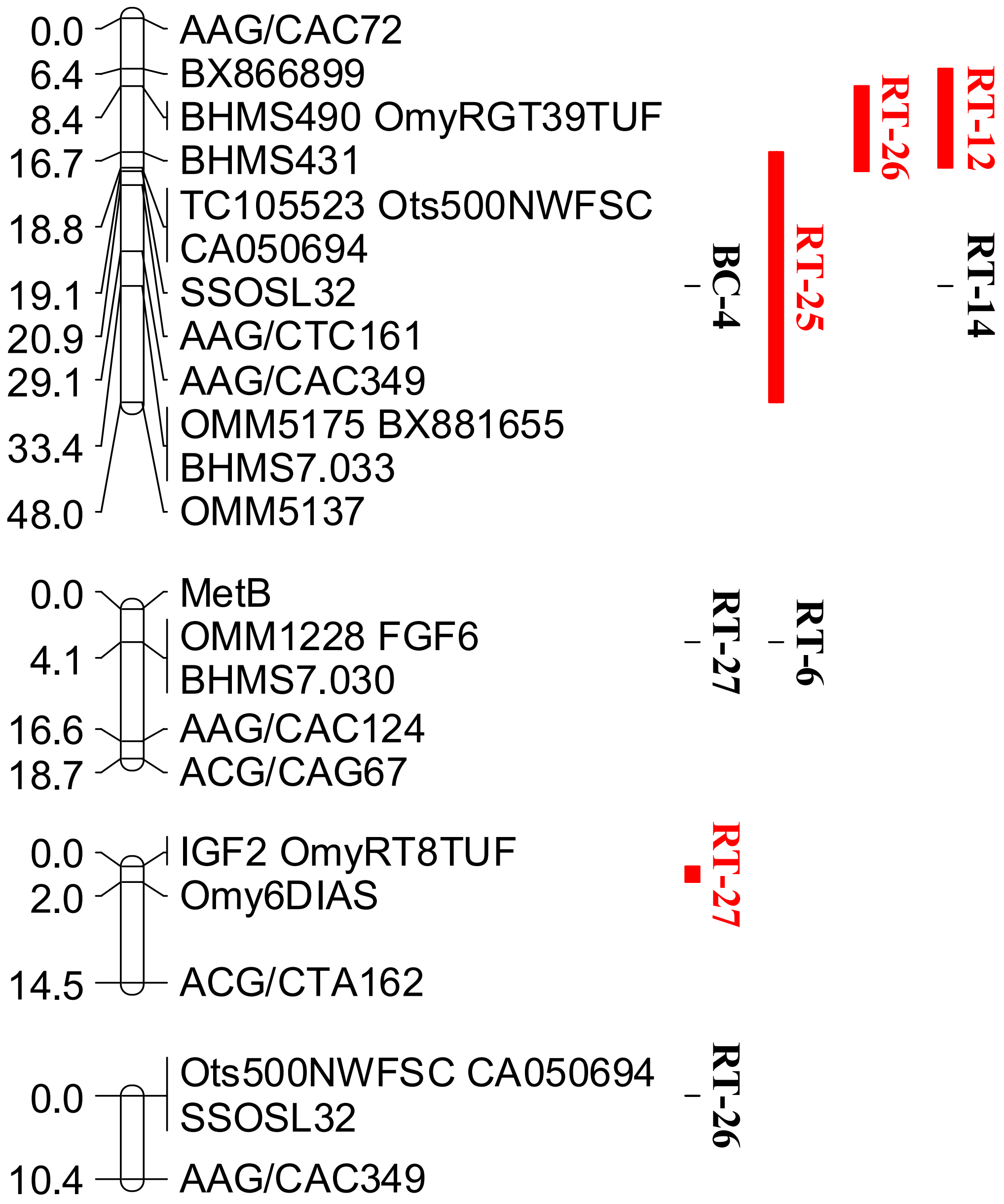
0.0 AAG/CAC72
6.4 BX866899
8.4 BHMS490 OmyRGT39TUF
16.7 BHMS431
18.8 TC105523 Ots500NWFSC
CA050694
19.1 SSOSL32
20.9 AAG/CTC161
29.1 AAG/CAC349
33.4 OMM5175 BX881655
BHMS7.033
48.0 OMM5137
RT-12
RT-26
RT-25
BC-4
RT-14
0.0 MetB
OMM1228 FGF6
4.1 BHMS7.030
16.6 AAG/CAC124
18.7 ACG/CAG67
RT-27
RT-6
0.0 IGF2 OmyRT8TUF
2.0 Omy6DIAS
14.5 ACG/CTA162
RT-27
0.0 Ots500NWFSC CA050694
SSOSL32
10.4 AAG/CAC349
RT-26

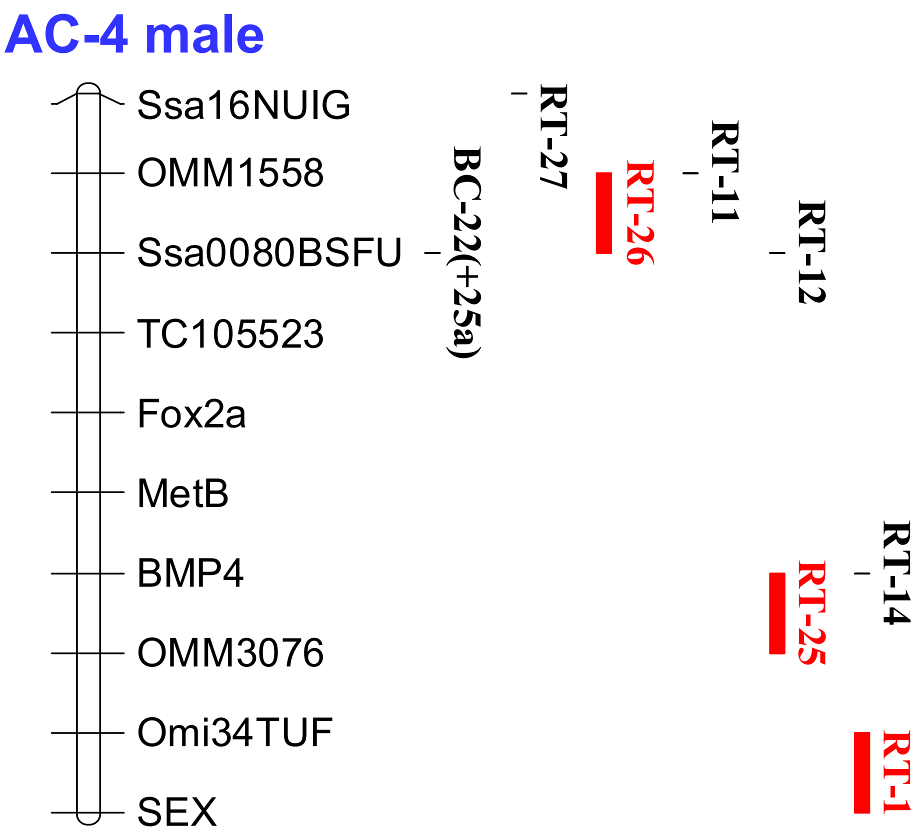
AC-4 male
Ssa16NUIG
OMM1558
Ssa0080BSFU
TC105523
Fox2a
MetB
BMP4
OMM3076
Omi34TUF
SEX
BC-22(+25a)
RT-27
RT-26
RT-11
RT-12
RT-14
RT-25
RT-1

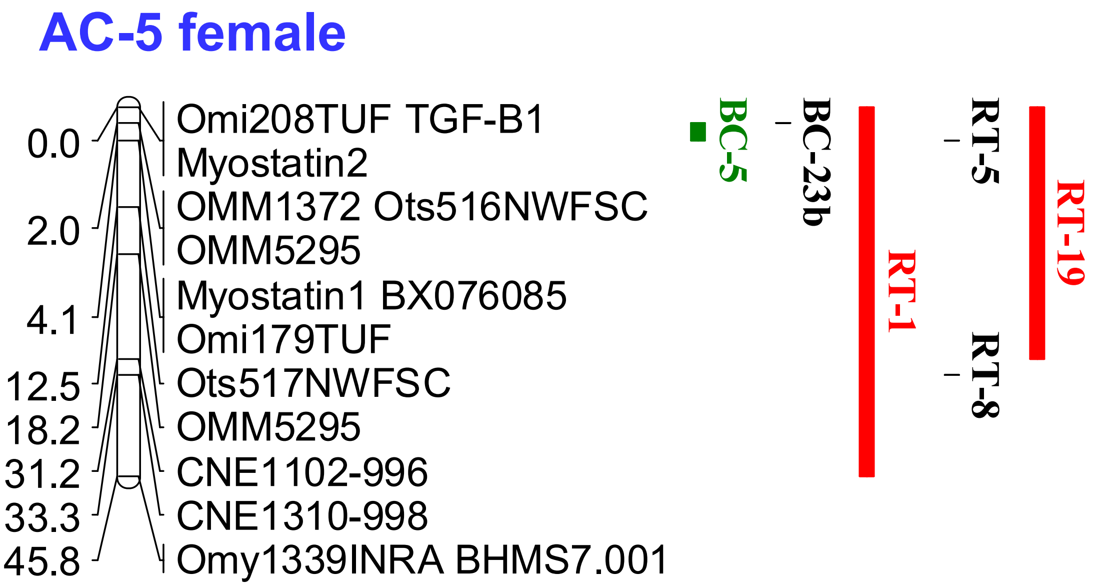
AC-5 female
0.0
2.0
4.1
12.5
18.2
31.2
33.3
45.8
Omi208TUF TGF-B1
Myostatin2
OMM1372 Ots516NWFSC
OMM5295
Myostatin1 BX076085
Omi179TUF
Ots517NWFSC
OMM5295
CNE1102-996
CNE1310-998
Omy1339INRA BHMS7.001
BC-5
BC-23b
RT-1
RT-5
RT-8
RT-19

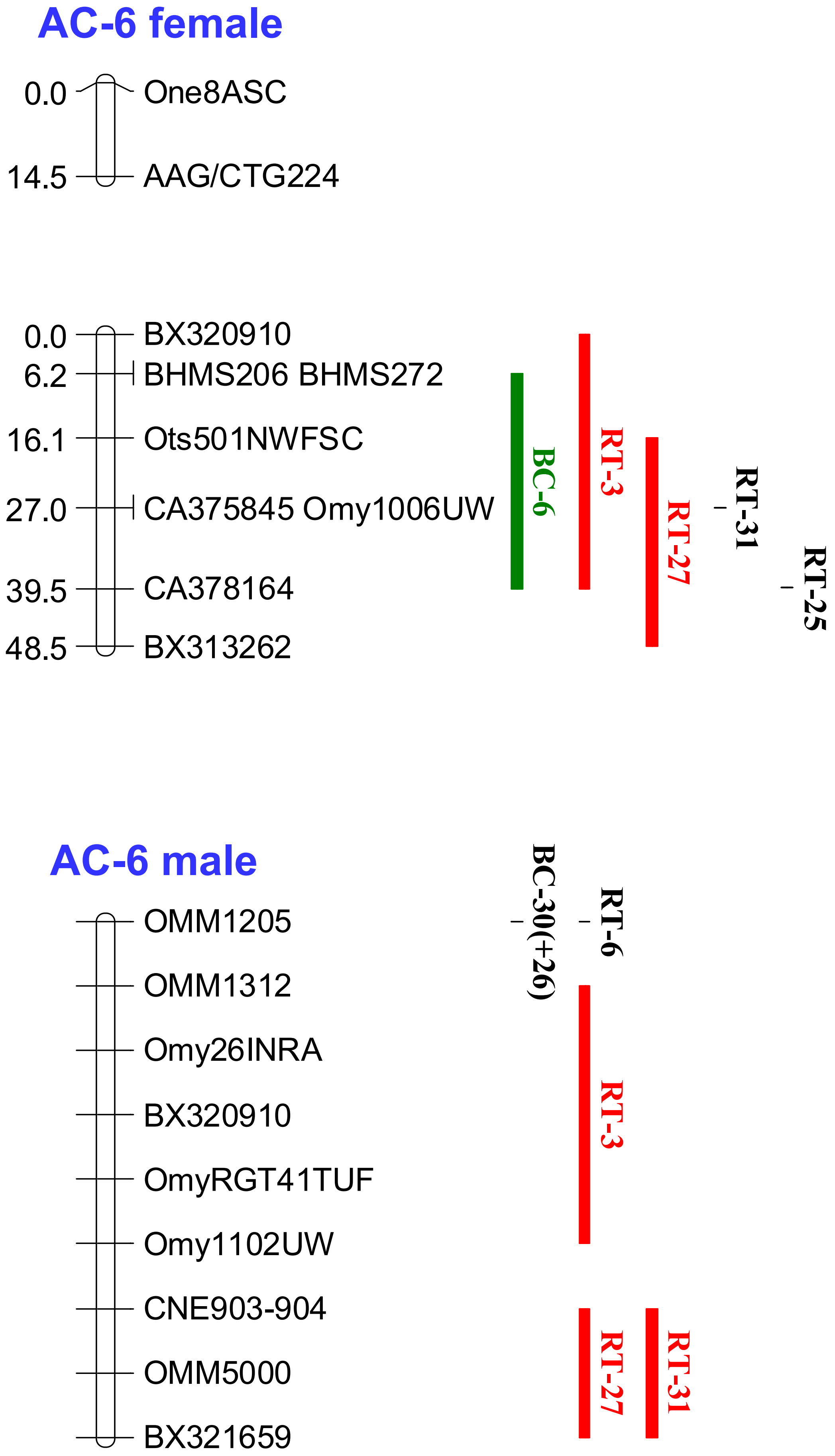
AC-6 female
0.0
One8ASC
14.5
AAG/CTG224
0.0
BX320910
6.2
BHMS206 BHMS272
16.1
Ots501NWFSC
27.0
CA375845 Omy1006UW
39.5
CA378164
48.5
BX313262
BC-6
RT-3
RT-27
RT-31
RT-25
AC-6 male
OMM1205
OMM1312
Omy26INRA
BX320910
OmyRGT41TUF
Omy1102UW
CNE903-904
OMM5000
BX321659
BC-30(+26)
RT-6
RT-3
RT-27
RT-31

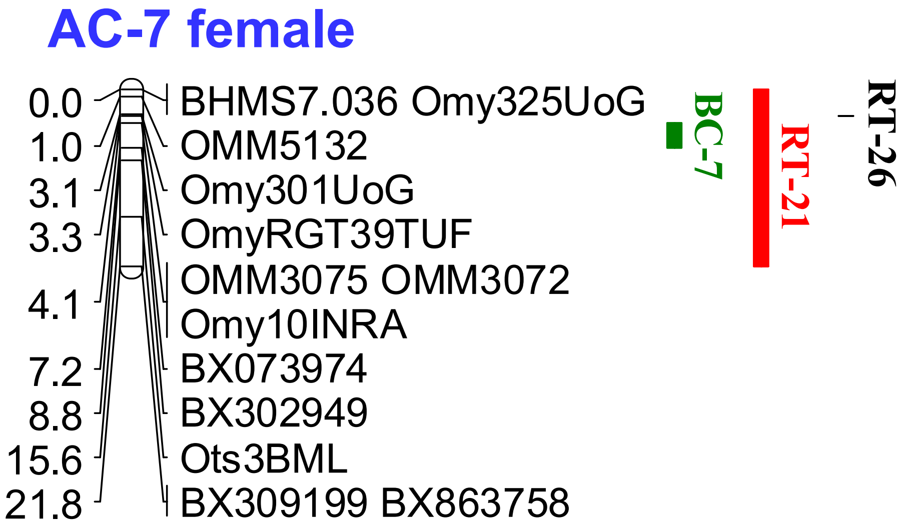
AC-7 female
0.0
1.0
3.1
3.3
4.1
7.2
8.8
15.6
21.8
BHMS7.036 Omy325UoG
OMM5132
Omy301UoG
OmyRGT39TUF
OMM3075 OMM3072
Omy10INRA
BX073974
BX302949
Ots3BML
BX309199 BX863758
BC-7
RT-21
RT-26

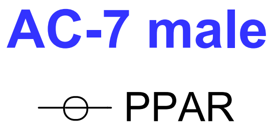
AC-7 male
PPAR

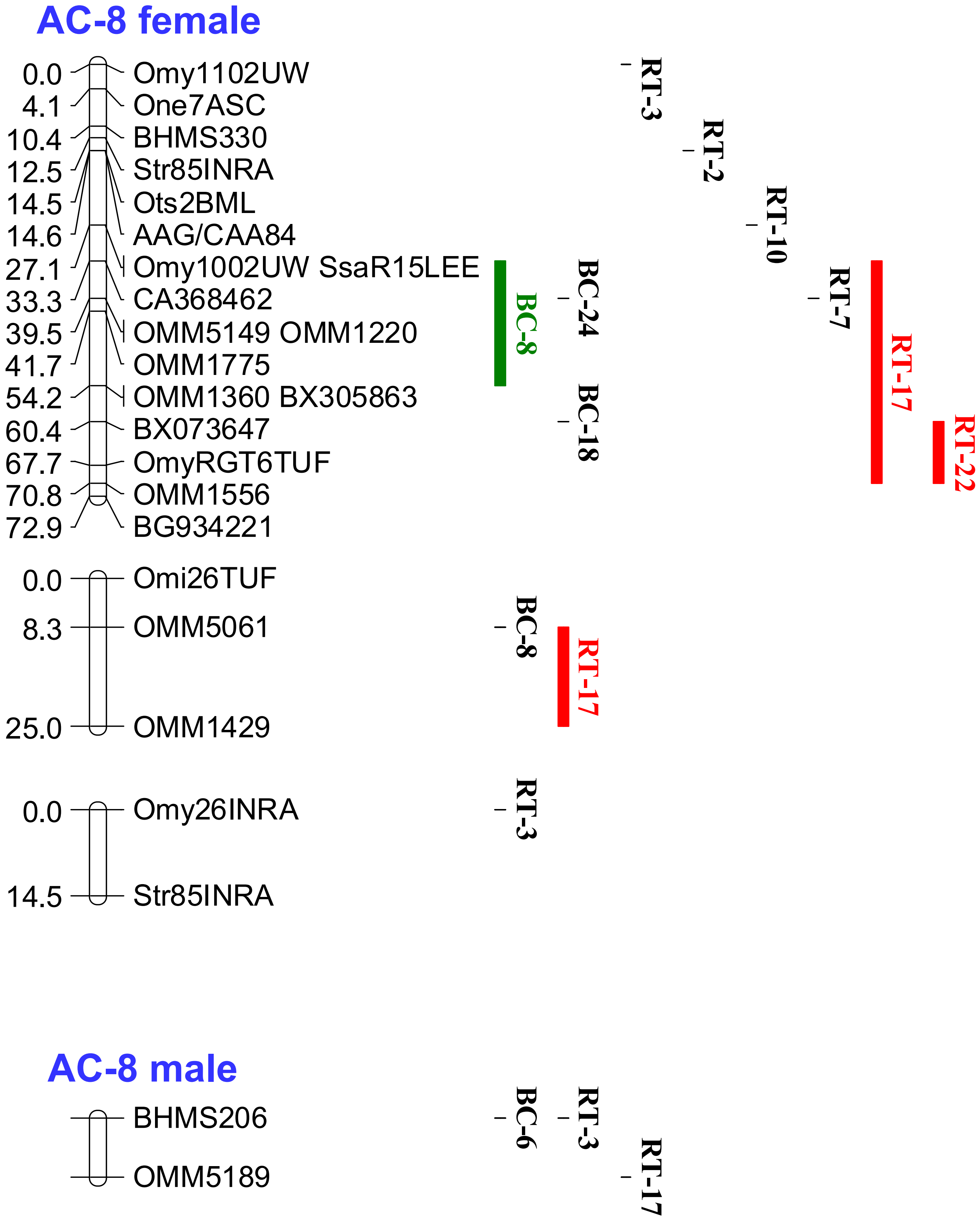
AC-8 female
0.0 Omy1102UW
4.1 One7ASC
10.4 BHMS330
12.5 Str85INRA
14.5 Ots2BML
14.6 AAG/CAA84
27.1 Omy1002UW SsaR15LEE
33.3 CA368462
39.5 OMM5149 OMM1220
41.7 OMM1775
54.2 OMM1360 BX305863
60.4 BX073647
67.7 OmyRGT6TUF
70.8 OMM1556
72.9 BG934221
RT-3
RT-2
RT-10
RT-7
RT-17
RT-22
BC-8
BC-24
BC-18
0.0 Omi26TUF
8.3 OMM5061
25.0 OMM1429
BC-8
RT-17
0.0 Omy26INRA
14.5 Str85INRA
RT-3
AC-8 male
BHMS206
OMM5189
BC-6
RT-3
RT-17

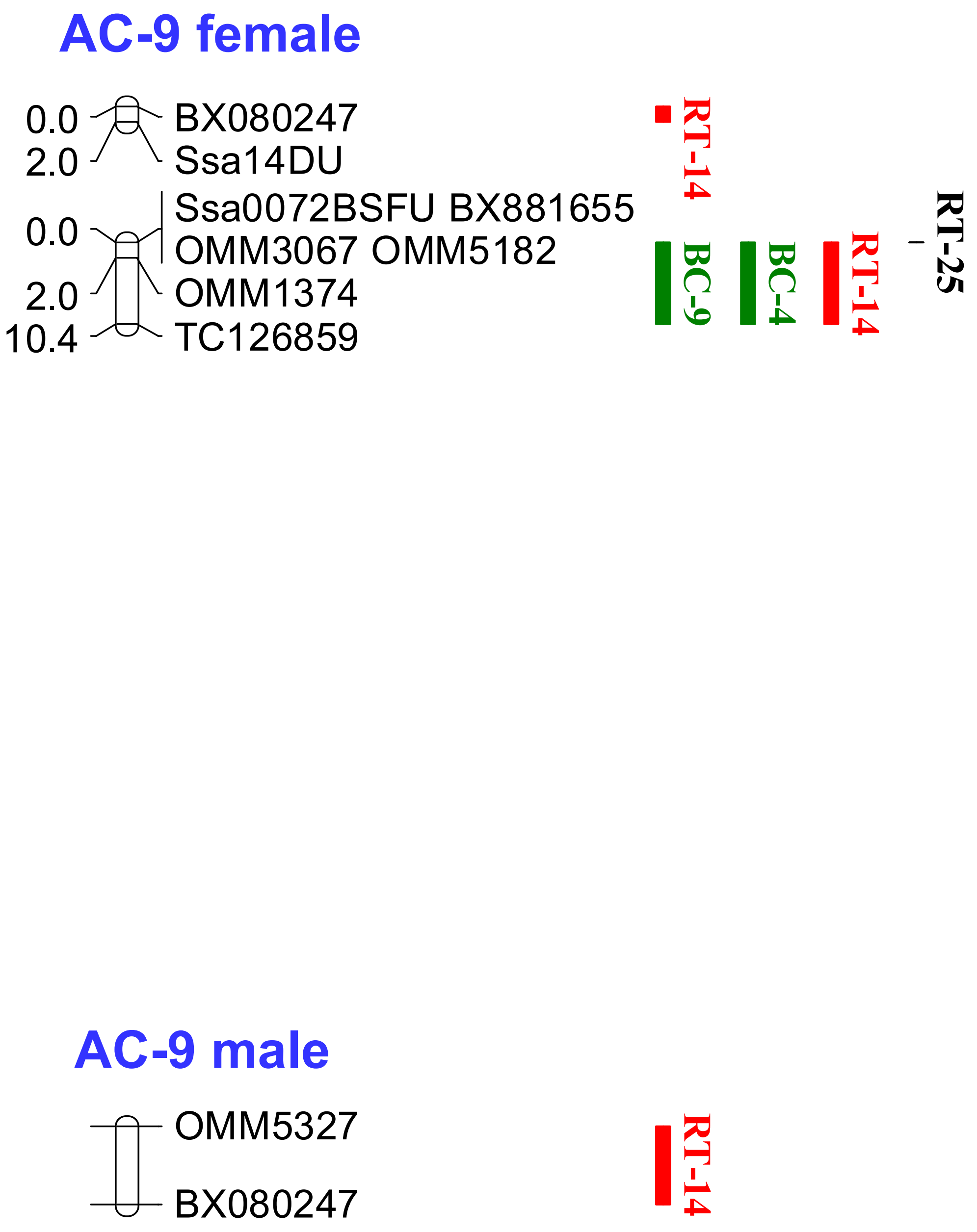
AC-9 female
0.0
2.0
BX080247
Ssa14DU
RT-14
0.0
2.0
10.4
Ssa0072BSFU BX881655
OMM3067 OMM5182
OMM1374
TC126859
BC-9
BC-4
RT-14
RT-25
AC-9 male
OMM5327
BX080247
RT-14

AC-10 female

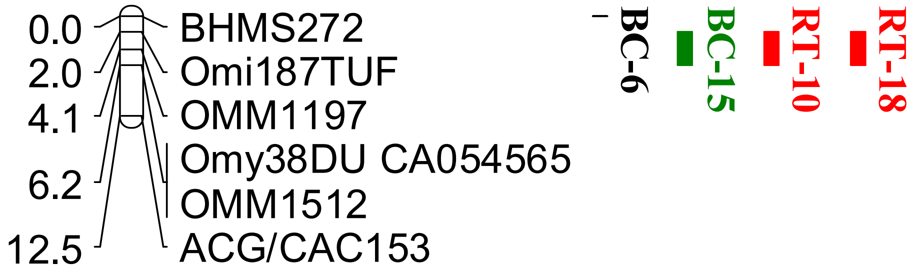

AC-10 male

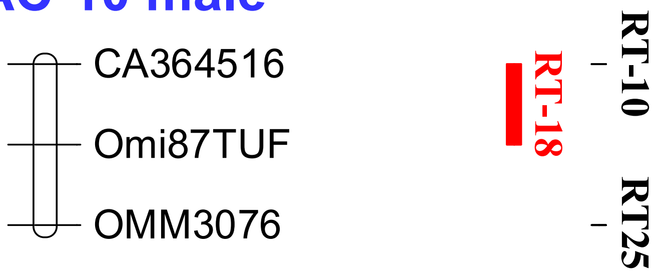

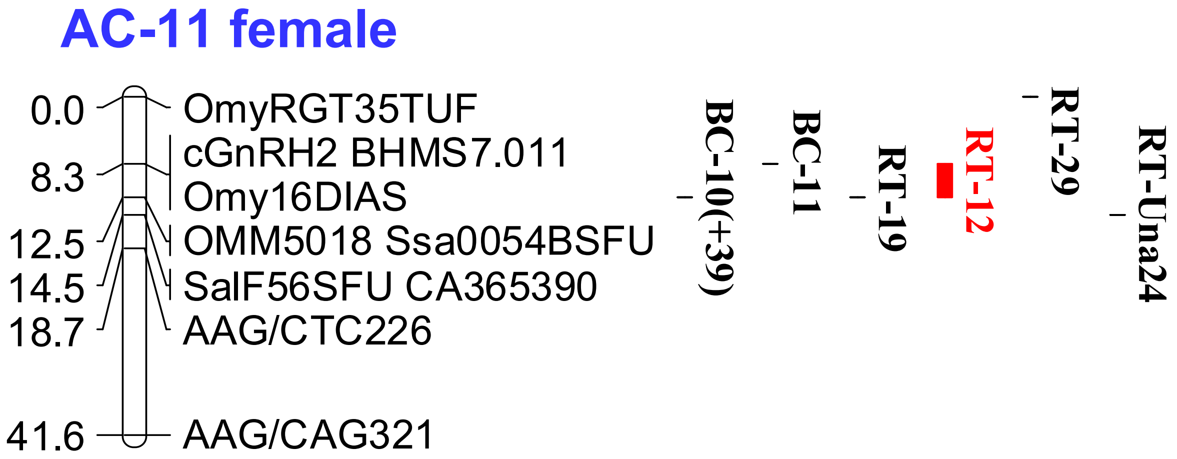
AC-11 female
0.0
8.3
12.5
14.5
18.7
41.6
OmyRGT35TUF
cGnRH2 BHMS7.011
Omy16DIAS
OMM5018 Ssa0054BSFU
SalF56SFU CA365390
AAG/CTC226
AAG/CAG321
BC-10(+39)
BC-11
RT-19
RT-12
RT-29
RT-Una24

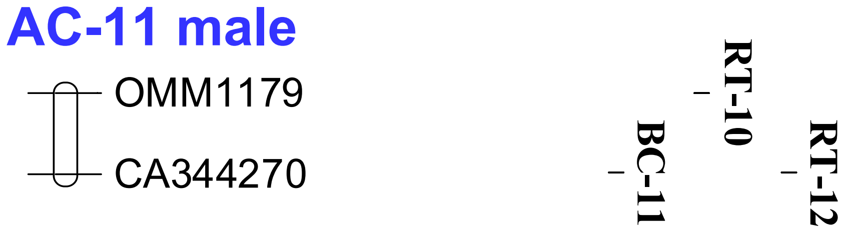
AC-11 male
OMM1179
CA344270
BC-11
RT-10
RT-12

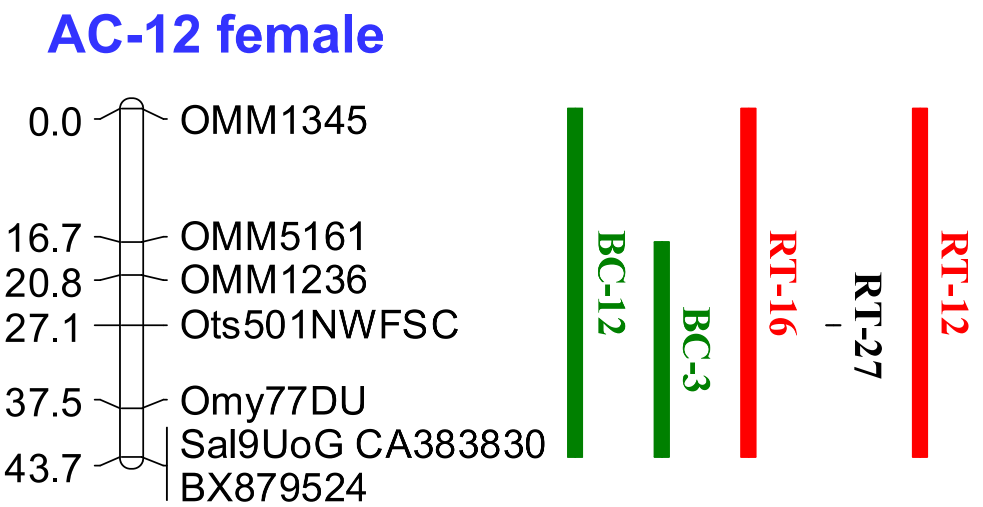
AC-12 female
0.0
OMM1345
16.7
OMM5161
20.8
OMM1236
27.1
Ots501NWFSC
37.5
Omy77DU
Sal9UoG CA383830
43.7
BX879524
BC-12
BC-3
RT-16
RT-27
RT-12

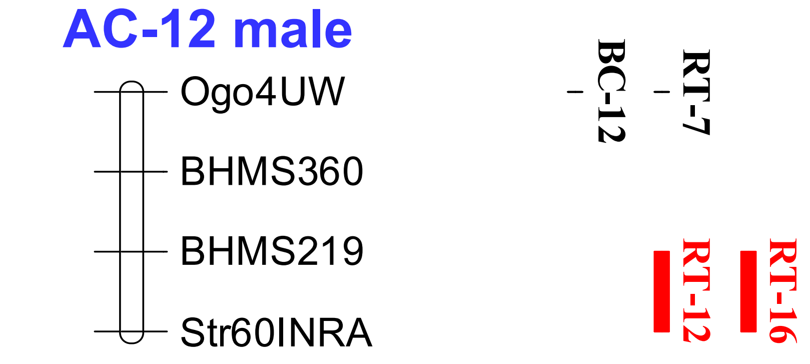
AC-12 male
Ogo4UW
BHMS360
BHMS219
Str60INRA
BC-12
RT-7
RT-12
RT-16

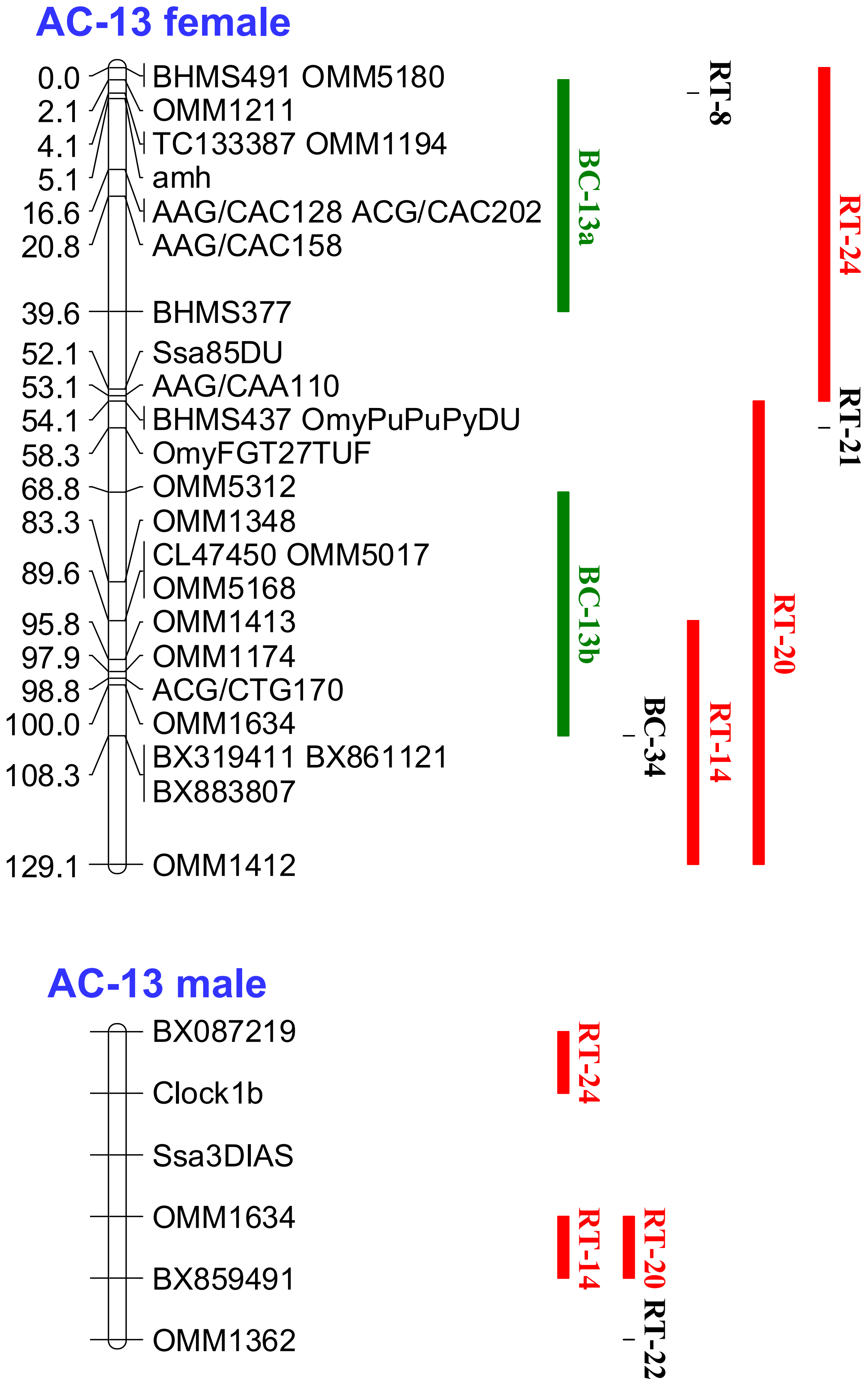
AC-13 female
0.0 BHMS491 OMM5180
2.1 OMM1211
4.1 TC133387 OMM1194
5.1 amh
16.6 AAG/CAC128 ACG/CAC202
20.8 AAG/CAC158
39.6 BHMS377
52.1 Ssa85DU
53.1 AAG/CAA110
54.1 BHMS437 OmyPuPuPyDU
58.3 OmyFGT27TUF
68.8 OMM5312
83.3 OMM1348
89.6 CL47450 OMM5017
OMM5168
95.8 OMM1413
97.9 OMM1174
98.8 ACG/CTG170
100.0 OMM1634
108.3 BX319411 BX861121
BX883807
129.1 OMM1412
BC-13a
BC-13b
RT-8
RT-24
RT-21
RT-20
RT-14
BC-34
AC-13 male
BX087219
Clock1b
Ssa3DIAS
OMM1634
BX859491
OMM1362
RT-24
RT-14
RT-20
RT-22

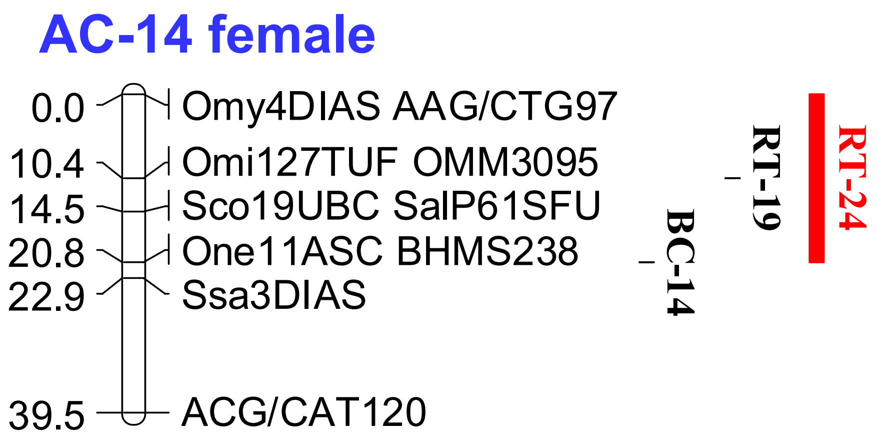
AC-14 female
0.0
10.4
14.5
20.8
22.9
39.5
Omy4DIAS AAG/CTG97
Omi127TUF OMM3095
Sco19UBC SalP61SFU
One11ASC BHMS238
Ssa3DIAS
ACG/CAT120
BC-14
RT-19
RT-24

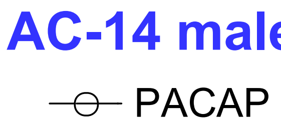
AC-14 male
PACAP

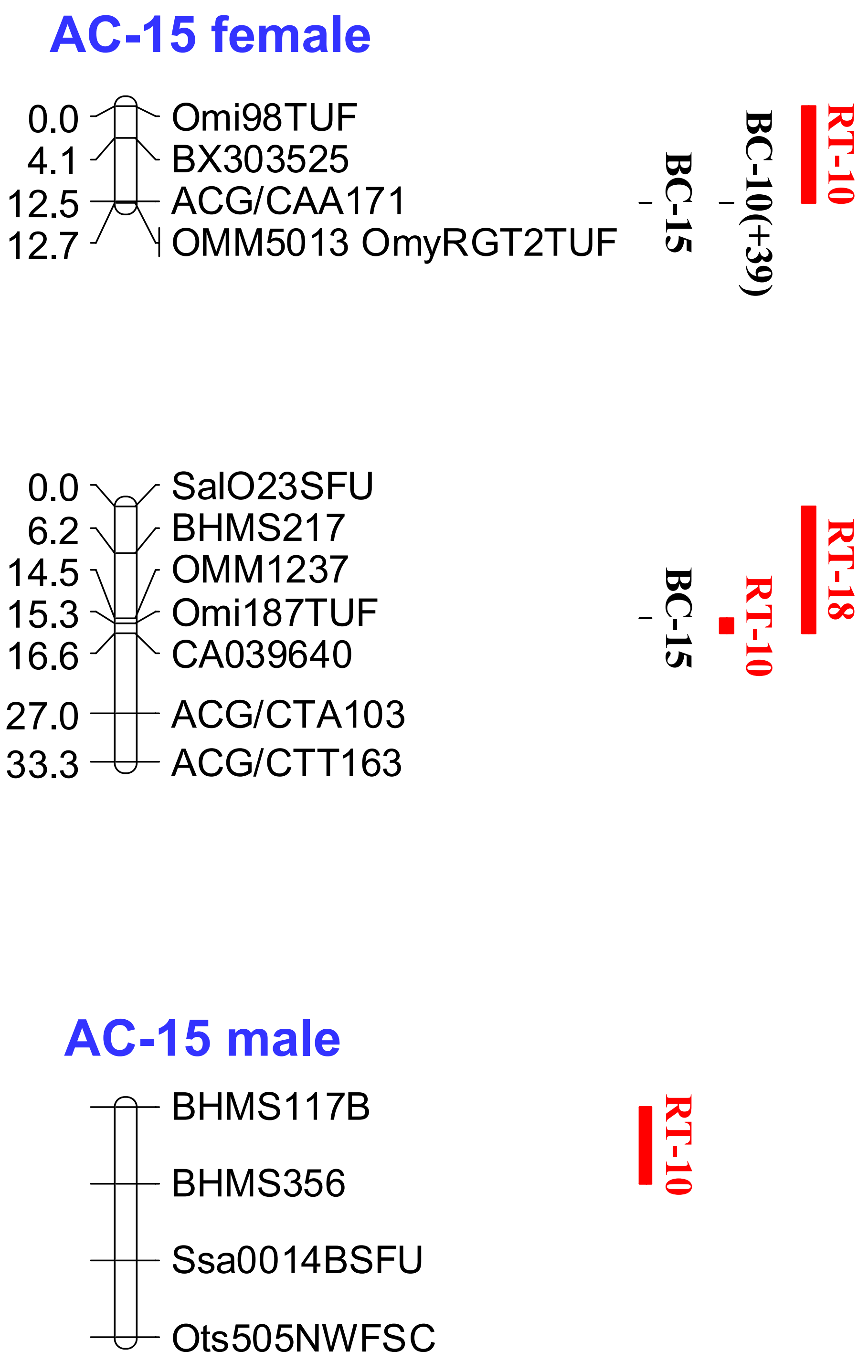
AC-15 female
0.0
4.1
12.5
12.7
Omi98TUF
BX303525
ACG/CAA171
OMM5013 OmyRGT2TUF
BC-15
BC-10(+39)
RT-10
0.0
6.2
14.5
15.3
16.6
27.0
33.3
SalO23SFU
BHMS217
OMM1237
Omi187TUF
CA039640
ACG/CTA103
ACG/CTT163
BC-15
RT-10
RT-18
AC-15 male
BHMS117B
BHMS356
Ssa0014BSFU
Ots505NWFSC
RT-10

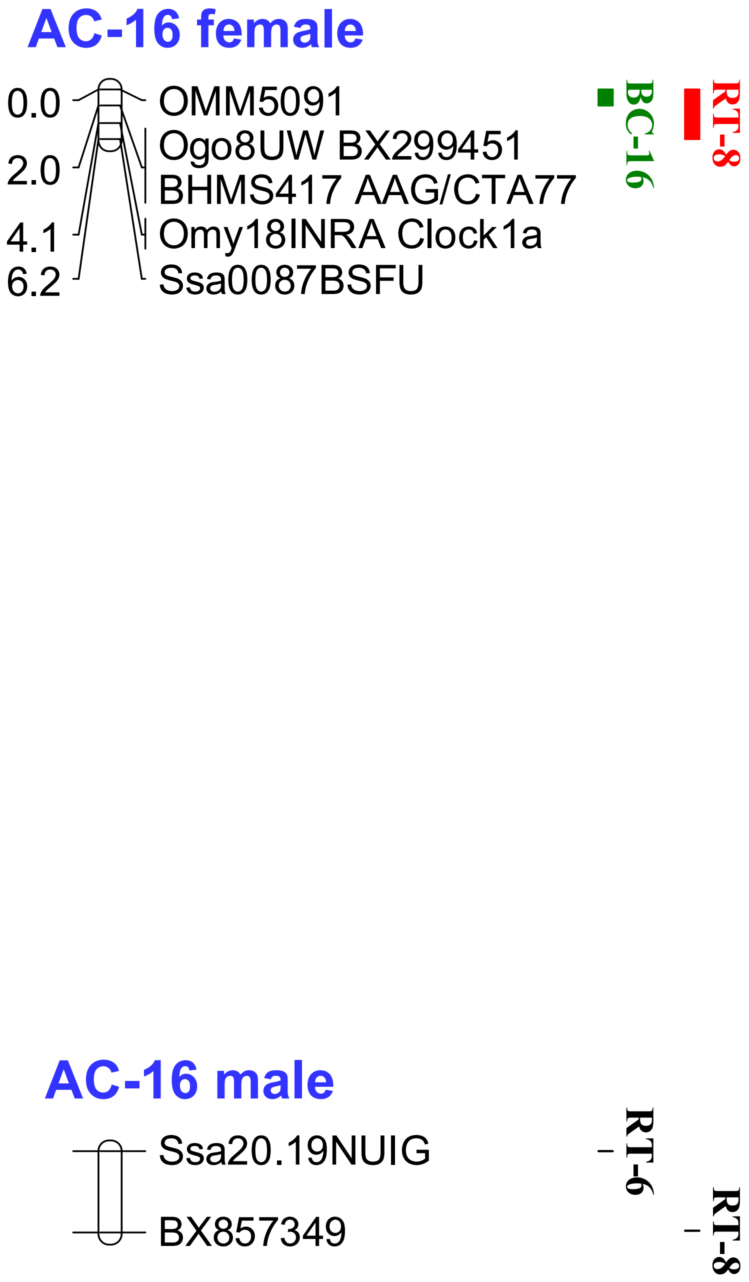
AC-16 female
0.0
2.0
4.1
6.2
OMM5091
Ogo8UW BX299451
BHMS417 AAG/CTA77
Omy18INRA Clock1a
Ssa0087BSFU
BC-16
RT-8
AC-16 male
Ssa20.19NUIG
BX857349
RT-6
RT-8

AC-17 female

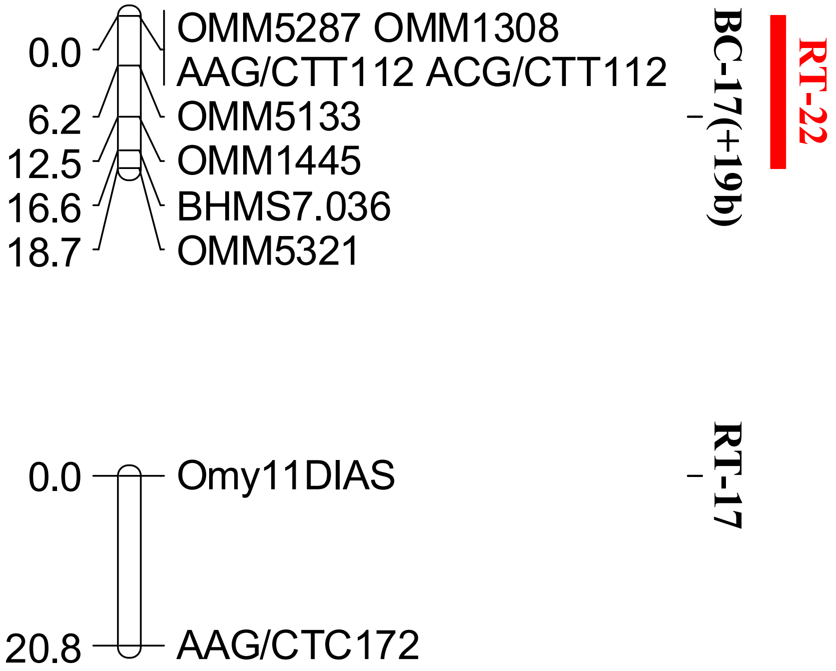

AC-17 male

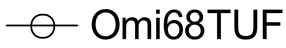

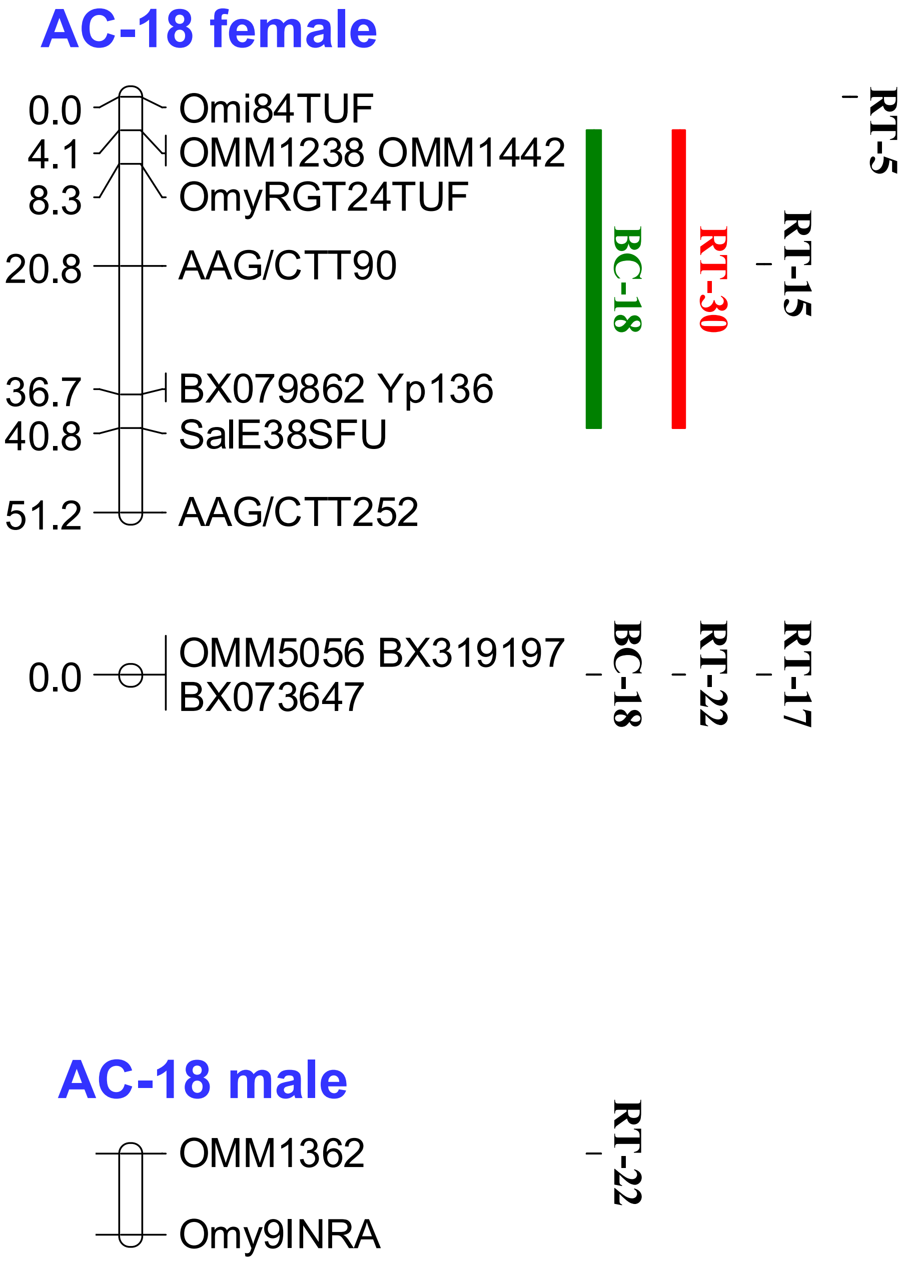
AC-18 female
0.0
4.1
8.3
20.8
36.7
40.8
51.2
Omi84TUF
OMM1238 OMM1442
OmyRGT24TUF
AAG/CTT90
BX079862 Yp136
SalE38SFU
AAG/CTT252
BC-18
RT-30
RT-15
RT-5
0.0
OMM5056 BX319197
BX073647
BC-18
RT-22
RT-17
AC-18 male
OMM1362
Omy9INRA
RT-22

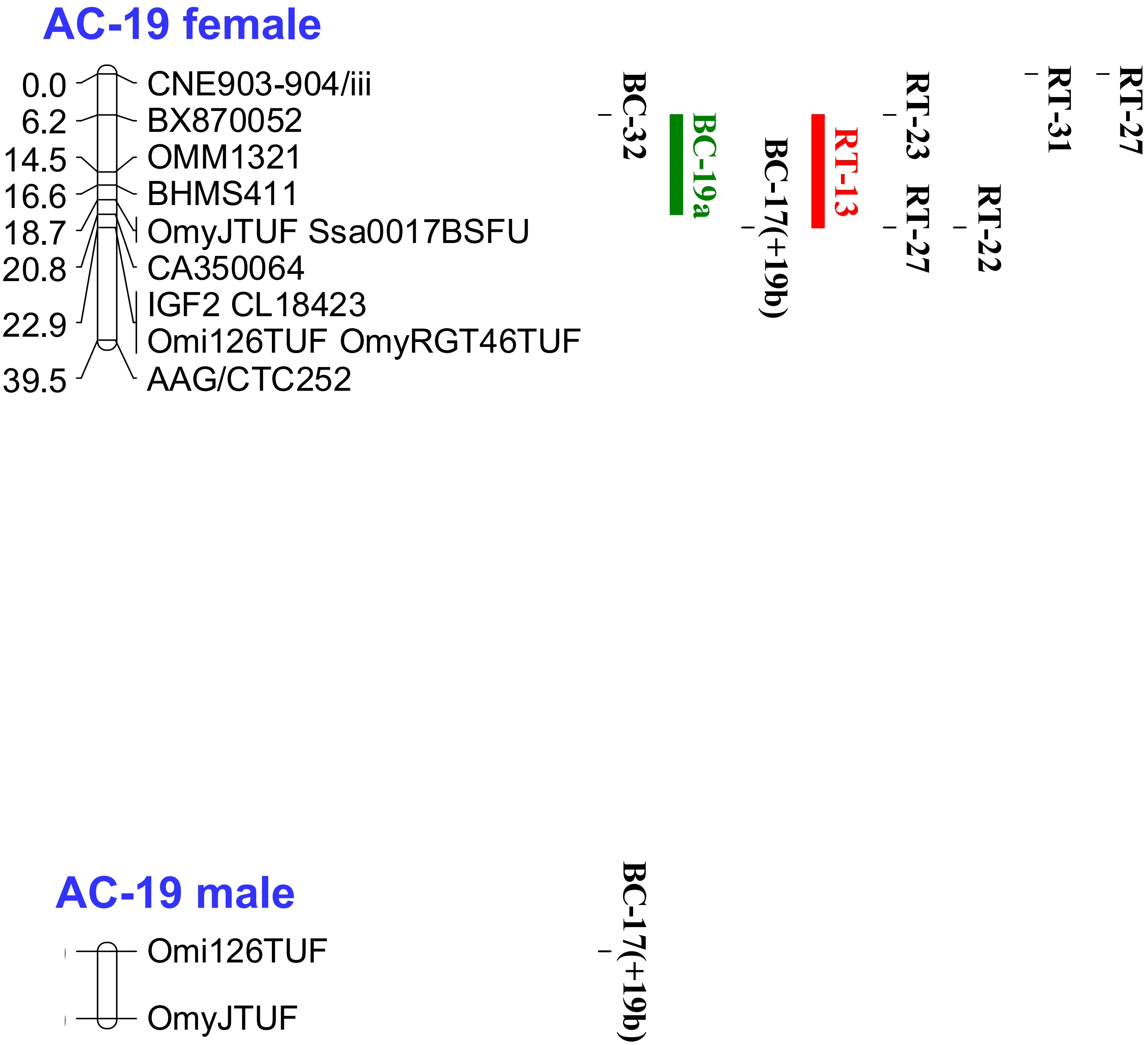

AC-19 female
0.0
6.2
14.5
16.6
18.7
20.8
22.9
39.5
CNE903-904/iii
BX870052
OMM1321
BHMS411
OmyJTUF Ssa0017BSFU
CA350064
IGF2 CL18423
Omi126TUF OmyRGT46TUF
AAG/CTC252
BC-32
BC-19a
BC-17(+19b)
RT-13
RT-23
RT-27
RT-22
RT-31
RT-27
AC-19 male
Omi126TUF
OmyJTUF
BC-17(+19b)

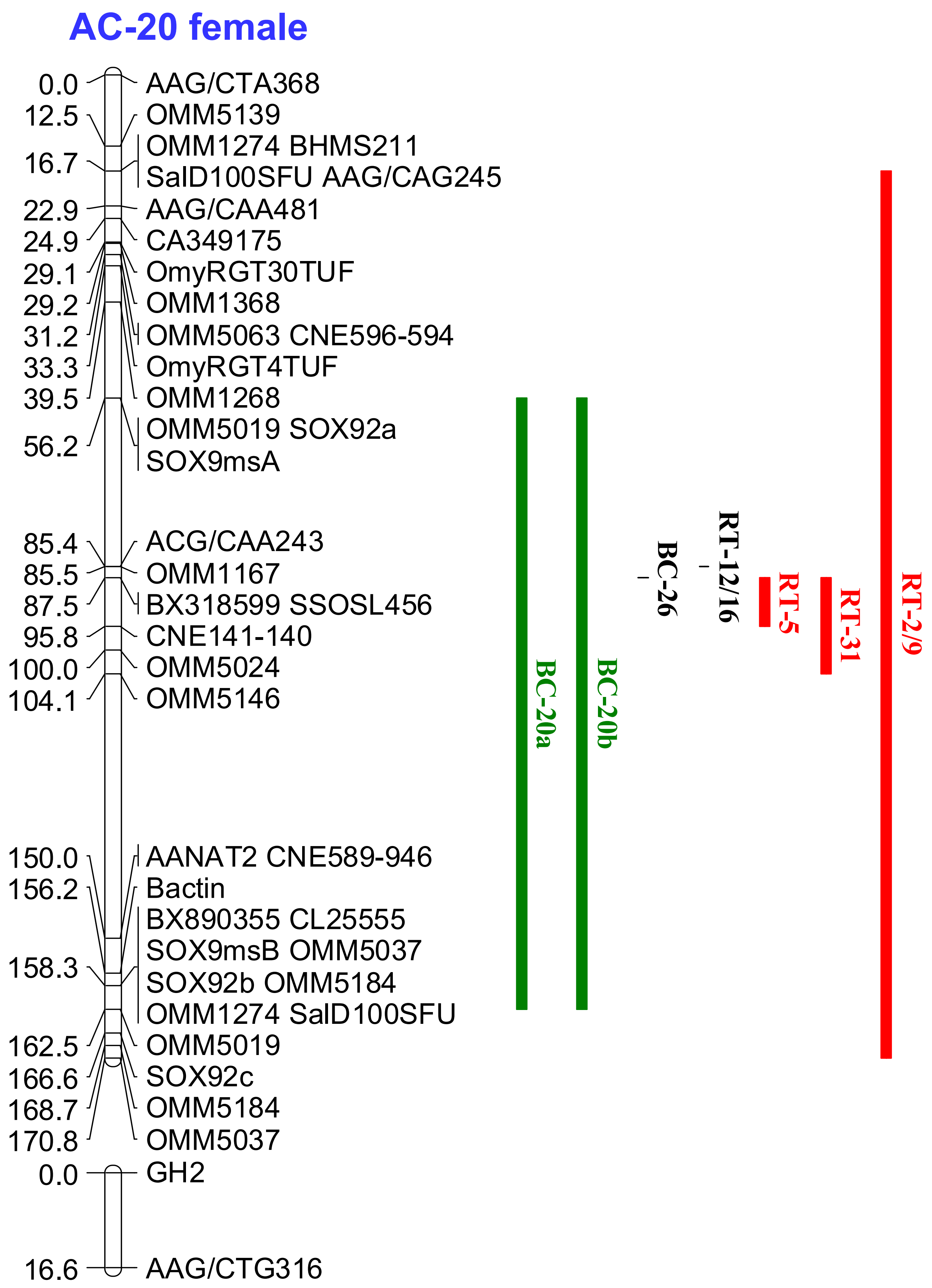
AC-20 female
0.0 AAG/CTA368
12.5 OMM5139
OMM1274 BHMS211
16.7 SalD100SFU AAG/CAG245
22.9 AAG/CAA481
24.9 CA349175
29.1 OmyRGT30TUF
29.2 OMM1368
31.2 OMM5063 CNE596-594
33.3 OmyRGT4TUF
39.5 OMM1268
OMM5019 SOX92a
56.2 SOX9msA
85.4 ACG/CAA243
85.5 OMM1167
87.5 BX318599 SSOSL456
95.8 CNE141-140
100.0 OMM5024
104.1 OMM5146
150.0 AANAT2 CNE589-946
156.2 Bactin
BX890355 CL25555
SOX9msB OMM5037
158.3 SOX92b OMM5184
OMM1274 SalD100SFU
162.5 OMM5019
166.6 SOX92c
168.7 OMM5184
170.8 OMM5037
0.0 GH2
16.6 AAG/CTG316
BC-20a
BC-20b
BC-26
RT-12/16
RT-5
RT-31
RT-2/9

AC-20 male

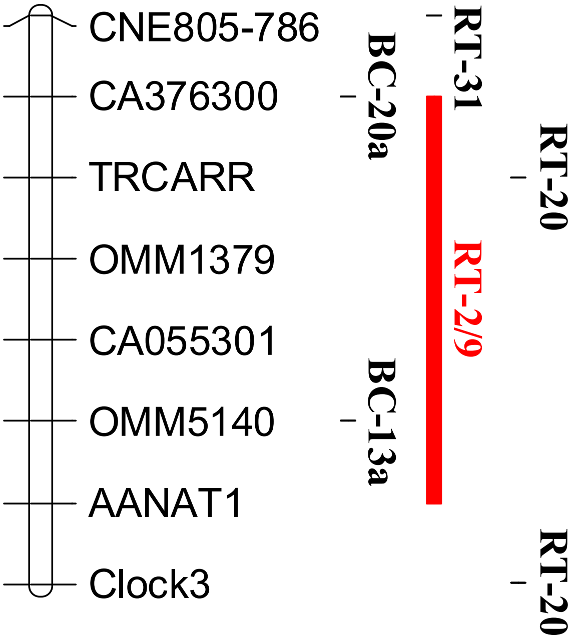

CNE805-786
CA376300
TRCARR
OMM1379
CA055301
OMM5140
AANAT1
Clock3
BC-20a
BC-13a
RT-31
RT-2/9
RT-20
RT-20

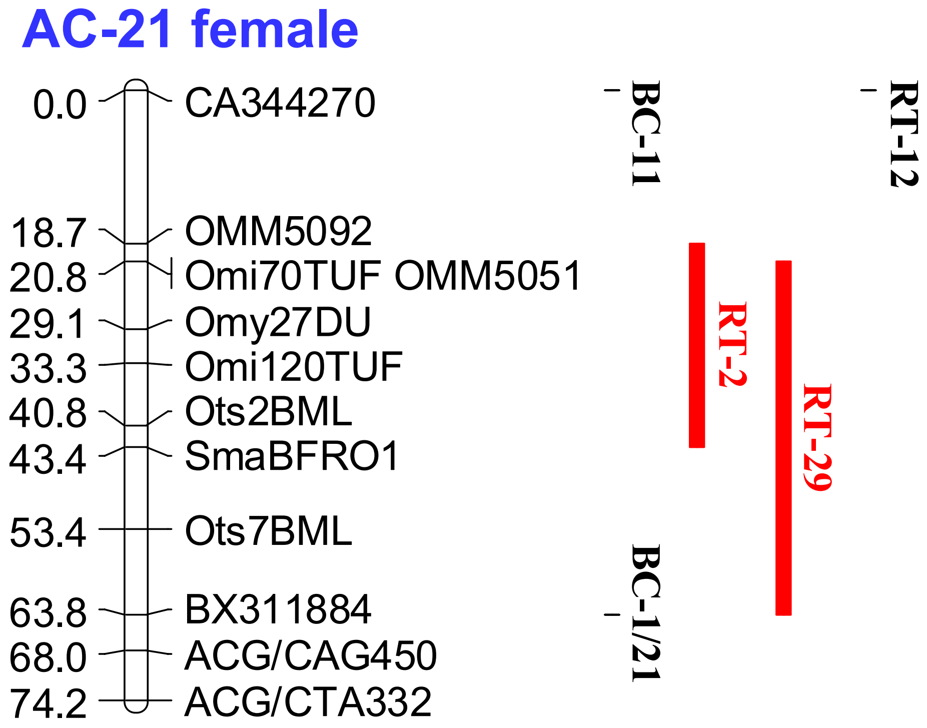
AC-21 female
0.0 CA344270
18.7 OMM5092
20.8 Omi70TUF OMM5051
29.1 Omy27DU
33.3 Omi120TUF
40.8 Ots2BML
43.4 SmaBFRO1
53.4 Ots7BML
63.8 BX311884
68.0 ACG/CAG450
74.2 ACG/CTA332
BC-11
RT-12
RT-2
RT-29
BC-1/21

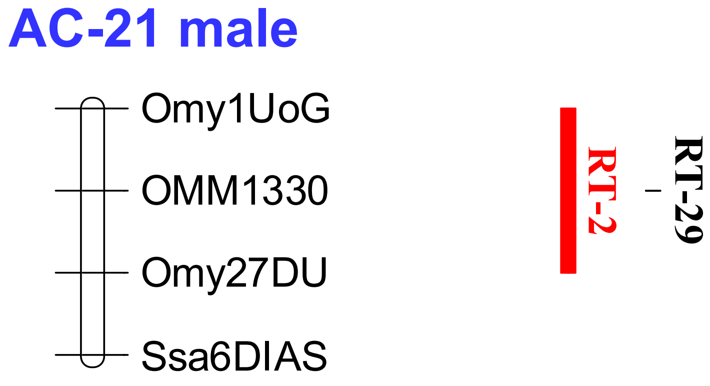
AC-21 male
Omy1UoG
OMM1330
Omy27DU
Ssa6DIAS
RT-2
RT-29

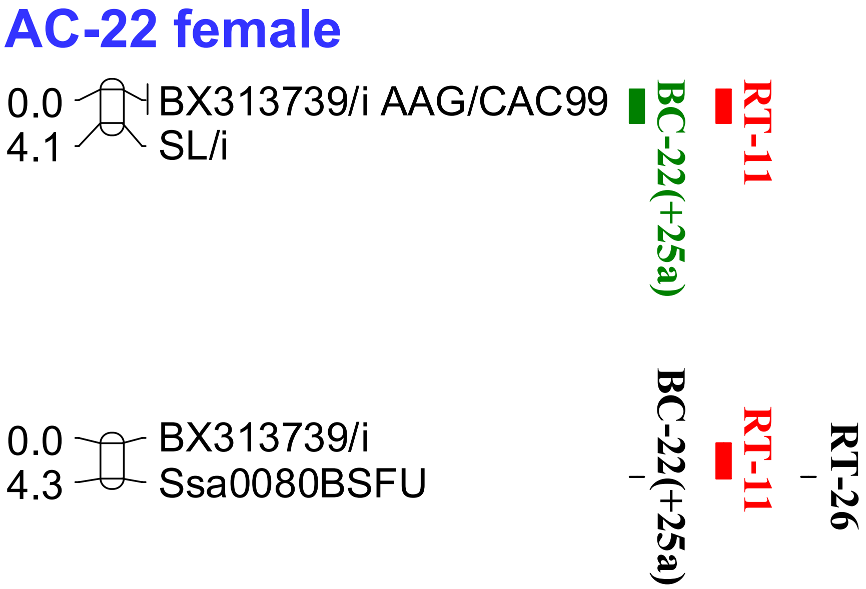
AC-22 female
0.0
4.1
BX313739/i AAG/CAC99
SL/i
BC-22(+25a)
RT-11
0.0
4.3
BX313739/i
Ssa0080BSFU
BC-22(+25a)
RT-11
RT-26

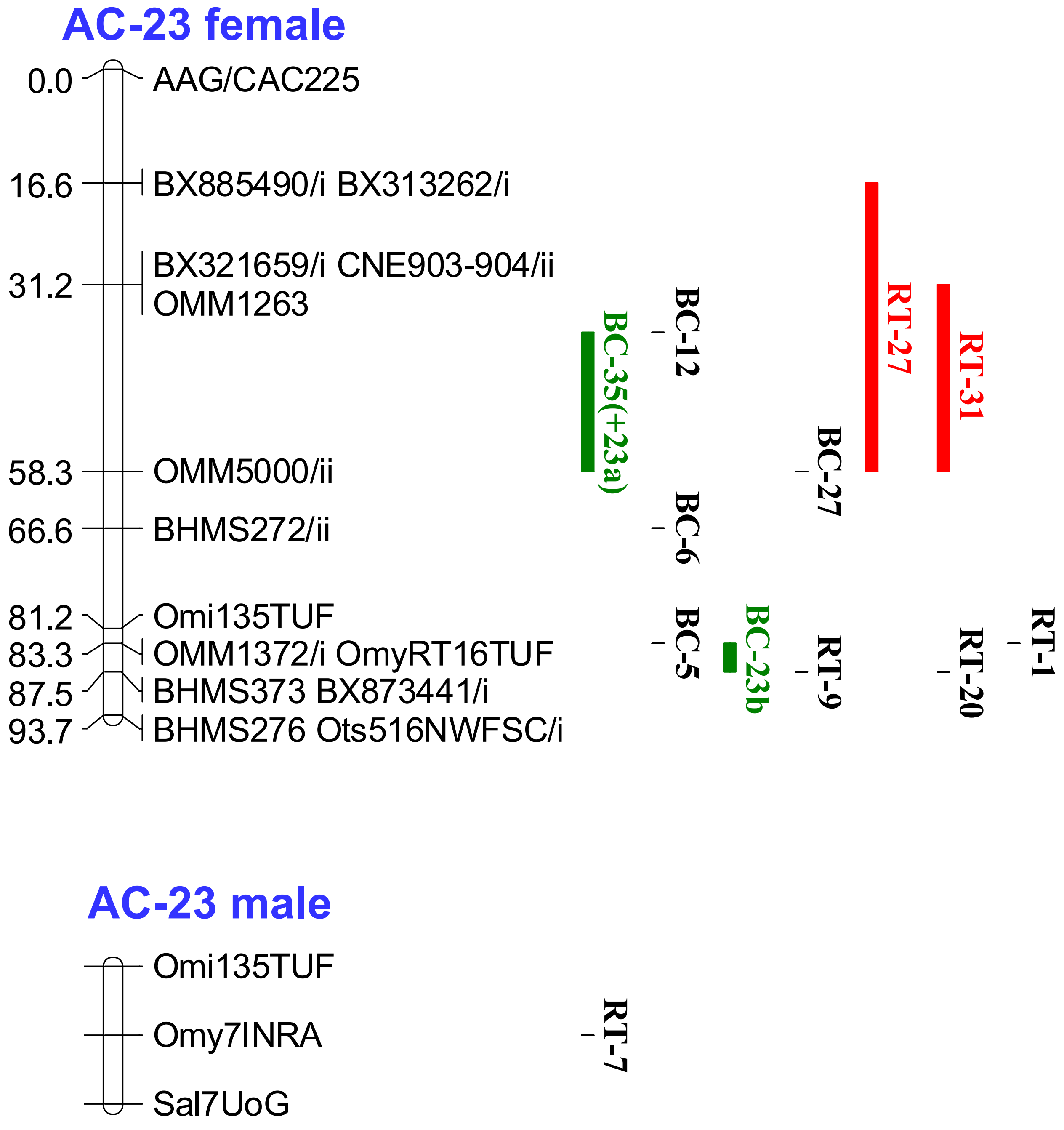
AC-23 female
0.0 AAG/CAC225
16.6 BX885490/i BX313262/i
31.2 BX321659/i CNE903-904/ii
OMM1263
58.3 OMM5000/ii
66.6 BHMS272/ii
81.2 Omi135TUF
83.3 OMM1372/i OmyRT16TUF
87.5 BHMS373 BX873441/i
93.7 BHMS276 Ots516NWFSC/i
BC-35(+23a)
BC-12
BC-6
BC-5
BC-23b
BC-27
RT-9
RT-27
RT-31
RT-20
RT-1
AC-23 male
Omi135TUF
Omy7INRA
Sal7UoG
RT-7

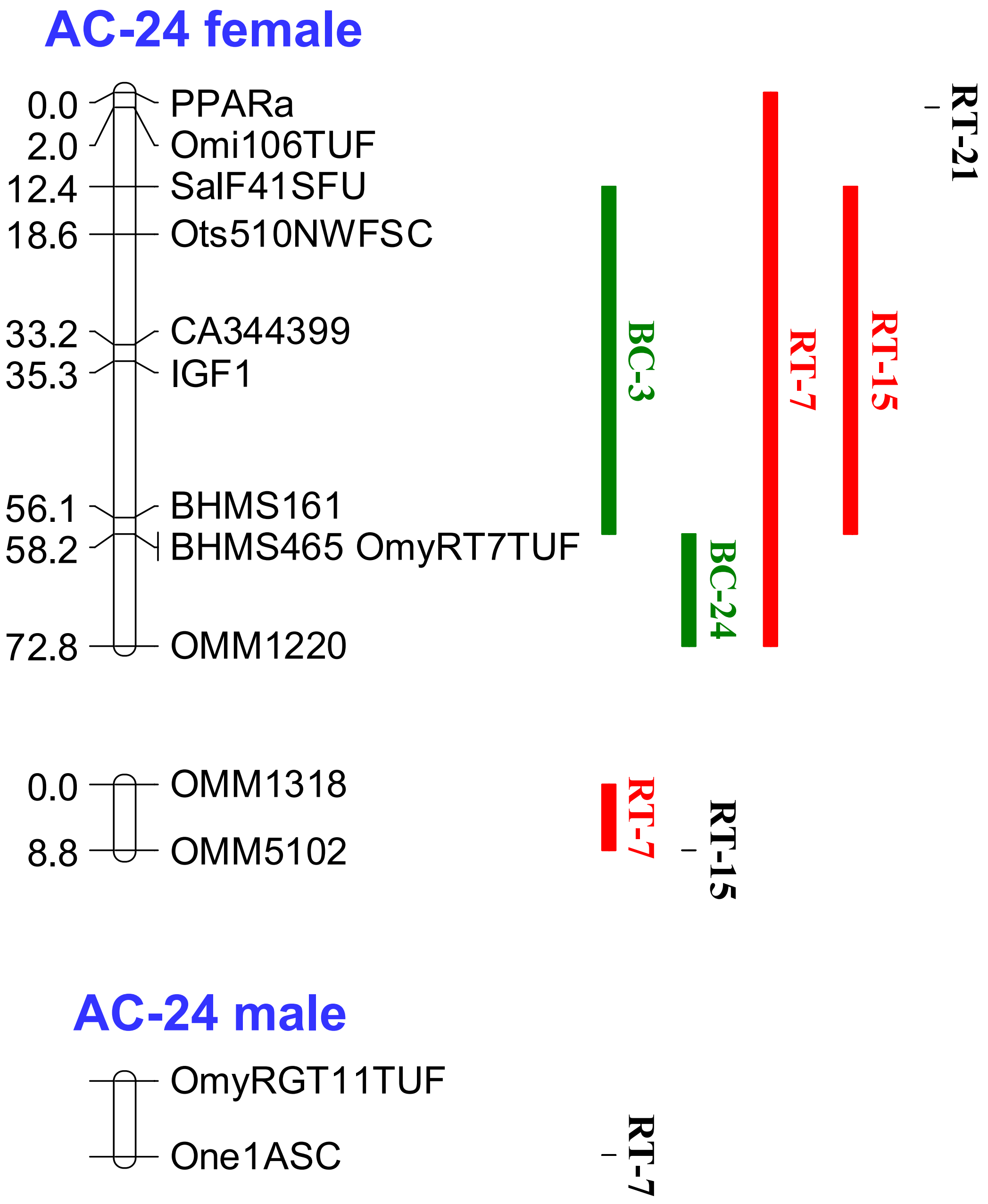
AC-24 female
0.0 PPARa
2.0 Omi106TUF
12.4 SalF41SFU
18.6 Ots510NWFSC
33.2 CA344399
35.3 IGF1
56.1 BHMS161
58.2 BHMS465 OmyRT7TUF
72.8 OMM1220
BC-3
BC-24
RT-7
RT-15
RT-21
0.0 OMM1318
8.8 OMM5102
RT-7
RT-15
AC-24 male
OmyRGT11TUF
One1ASC
RT-7

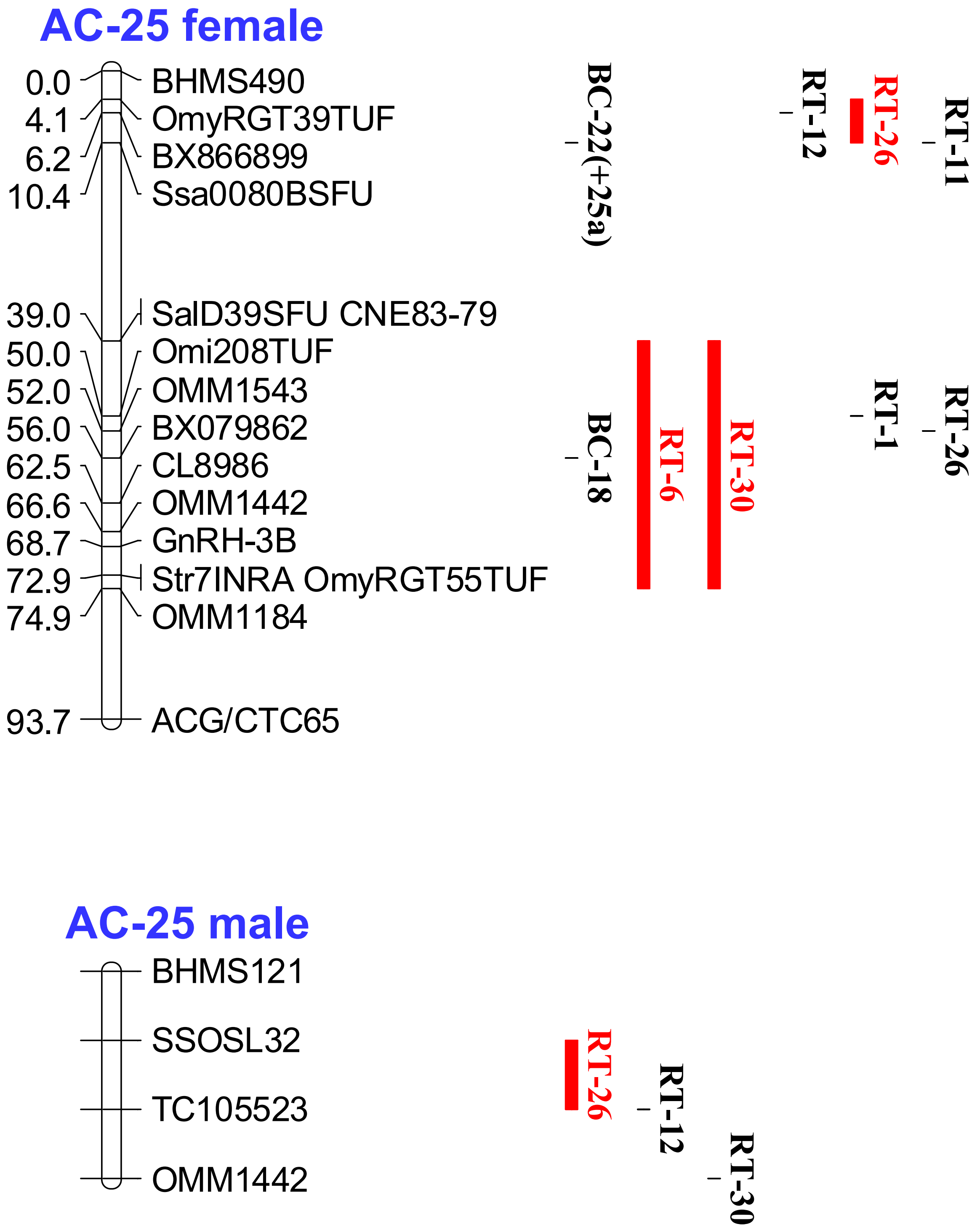

AC-25 female
0.0
4.1
6.2
10.4
39.0
50.0
52.0
56.0
62.5
66.6
68.7
72.9
74.9
93.7
BHMS490
OmyRGT39TUF
BX866899
Ssa0080BSFU
SalD39SFU CNE83-79
Omi208TUF
OMM1543
BX079862
CL8986
OMM1442
GnRH-3B
Str7INRA OmyRGT55TUF
OMM1184
ACG/CTC65
BC-22(+25a)
RT-12
RT-26
RT-11
BC-18
RT-6
RT-30
RT-1
RT-26
AC-25 male
BHMS121
SSOSL32
TC105523
OMM1442
RT-26
RT-12
RT-30

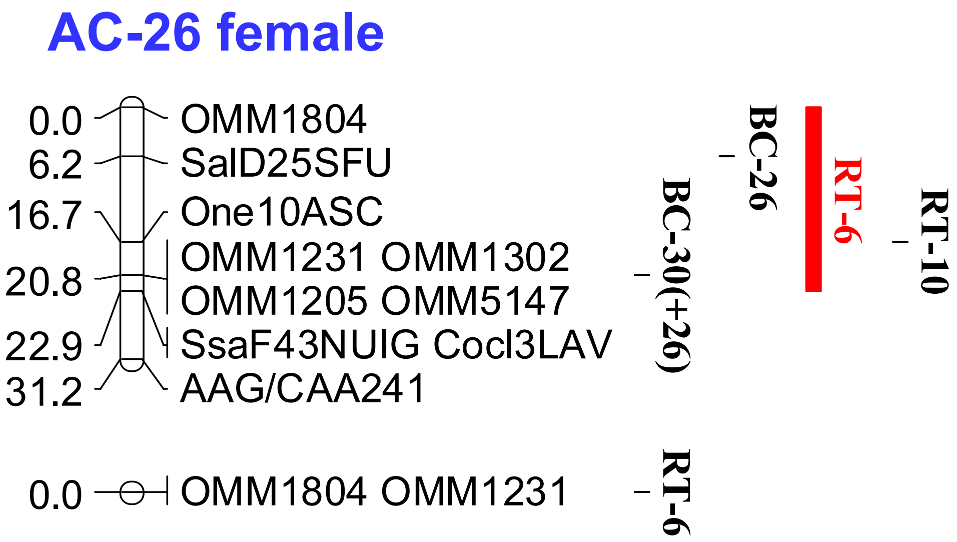
AC-26 female
0.0
6.2
16.7
20.8
22.9
31.2
OMM1804
SalD25SFU
One10ASC
OMM1231 OMM1302
OMM1205 OMM5147
SsaF43NUIG Cocl3LAV
AAG/CAA241
0.0
OMM1804 OMM1231
BC-26
RT-6
RT-10
BC-30(+26)
RT-6

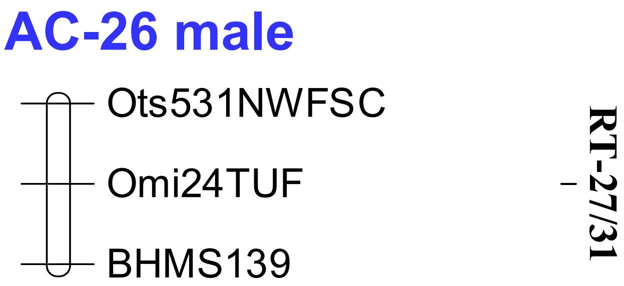
AC-26 male
Ots531NWFSC
Omi24TUF
BHMS139
RT-27/31

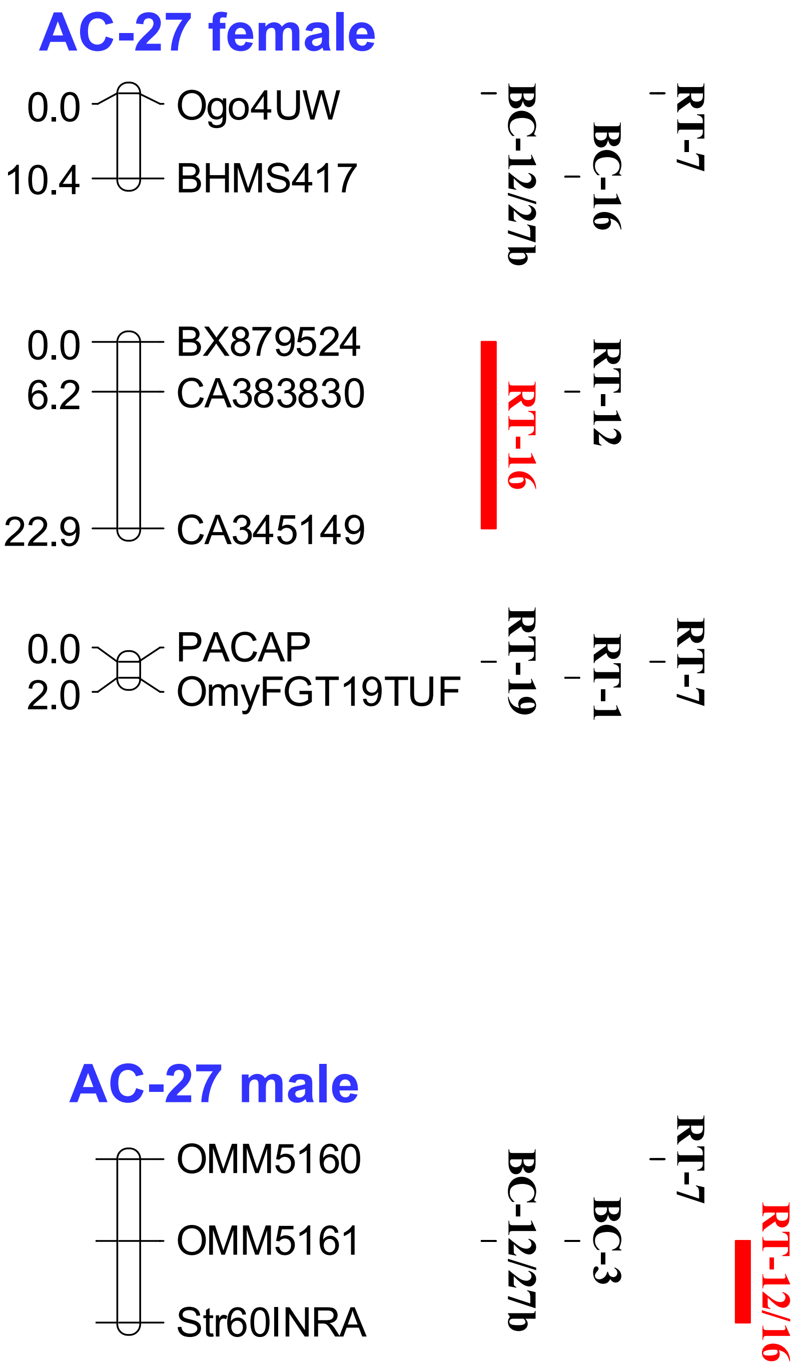
AC-27 female
0.0
Ogo4UW
10.4
BHMS417
BC-12/27b
BC-16
RT-7
0.0
BX879524
6.2
CA383830
22.9
CA345149
RT-16
RT-12
0.0
PACAP
2.0
OmyFGT19TUF
RT-19
RT-1
RT-7
AC-27 male
OMM5160
OMM5161
Str60INRA
BC-12/27b
BC-3
RT-7
RT-12/16

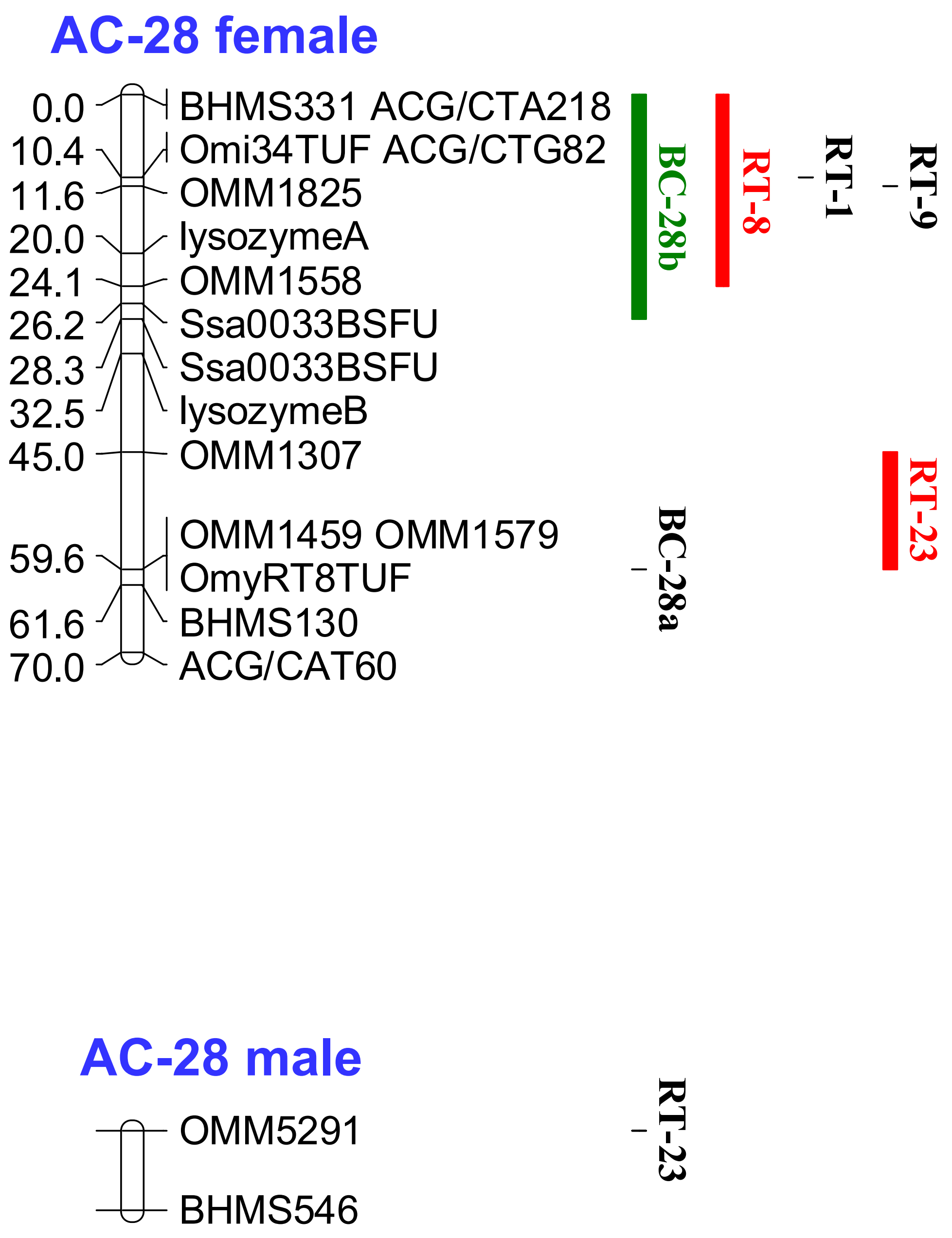
AC-28 female
0.0 BHMS331 ACG/CTA218
10.4 Omi34TUF ACG/CTG82
11.6 OMM1825
20.0 lysozymeA
24.1 OMM1558
26.2 Ssa0033BSFU
28.3 Ssa0033BSFU
32.5 lysozymeB
45.0 OMM1307
OMM1459 OMM1579
59.6 OmyRT8TUF
61.6 BHMS130
70.0 ACG/CAT60
BC-28b
RT-8
RT-1
RT-9
RT-23
BC-28a
AC-28 male
OMM5291
BHMS546
RT-23

AC-30 female

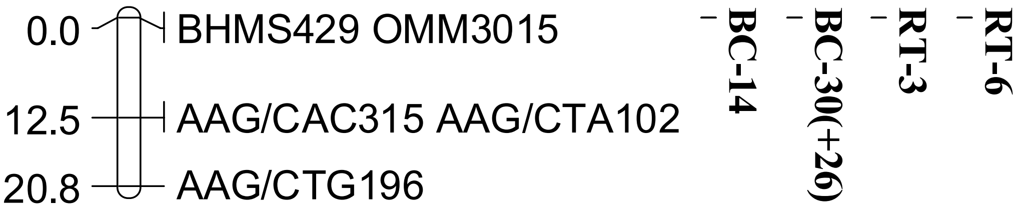

0.0
BHMS429 OMM3015
12.5
AAG/CAC315 AAG/CTA102
20.8
AAG/CTG196
BC-14
BC-30(+26)
RT-3
RT-6

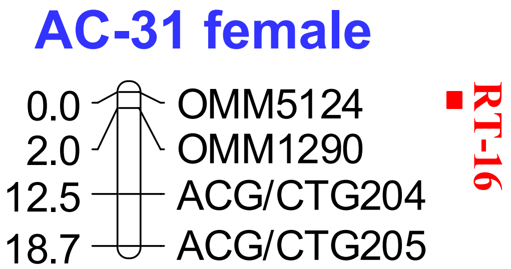
AC-31 female
0.0
2.0
12.5
18.7
OMM5124
OMM1290
ACG/CTG204
ACG/CTG205
RT-16

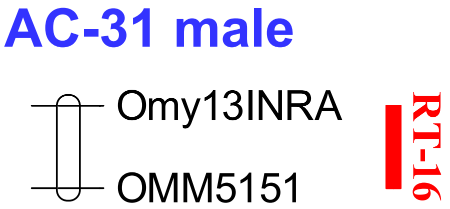
AC-31 male
Omy13INRA
OMM5151
RT-16

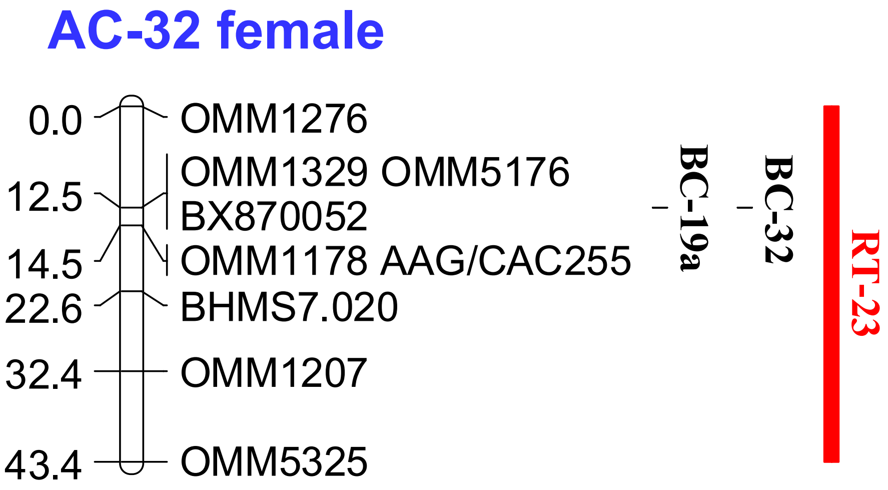

AC-32 female
0.0
OMM1276
12.5
OMM1329 OMM5176
BX870052
14.5
OMM1178 AAG/CAC255
22.6
BHMS7.020
32.4
OMM1207
43.4
OMM5325
BC-19a
BC-32
RT-23

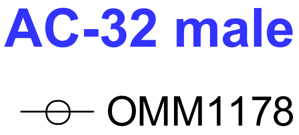

AC-32 male
OMM1178

AC-33 female

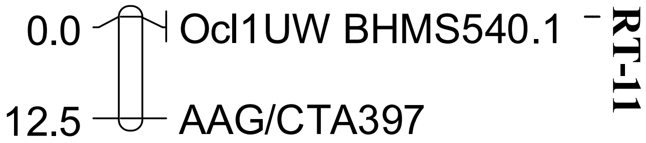

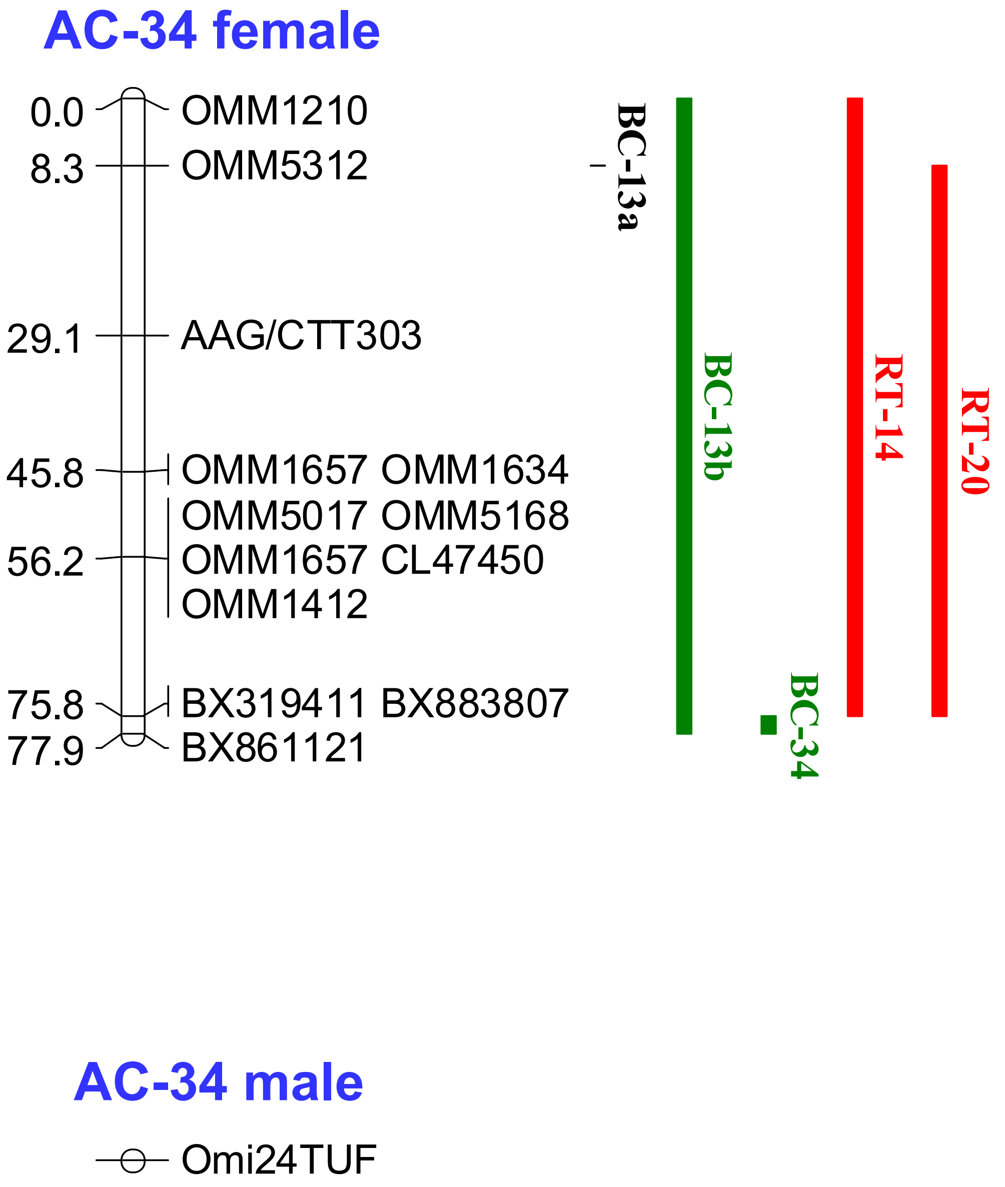

AC-34 female
0.0
8.3
29.1
45.8
56.2
75.8
77.9
OMM1210
OMM5312
AAG/CTT303
OMM1657 OMM1634
OMM5017 OMM5168
OMM1657 CL47450
OMM1412
BX319411 BX883807
BX861121
BC-13a
BC-13b
BC-34
RT-14
RT-20
AC-34 male
Omi24TUF

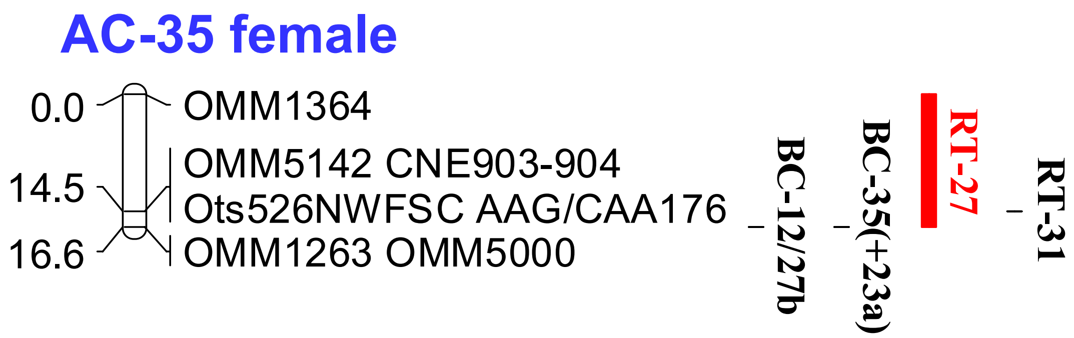
AC-35 female
0.0
14.5
16.6
OMM1364
OMM5142 CNE903-904
Ots526NWFSC AAG/CAA176
OMM1263 OMM5000
BC-12/27b
BC-35(+23a)
RT-27
RT-31

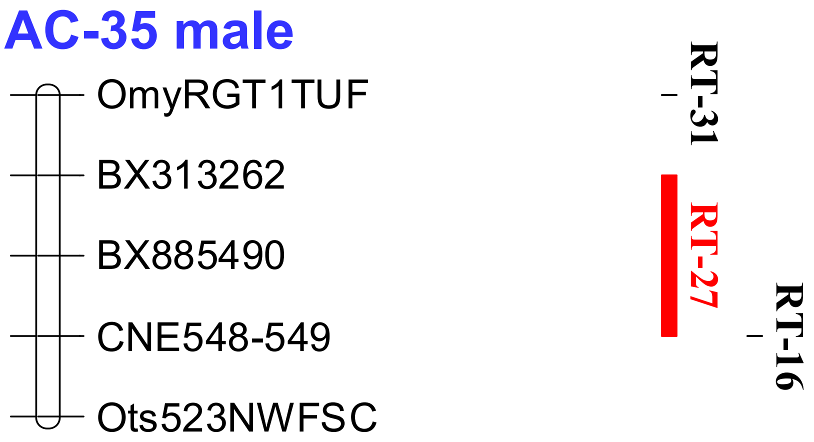
AC-35 male
OmyRGT1TUF
BX313262
BX885490
CNE548-549
Ots523NWFSC
RT-31
RT-27
RT-16

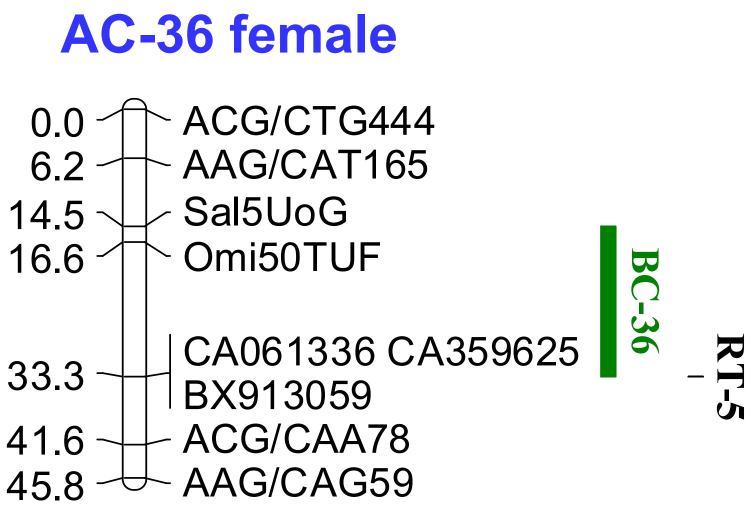
AC-36 female
0.0 ACG/CTG444
6.2 AAG/CAT165
14.5 Sal5UoG
16.6 Omi50TUF
33.3 CA061336 CA359625 BX913059
41.6 ACG/CAA78
45.8 AAG/CAG59
BC-36
RT-5

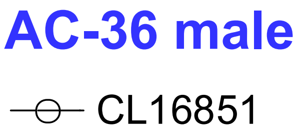
AC-36 male
CL16851

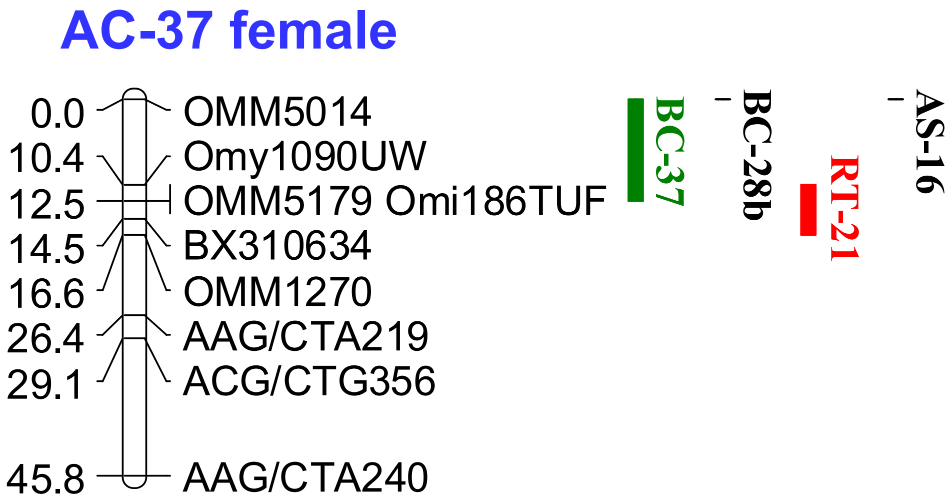
AC-37 female
0.0
10.4
12.5
14.5
16.6
26.4
29.1
45.8
OMM5014
Omy1090UW
OMM5179 Omi186TUF
BX310634
OMM1270
AAG/CTA219
ACG/CTG356
AAG/CTA240
BC-37
BC-28b
RT-21
AS-16

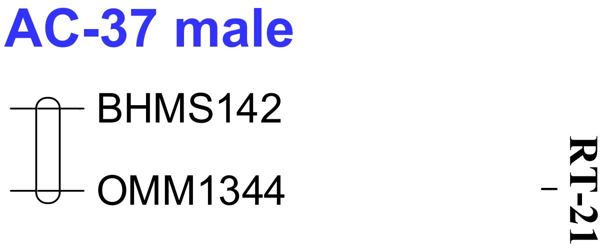
AC-37 male
BHMS142
OMM1344
RT-21

AC-39 female

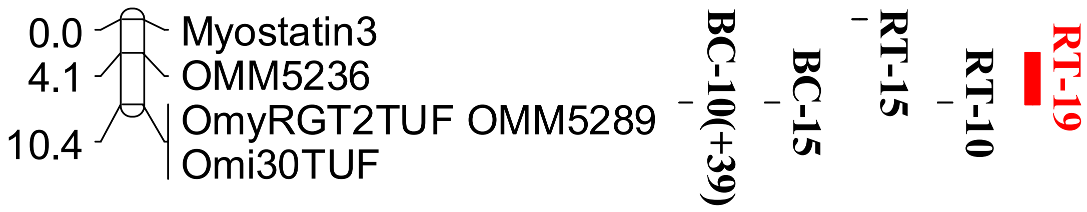

AC-39 male

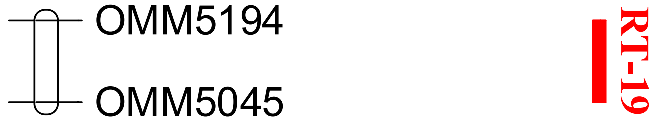

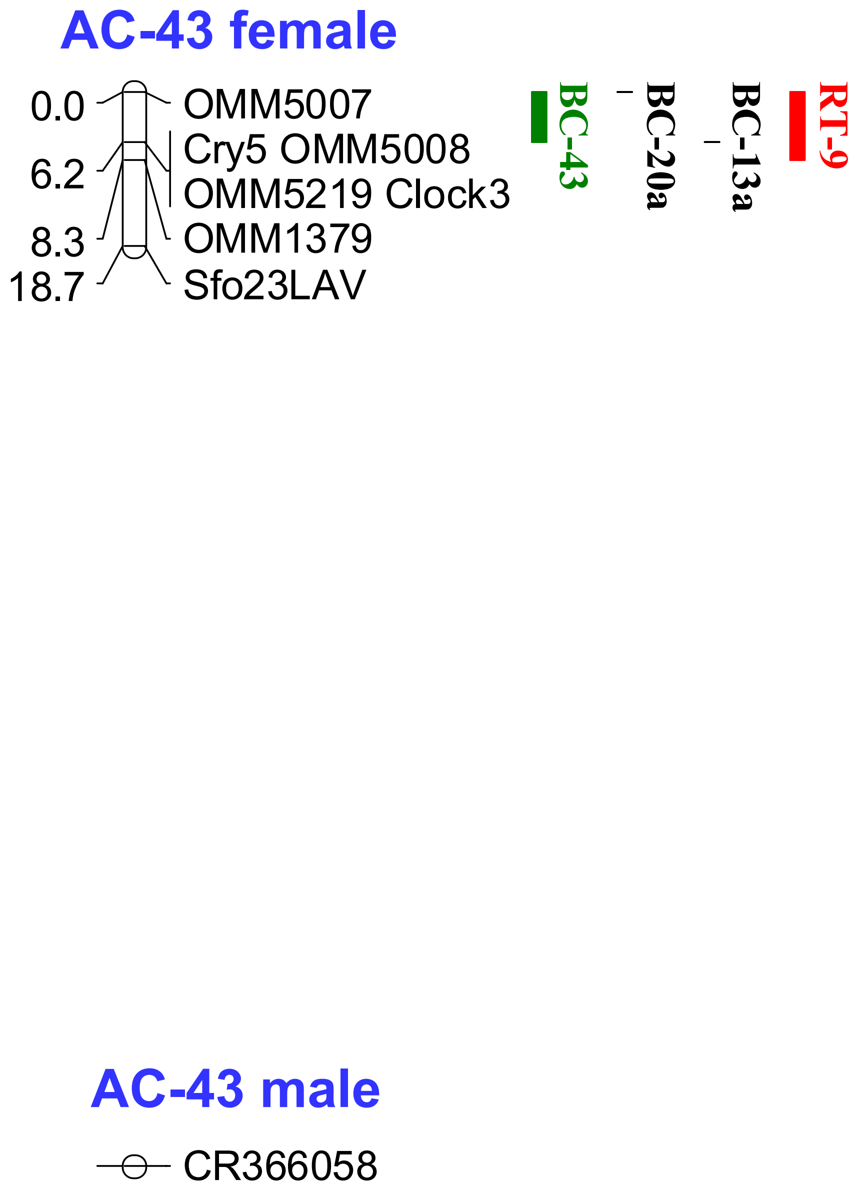
AC-43 female
0.0
6.2
8.3
18.7
OMM5007
Cry5 OMM5008
OMM5219 Clock3
OMM1379
Sfo23LAV
BC-43
BC-20a
BC-13a
RT-9
AC-43 male
CR366058
