# Supplementary material for: Genome evolution in the fish family salmonidae: generation of a brook charr genetic map and comparisons among charrs (Arctic charr and brook charr) with rainbow trout
Source: BMC Genet. 2011 Jul 28;12:68. doi: 10.1186/1471-2156-12-68 (PMC3162921; doi:10.1186/1471-2156-12-68)
Supplement: Additional file 10 — Complete listing of all the genetic markers screened in the brook charr mapping panels. [file 1471-2156-12-68-S10.DOC]

Additional File 10. Compendium of markers genotyped in brook charr including information on the strain of origin for the mapping families (HL = Hills Lake; LN = Nipigon), whether the marker was polymorphic (P), monomorophic (M), unresolved (?), or did not amplify (NA) in an initial screening of the HL3, HL7 and LN4 mapping parents. If mapping parents were identical heterozygotes, only half of the progeny provided informative genotypes within the family and these markers are indicated as: (0.5). Markers are listed in alphabetical order.

| Primer Name | HL3F | HL3M | HL7F | HL7M | LN4F | LN4M |
| --- | --- | --- | --- | --- | --- | --- |
| 1A2S2R | M | M | M | M | M | M |
| 1A4H2F | M | M | M | M | M | M |
| 1B442F | M | M | M | M | 0.5 | 0.5 |
| BX299451 | 0.5 | 0.5 | 0.5 | 0.5 | M | M |
| BG934221 | M | M | M | M | M | M |
| BHMS130 | ? | ? | ? | ? | ? | ? |
| BHMS206 | P | P | P | P | P | P |
| BHMS238 | P | M | P | P | M | P |
| BHMS272 | P | P | P | P | P? | P? |
| BHMS330 | P | M | M | M | M | M |
| BHMS331 | P | M | P | M | 0.5 | 0.5 |
| BHMS377 | P | P | P | P | M | M |
| BHMS411 | P | M | M | M | M | M |
| BHMS417 | P | P | P | P | M | M |
| BHMS429 | M | P | P | M | M | M |
| BHMS465 | P | P | P | P | P | P |
| BHMS490 | P | P | M | P | M | M |
| BHMS7.001 | M | M | M | M | M | M |
| BHMS7.011 | 0.5 | 0.5 | P | M | M | M |
| BHMS7.030 | ? | ? | ? | ? | ? | ? |
| BHMS7.036 | ? | ? | ? | ? | ? | ? |
| BX073647 | P | P | P | P | M | M |
| BX073974 | P | M | M | P | 0.5 | 0.5 |
| BX076085 | M | P | M | M | M | M |
| BX079862 | M | P | M | P | 0.5 | 0.5 |
| BX080247 | ? | ? | ? | ? | ? | ? |
| BX087219 | M | M | M | M | M | M |
| BX087664 | P | P | M | M | 0.5 | 0.5 |
| BX296674 | ? | ? | ? | ? | ? | ? |
| BX302949 | ? | ? | ? | ? | ? | ? |
| BX303525 | M | M | M | M | M | M |
| BX305863 | ? | ? | ? | ? | ? | ? |
| BX309199 | P | M | M | P | M | M |
| BX310634 | P | M | P | P | ? | ? |
| BX311224 | P | P | P | M | P | P |
| BX311884 | P | P | P | P | M | P |
| BX313262 | ? | ? | ? | ? | ? | ? |
| BX313739 | M | P | P | M | M | M |
| BX318599 | M | M | P | P | M | M |
| BX319197 | P | P | P | P | P | M |
| Primer Name | HL3F | HL3M | HL7F | HL7M | LN4F | LN4M |
| BX319411 | P | P | P | P | ? | ? |
| BX320910 | M | M | M | M | M | M |
| BX321659 | M | M | M | P | P | P |
| BX859491 | M | M | M | M | M | M |
| BX861121 | P | P | P | P | ? | ? |
| BX863758 | P | M | M | P | M | M |
| BX866899 | M | M | M | M | M | M |
| BX867246 | ? | ? | ? | ? | ? | ? |
| BX870052 | P | P | P | P | M | M |
| BX873441 | P | P | P | P | P | M |
| BX873975 | M | M | M | M | M | M |
| BX879524 | M | M | M | M | M | M |
| BX881655 | P | P | P | P | M | M |
| BX883807 | ? | ? | ? | ? | ? | ? |
| BX885490 | ? | ? | ? | ? | ? | ? |
| BX890355 | P | P | P | P | P | M |
| BX913059 | M | M | M | M | M | M |
| CA039635 | M | M | M | M | M | M |
| CA039640 | M | M | M | M | M | M |
| CA050694 | ? | P | M | M | M | M |
| CA060381 | P | P | 0.5 | 0.5 | M | M |
| CA061336 | P | P | P | P | P | M |
| CA344270 | M | P | P | P | M | M |
| CA345149 | P | P | P | P | P | P |
| CA349175 | M | M | M | M | M | M |
| CA350064 | P | M | M | M | P | M |
| CA359625 | P | P | M | M | M | M |
| CA365390 | M | M | M | P | M | M |
| CA368462 | M | P | P | P | P | P |
| CA375845 | M | M | M | M | M | M |
| CA376300 | M | M | P | P | M | M |
| CA378164 | P | M | M | M | P | M |
| CA379795 | P | M | 0.5 | 0.5 | M | M |
| CA383830 | ? | ? | ? | ? | ? | ? |
| CB512520 | ? | ? | ? | ? | ? | ? |
| CL4778 | M | P | P | M | M | M |
| CL8986 | ? | ? | ? | ? | ? | ? |
| CL15841 | M | M | M | M | M | M |
| CL18423 | ? | ? | ? | ? | ? | ? |
| CL47450 | 0.5 | 0.5 | 0.5 | 0.5 | M | M |
| CL67680 | M | M | M | M | M | M |
| CLK3-2B1F2R2 | M | M | M | M | M | M |
| CLK3-2B1R4F4 | M | M | M | M | M | M |
| CLK3-2B2C3R1F1 | M | M | M | M | M | M |
| CLOCK3-7C2F1R1 | M | M | M | M | M | M |
| CLOCK3-7C2R3F3 | M | P | M | M | P | P |
| CR363293 | P | P | M | P | M | M |
| Primer Name | HL3F | HL3M | HL7F | HL7M | LN4F | LN4M |
| NPAS2-6B1F2R2 | M | M | M | M | M | M |
| NPAS2-6B1F2R2 | M | M | M | M | M | M |
| Ogo3UW | M | M | M | M | M | M |
| Ogo4UW | P | P | P | P | M | M |
| Ogo8UW | M | M | M | M | M | M |
| Omi106TUF | ? | ? | ? | ? | ? | ? |
| Omi120TUF | M | M | M | P | M | M |
| Omi126TUF | P | M | P | P | M | P |
| Omi127TUF | ? | ? | ? | ? | ? | ? |
| Omi179TUF | P | P | P | M | M | M |
| Omi208TUF | ? | ? | ? | ? | ? | ? |
| Omi30TUF | P | P | P | P | P | M |
| OMM1075 | ? | ? | ? | ? | ? | ? |
| OMM1135 | M | M | M | M | M | M |
| OMM1178 | M | P | M | M | M | M |
| OMM1184 | P | M | M | P | M | M |
| OMM1195 | P | P | P | P | M | P |
| OMM1197 | P | P | P | P | P | P |
| OMM1205 | P | P | P | M | M | M |
| OMM1207 | M | P | P | M | M | M |
| OMM1210 | M | P | P | P | M | M |
| OMM1211 | M | M | P | M | P | M |
| OMM1220 | P | P | P | P | P | P |
| OMM1228 | P | P | P | P | M | M |
| OMM1231 | NA | NA | NA | NA | NA | NA |
| OMM1237 | ? | ? | ? | ? | ? | ? |
| OMM1238 | M | P | M | M | M | M |
| OMM1263 | P | ? | P | P | M | M |
| OMM1268 | NA | NA | NA | NA | NA | NA |
| OMM1270 | M | P | M | M | M | M |
| OMM1276 | 0.5 | 0.5 | 0.5 | 0.5 | M | M |
| OMM1290 | P | P | M | ? | M | M |
| OMM1300 | ? | M | M | P | P | M |
| OMM1304 | M | M | M | M | M | M |
| OMM1306 | ? | ? | ? | ? | ? | ? |
| OMM1307 | M | M | M | M | M | M |
| OMM1308 | P | P | P | P | ? | ? |
| OMM1312 | P | P | P | P | P | P |
| OMM1318 | ? | ? | ? | ? | ? | ? |
| OMM1321 | M | M | M | M | M | M |
| OMM1329 | P | M | P | P | 0.5 | 0.5 |
| OMM1345 | M | M | P | P | M | M |
| OMM1364 | ? | ? | ? | ? | ? | ? |
| OMM1374 | ? | ? | ? | ? | ? | ? |
| OMM1442 | M | M | M | P | M | P? |
| OMM1445 | M | P | P | P | M | M |
| OMM1459 | M | M | P | M | P | M |
| Primer Name | HL3F | HL3M | HL7F | HL7M | LN4F | LN4M |
| OMM1512 | M | P | M | P | M | M |
| OMM1543 | NA | NA | NA | NA | NA | NA |
| OMM1579 | M | P | P | P | M | M |
| OMM1804 | M | P | P | P | M | P |
| OMM3015 | P | M | P | M | M | M |
| OMM3075 | ? | ? | P | P | M | M |
| OMM3095 | P | P | 0.5 | 0.5 | M | P |
| OMM5000 | P | P | P | P | P | P |
| OMM5007 | P | P | P | M | M | M |
| OMM5008 | P | M | P | P | P | P |
| OMM5009 | M | M | M | M | M | M |
| OMM5011 | M | M | M | M | M | M |
| OMM5013 | M | M | M | M | M | M |
| OMM5014 | P | P | M | P | M | P |
| OMM5018 | 0.5 | 0.5 | P | M | M | M |
| OMM5019 | P | P | P | P | M | P |
| OMM5024 | P | M | M | M | M | M |
| OMM5035 | M | M | M | M | M | M |
| OMM5051 | M | M | M | M | M | M |
| OMM5053 | M | M | M | M | M | M |
| OMM5056 | P | P | P | P | M | M |
| OMM5060 | M | M | P | M | M | M |
| OMM5061 | P | P | P | ? | M | P |
| OMM5063 | ? | ? | ? | ? | ? | ? |
| OMM5074 | M | M | M | P | M | M |
| OMM5091 | P | M | P | P | M | P |
| OMM5092 | P | M | M | M | M | M |
| OMM5102 | P | P | P | P | M | M |
| OMM5113 | P | P | P | M | M | P |
| OMM5124 | NA | NA | NA | NA | NA | NA |
| OMM5132 | NA | NA | NA | NA | NA | NA |
| OMM5133 | M | M | M | M | M | M |
| OMM5139 | ? | ? | ? | ? | ? | ? |
| OMM5142 | P | M | M | M | M | M |
| OMM5146 | ? | ? | ? | ? | ? | ? |
| OMM5147 | M | M | P | M | M | M |
| OMM5149 | M | M | M | P | M | M |
| OMM5154 | ? | ? | ? | ? | ? | ? |
| OMM5155 | M | M | M | P | M | M |
| OMM5157 | M | M | P | M | M | M |
| OMM5161 | P | P | P | M | P | M |
| OMM5168 | ? | ? | ? | ? | M | M |
| OMM5172 | M | M | M | M | M | M |
| OMM5175 | M | M | M | M | M | M |
| OMM5176 | P | P | P | P | M | M |
| OMM5179 | P | P | M | M | M | M |
| OMM5181 | M | M | M | M | M | M |
| Primer Name | HL3F | HL3M | HL7F | HL7M | LN4F | LN4M |
| OMM5182 | M | M | M | M | M | M |
| OMM5185 | M | M | M | M | M | M |
| OMM5236 | M | M | M | M | M | M |
| OMM5265 | P | M | P | P | ? | P |
| OMM5289 | M | M | M | M | M | M |
| OMM5295 | ? | ? | ? | ? | ? | ? |
| OMM5312 | P | P | P | P | P | P |
| OMM5321 | M | M | M | M | M | M |
| Omy1339INRA | 0.5 | 0.5 | M | M | M | M |
| Omy21IRNA | P | P | P | P | P | P |
| Omy6DIAS | P | M | M | M | M | M |
| OmyRGT2TUF | P | P | P | P | M | M |
| OmyRGT35TUF | M | M | M | M | M | M |
| OmyRGT6TUF | ? | ? | ? | ? | ? | ? |
| OmyRT16TUF | P | P | P | P | M | P |
| OmyRT7TUF | M | M | M | M | M | M |
| One8ASC | M | M | M | M | M | P |
| Ots2BML | ? | ? | ? | ? | ? | ? |
| Ots3BML | ? | ? | ? | ? | ? | ? |
| Ots516NWFSC | 0.5 | 0.5 | P | P | P | M |
| Ots53INWFSC | ? | ? | ? | ? | ? | ? |
| Sal5UOG | P | M | P | P | 0.5 | 0.5 |
| Sal16UOG | M | M | M | M | M | M |
| Sal9UOG | P | P | M | M | M | M |
| SalD25SFU | M | P | M | P | 0.5 | 0.5 |
| SalD39SFU | M | P | P | P | 0.5 | 0.5 |
| SalE38SFU | P | P | M | P | P | P |
| SalF41SFU | P | P | P | P | M | P |
| SalO23SFU | ? | ? | ? | ? | ? | ? |
| Sfo23LAV | M | M | M | M | M | M |
| OkeSLINRA | P | P | P | M | M | P |
| SmaBFR01 | ? | ? | ? | ? | ? | ? |
| Ssa17 | P | P | M | P | M | M |
| Ssa33BSFU | P | P | P | P | M | P |
| Ssa3DIAS | M | M | M | M | M | M |
| Ssa54BSFU | M | M | M | M | M | M |
| Ssa72BSFU | P | P | P | P | P | P |
| Ssa80BSFU | P | P | P | P | M | M |
| Ssa87BSFU | M | P | 0.5 | 0.5 | M | M |
| SsoSL456 | ? | ? | ? | ? | ? | ? |
| Str7INRA | M | M | M | P | M | M |
| TC105189 | P | P | M | M | M | M |
| TC105523 | ? | ? | ? | ? | ? | ? |
| TC126859 | P | P | M | P | P | P |
| TC128101 | ? | ? | ? | ? | ? | ? |
